# Telomerase reverse transcriptase gene knock‐in unleashes enhanced longevity and accelerated damage repair in mice

**DOI:** 10.1111/acel.14445

**Published:** 2024-12-11

**Authors:** Tian‐Yi Zhu, Po Hu, Yu‐Hui Mi, Jun‐Li Zhang, An‐Na Xu, Ming‐Tong Gao, Ying‐Ying Zhang, San‐Bing Shen, Guang‐Ming Yang, Yang Pan

**Affiliations:** ^1^ School of Pharmacy Nanjing University of Chinese Medicine Nanjing Jiangsu China; ^2^ Clem Jones Centre for Ageing Dementia Research, Queensland Brain Institute The University of Queensland Brisbane Queensland Australia; ^3^ Regenerative Medicine Institute, School of Medicine University of Galway Galway Ireland

**Keywords:** damage repair, lifespan extension, telomerase reverse transcriptase, *Tert* knock‐in, transgenic mice

## Abstract

While previous research has demonstrated the therapeutic efficacy of telomerase reverse transcriptase (TERT) overexpression using adeno‐associated virus and cytomegalovirus vectors to combat aging, the broader implications of TERT germline gene editing on the mammalian genome, proteomic composition, phenotypes, lifespan extension, and damage repair remain largely unexplored. In this study, we elucidate the functional properties of transgenic mice carrying the *Tert* transgene, guided by precise gene targeting into the Rosa26 locus via embryonic stem (ES) cells under the control of the elongation factor 1α (*EF1α*) promoter. The *Tert* knock‐in (*TertKI*) mice harboring the *EF1α‐Tert* gene displayed elevated telomerase activity, elongated telomeres, and extended lifespan, with no spontaneous genotoxicity or carcinogenicity. The *TertKI* mice showed also enhanced wound healing, characterized by significantly increased expression of Fgf7, Vegf, and collagen. Additionally, *TertKI* mice exhibited robust resistance to the progression of colitis induced by dextran sodium sulfate (DSS), accompanied by reduced expression of disease‐deteriorating genes. These findings foreshadow the potential of *TertKI* as an extraordinary rejuvenation force, promising not only longevity but also rejuvenation in skin and intestinal aging.

## Graphical abstract

Tert knock‐in (*TertKI*) mice harboring the *EF1α‐Tert* gene in the *Rosa26* locus displayed heightened telomerase activity, elongated telomeres, and extended lifespan. *TertKI* mice displayed enhanced wound healing and exhibited resistance to the progression of colitis.

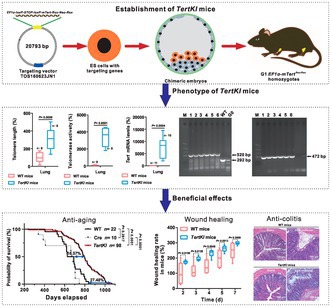

**Abbreviations**
AA: amino acid
BAC: bacterial artificial chromosome
bp: base pairs
CA72‐4: carbohydrate antigen 72‐4
CAG: cytosine‐adenine‐guanine
DSS: dextran sodium sulfate
DMAB: dimethylaminobenzaldehyde
EF1α: elongation factor 1α
ES: embryonic stem
ECM: extracellular matrix
Got1: glutamic‐oxaloacetic transaminase
Gpt: glutamic‐pyruvic transaminase
GSH: glutathione
H&E: hematoxylin and eosin
HYP: hydroxyproline
Il1β: interleukin 1 beta
mg/g: milligrams per gram
mTert: mouse Tert
ANOVA: one‐way analysis of variance
PCR: polymerase chain reaction
SOD: superoxide dismutase
TAC: telomerase activating compound
TERT: telomerase reverse transcriptase
Terc: telomerase RNA component
TertKI: Tert knock‐in
Tgf‐β1: transforming growth factor beta
Tnfα: tumor necrosis factor alpha
WT: wild‐type

## 1 INTRODUCTION

Extensive research on aging has identified over 1000 genes associated with aging in various model organisms (Guarente & Kenyon, 2000; Tacutu et al., 2018), making genetic interventions promising for extending lifespan (Vaiserman et al., 2019). Telomeres, the DNA sequences at the ends of chromosomes, undergo shortening during cellular aging (Harley et al., 1990), and the discovery of telomerase, an enzyme that maintains telomere length (Poole et al., 2001; Shampay & Blackburn, 1988), suggests its potential use for enhancing cell longevity (Varela & Blasco, 2010). Telomerase reverse transcriptase (TERT), encoded by the *Tert* gene in mice (*TERT* in humans), plays a vital role in this process (Weinrich et al., 1997), and its introduction in human cells is shown to maintain telomere length and cellular immortality (Bodnar et al., 1998).

Mice, due to their genetic similarity to humans and genetic manipulability, serve as valuable models in aging research (Vanhooren & Libert, 2013). Studies involving extended telomeres in mice showed potential benefits for longevity and health (Muñoz‐Lorente et al., 2019). Another study showed that human‐specific regulatory elements in the *hTERT* promoter could explain differences in telomerase expression between humans and mice, highlighting the importance of creating humanized models for TERT research (Horikawa et al., 2005), and transgenic mice generated with a *hTERT* gene harboring a luciferase reporter element was used for screening drugs which up‐regulate human TERT expression (Jia et al., 2011; Shim et al., 2024). While various methods, such as broad‐spectrum Tert‐expressing viruses and exogenous *Tert* introduction, have showed promising results in extending lifespan without increased cancer risk or other adverse effects (Bernardes de Jesus et al., 2012; Jaijyan et al., 2022; Mojiri et al., 2021; Tomás‐Loba et al., 2008), there are still limitations. For instance, the use of viruses or exogenous *Tert* introduction may raise concerns about safety and control, potentially leading to unintended effects or immune responses. In contrast, the utilization of non‐viral promoters to drive *Tert* expression represents a potentially safer and more controllable approach for treatment. This innovative strategy capitalizes on recent advancements in genome‐targeting technologies in mouse models (Skelton et al., 2013), thereby enhancing the heritability, precision, and efficiency of gene therapy (Wolf et al., 2019). Additionally, embryonic stem (ES) cell targeting is highly efficient for creating complex genetic modifications, such as large DNA knock‐ins, which are challenging to achieve with CRISPR/Cas9 due to lower success rates for large insertions, and it enables high‐throughput in vitro screening and targeting (Ozawa et al., 2023).

Despite extensive research on *Tert*, there has been limited investigation targeting the *Tert* gene into ES cells and generating mammalian models with germline *Tert* overexpression. Only one study employed cytosine‐adenine‐guanine (CAG) promoters to induce *Tert* overexpression in mouse oocytes (Artandi et al., 2002). However, this was reported to lead elevated cancer rates with the absence of anti‐aging effects. As a result, the effects of systemic telomerase overexpression on organismal longevity and tumor development remain contentious. In this study, we introduced the use of gene‐targeting techniques in mouse ES cells and created a novel mouse *Tert* *(mTert)* transgenic mouse model (*TertKI* mice) driven by the human elongation factor 1α (*EF1α*) promoter. To investigate the effects of the *Tert* gene on longevity and health, we conducted long‐term investigation on the lifespan of *TertKI* mice in successive generations. By utilizing this sophisticated technology, we can ensure targeted integration of the *Tert* gene into the *Rosa26* locus, minimizing integrational disruption and maximizing controlled *Tert* overexpression. Furthermore, we evaluated the recovery and response of *TertKI* mice to acute injury using skin injury and colitis models, and examined the effects of *Tert* overexpression on other health indicators, including genotoxicity, routine blood tests, and blood biochemistry in aged *TertKI* mice. Employing this sophisticated technique, our aim is to underscore not only the efficacy of *Tert* knock‐in in extending lifespan and repairing damage but also the superiority of ES cell targeting technology in achieving safe genetic modifications.

## 2 RESULTS

### 2.1 Successful generation of the *TertKI* model

**FIGURE 1:**
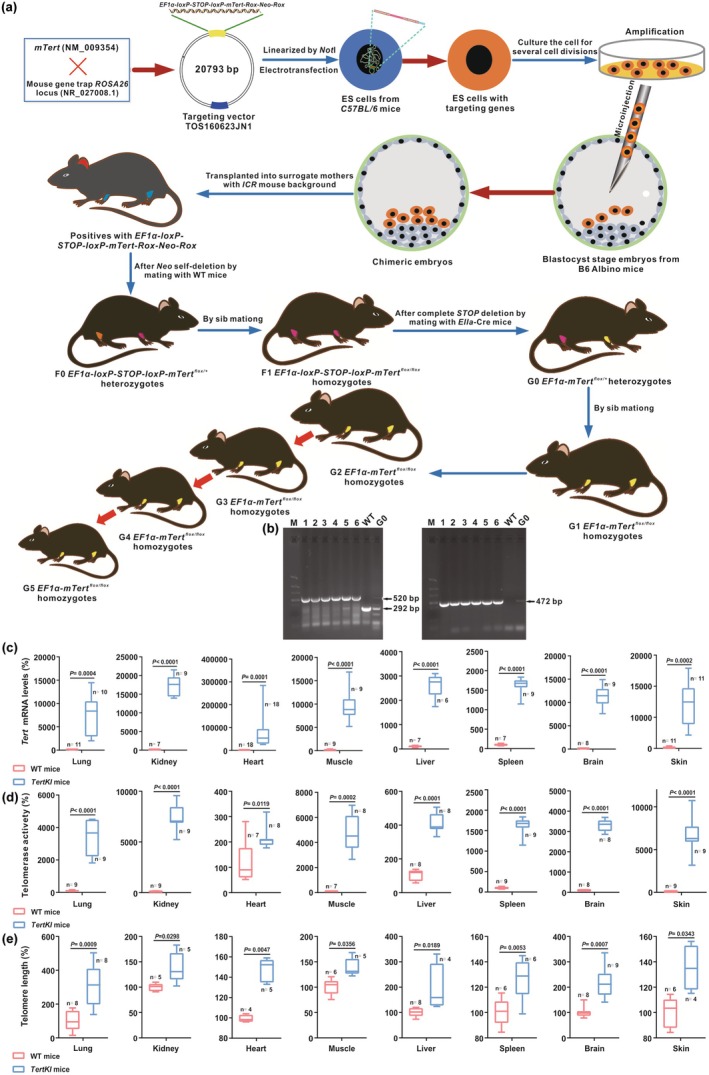
Generation, identification and phenotype of *TertKI* mice. (a) The schematic diagram illustrates the successful generation of *TertKI* mice that were stably inherited for four consecutive generations. The *EF1α‐mTert* transgene was targetedly integrated into the *Rosa26* locus via ES cells, which involved construction of a targeting vector, ES cell culture, transfection, identification and proliferation of targeted ES cells. The targeted ES cells were then used to produce chimeric mice by injection into blastocysts. Subsequent breeding of the chimeric mice resulted in the birth of offspring carrying the modified gene. The removal of the *Neo* ^*r*^ gene *3′* to the *mTert* gene led to the production of F0 heterozygous and F1 homozygous mice lacking *Neo* ^*r*^, while the deletion of the *Stop* sequence between the *EF1α* promoter and *mTert* cDNA resulted in G0 heterozygous and G1–G5 homozygous mice. (b) Genotype identification was conducted on the G5 (5th generation) *TertKI* mice using three sets of primers. *Tert*_F1 and *3′Arm*_R primers amplify 520 bp from *Neo‐Rox*‐deleted allele but 4315 bp prior to deletion. *5′Arm*_F3 and *3′Arm*_R primers amplify 292 bp fragment from the WT *Rosa26* locus only, whereas the length of DNA was 9965 bp in the original knock‐in construct and 6170 bp after the *Neo‐Rox* deletion. The primer pair of *EF1α*_F2 and *Tert*_R3 primers detect 472 bp from *lox‐STOP*‐removed allele but 1349 bp from original insert. Therefore, G5 homozygous *EF1α‐mTert* ^*flox/flox*^ mice (as shown in pups 1–6) were anticipated to display 520 bp (left) and 472 bp (right) PCR products, G0 heterozygous mice (*EF1α‐mTert* ^*flox/+*^) should exhibit three bands of 520 bp, 292 bp (left) and 472 bp (right), whereas wild‐type (WT) mice were expected to be positive for 292 bp (left) DNA only. (c–e) A comparative analysis was performed to assess *mTert* gene expression, telomerase activity, and telomere length in *TertKI* mice and WT mice. The mRNA expression level of *mTert* gene (c), telomerase activity (d), and telomere length (e) in various organs and tissues of *TertKI* and WT mice aged 6 weeks were determined. Evaluations were conducted in six‐week‐old *TertKI* mice (in blue), and WT counterparts (in red), on a variety of organs and tissues (lung, kidney, liver, heart, muscle, spleen, brain and skin derived from three germ layers. Data are presented as mean ± standard deviation (SD). Statistical differences are represented on the graph, with *p <* 0.05 indicating a significant difference and *p <* 0.01 representing a highly significant difference.

We successfully generated *TertKI* mice by precisely integrating the mouse *Tert* gene into the *Rosa26* locus of mouse ES cells through gene targeting technology. This transgene was under the control of the *EF1α* promoter, ensuring the stable inheritance and high expression of the *Tert* transgene through successive generations (Figure 1a).

To streamline the *Tert* gene targeting in *C57BL/6* ES cells, we constructed a homologous recombination cassette containing the *EF1α‐LoxP‐STOP‐LoxP‐Rox‐tACE‐Dre‐TK‐Neo‐Rox*, which facilitated rapid, precise, and straightforward transgene integration into the *Rosa26* locus. The targeting vector was designed to enable deletion of the *Neo* and *STOP* sequences through Dre and Cre recombinase activity, ensuring the correct placement of the *EF1α* promoter upstream of the *mTert* gene. Restriction digestion and gene sequencing confirmed the accurate construction of the *Tert* targeting vector (Figures S1 and S2 and Appendix S1).

Out of 94 G418‐resistant ES clones screened, 17 exhibited potential targeting based on polymerase chain reaction (PCR) analysis (Figure S3). Southern blotting was further carried out which confirmed correct targeting event in 6 of the 17 positive clones (1A4, 1B9, 1B12, 1F11, 1G11, and 1H11) (Figure S4). Ten pups from 1F11 and 1H11 ES cells, born from surrogate mice, displayed coat color chimerism. PCR screening confirmed that the genotypes of chimeric mice matched the positive ES clones, showing participation of the targeted ES cells during development (Figure S5).

The 1F11 chimeric male mice were mated with wild‐type (WT) *C57BL/6* female mice, germline transmission was verified in nine offspring (F0 mice), indicating the successful creation of a heterozygous mouse lineage with the desired genetic traits. Genotyping demonstrated the consistent inheritance of the *mTert* targeting sequence and effective self‐deletion of the *Neo* gene through Dre recombination in the F0 generation (Figure S6). DNA sequencing also verified anticipated transgene sequence in the F1 mice (Appendix S1).

Out of the 51 F1 offspring from the mating of F0 mice, 47 (92.16%) survived to weaning. PCR genotyping (Figure S7) showed that 12 were WT, 23 were heterozygous, and 12 were homozygously transgenic mice. These results align with the Mendelian ratio of 1:2:1, with 25.53% (12/47) homozygously transgenic mice, indicating that the *Tert* transgene didn't interfere with mouse development or growth, or survival.

The F1 mice carrying *EF1α‐loxP‐STOP‐loxP‐mTert* ^*flox/flox*^ genotype were bred with *EIIa*‐Cre‐expressing mice to delete the *STOP* sequence, resulting in the removal of the *STOP* and one *loxP* site. This allowed the functionally critical element *Tert* to be directly linked to its upstream constitutive promoter of *EF1α*. From breeding of two cohorts of mice *EF1α‐loxP‐STOP‐loxP‐mTert* ^*flox/flox*^ and *EIIa*‐Cre, a total of 109 offspring were obtained, and G0 *EF1α‐Tert* ^*flox/+*^ heterozygotes were identified by PCR analyses (Figure S8). Among them, 36 mice were positive for complete removal of the *STOP*, leading to the generation of G0 heterozygous mice with *EF1α‐mTert* ^*flox/+*^.

Through full‐sib mating of G0 *EF1α‐Tert* ^*flox/+*^ heterozygous mice, a total of 104 progeny were obtained. Among them, 24 mice (11 males and 13 females, 23.08%) were identified as G1 homozygotes (*EF1α‐mTert* ^*flox/flox*^) which were positive for 472 bp (floxed) and 520 bp (Roxed) PCR products, but negative for 292 bp (WT) DNA; 53 mice (28 males and 25 females, 50.96%) remained as heterozygotes (*EF1α‐mTert* ^*flox/+*^) positive for three PCR products of 472, 520, and 292 bp; and 27 mice (13 males and 14 females, 25.96%) were WT mice with the 292 bp band only (Figure S9). The likelihood of obtaining homozygous transgenic mice was approximately 23.08%, which closely matched the expected Mendelian inheritance rate of 25%, indicating successful generation of *TertKI* mice with no disadvantage. The sequencing results of G1 *TertKI* mice confirmed the findings of PCR analysis (Appendix S1). Furthermore, in subsequent generations (G2*–*G5) of *TertKI* littermate mating, *EF1α‐Tert* ^*flox/flox*^ homozygous mice were obtained with a litter size of 6*–*12, indicating stable inheritance of the transgene for at least 5 generations with no significant difference from *C57BL/6* average litter size (Figure 1b).

### 2.2 Enhanced phenotype in robust *TertKI* mice: High transcription of *Tert* gene and increased telomerase activity without notable toxicities

*TertKI* mice and WT mice exhibited similar phenotypic characteristics across various parameters. The coat color, body size, and other visible features did not reveal any significant differences between the two groups (Figure S10a). In the open‐field arena, both *TertKI* and WT mice demonstrated comparable locomotor activity and exploration patterns. Social behaviors, including sniffing, grooming, and play behavior, were similar between the two groups.

The body weights of *TertKI* and WT mice did not show any significant variations in the first 3 days after birth. However, during postnatal days 5–23, the *TertKI* mice exhibited a noticeable and rapid weight gain (*p <* 0.05) (Figure S10b), indicating a potential effect of *Tert* genetic modification on the postnatal growth and development of the mice. Furthermore, the organ‐to‐body weight ratios (coefficients) of selected organs (heart, liver, spleen, lung, kidney, brain, and testis) were not significantly different between *TertKI* and WT mice (Figure S10c), suggesting similar organ development and maintenance of mice in the two groups.

During routine autopsies of all 98 naturally deceased *TertKI* mice, we thoroughly examined organs including the colon, liver, spleen, heart, stomach, kidneys, brain, and lungs. The postmortem analyses revealed five cases of hepatomegaly and six cases of splenomegaly, but there was no obvious evidence of abnormal tissue hyperplasia and tumor growth. These findings suggest that there is no pathological change associated with organ/tissue hyperplasia or tumorigenesis in the *TertKI* mouse models (Figure S10d). Histological examination was also performed to assess the cellular and tissue morphology of selected organs and tissues, including brain, lung, liver, testis, posterior gluteal muscle, dorsal skin, spleen, heart, and kidney. Microscopic analysis of hematoxylin and eosin (H&E)‐stained tissue sections revealed no significant histological differences between *TertKI* and WT mice. The cellular architecture, tissue organization, and overall morphology of the examined organs and tissues appeared comparable in both groups. No notable abnormalities or pathological alterations were observed (Figure S10e). These findings suggest that *Tert* overexpression does not seem to promote tumor occurrence.

The *Tert* overexpression in the *TertKI* mice was firstly determined by relative *Tert* mRNA level in respective organs of *TertKI* and WT mice, via RT‐qPCR using *Tert*_F2 (*5′*‐GGATTGCCACTGGCTCCG‐*3′*) and *Tert*_R2 (*5′*‐TGCCTGACCTCCTCTTGTGAC‐*3′*) primers, which amplified 279 bp DNA from transcripts of the transgene, as well as from the endogenous splicing variants encoding 1122 AA and 729 AA. Significantly elevated levels of *Tert* mRNA were indeed detected across all organs and tissues tested, including heart, liver, spleen, lung, kidney, brain, muscle, and skin in comparison to endogenous *Tert* mRNA present in WT *C57BL/6*J mice (*p <* 0.05) (Figure 1c). The ratios of expression levels were varied across different organs, with the highest ratios observed in the heart (695×), spleen (176×), and kidney (172×). Intermediate ratios were found in the brain (112×), skin (72×), muscle (94×), and lung (75×), while the lowest expression ratio was observed in the liver (26×). These variations are likely attributable to a combination of organ‐specific regulation of the *EF1α* promoter, endogenous *Tert* transcription and/or *Tert* mRNA stability.

The telomerase activity in WT and transgenic organs was then assessed using the Telomerase Activity Quantification qPCR Assay Kit. Consistent with *Tert* mRNA overexpression, *TertKI* mice also exhibited increased telomerase activity compared to WT mice (*p <* 0.05) (Figure 1d). Telomerase activity was significantly (*p <* 0.05) elevated in the transgenic lung (36×), kidney (74×), heart (2.4×), muscle (47×), liver (4.1×), spleen (16×), brain (33×), and skin (66×), indicating enhanced telomerase activity in these organs. However, the enhanced enzyme activity in the *TertKI* mice was not entirely correlated with the quantity of mRNA overexpression, suggesting that there may be tissue‐specific regulation of *Tert* mRNA stability and/or translation. The telomerase RNA component (*Terc*) is a non‐coding RNA served as a template for telomere replication. We next measured expression of the *Terc* in WT and *TertKI* mice and found that *Tert* overexpression had no influence over the *Terc* expression, indicating that *Terc* expression might be the limiting factor of the telomerase activity in the *TertKI* mice (Figure S10f).

The length of telomere, the protective caps at chromosome ends, was measured in *TertKI* mice using the TeloTAGGG Telomere Length Assay Kit. Telomeres were extended in the lung (3.1×), kidney (1.3×), heart (1.6×), muscle (1.2×), liver (1.5×), spleen (1.3×), brain (2.2×), and skin (1.2×) of *TertKI* mice compared to WT mice (*p <* 0.05) (Figure 1e). It is worth to note that there are reduced scales from increased *Tert* mRNA expression to telomerase activity to telomere length in the *TertKI* mice, and this could be associated with tissue‐specific limits of translation capacity and telomere extension ability.

The potential teratogenic and genotoxic effects were subsequently evaluated. There was no significant difference in micronucleated polychromatic erythrocytes (Figure S11a) or in the deformation rates of sperm between WT and *TertKI* mice (Figure S11b). Moreover, *Tert* overexpression had no significant alteration in blood biochemical indices including glutamic‐pyruvic transaminase (Gpt) for liver function, glutamic‐oxaloacetic transaminase (Got1) as a prognostic marker for pancreatic ductal adenocarcinoma, or urea level for kidney function (Figure S11c). Although routine blood tests indicated that most indices in the *TertKI* mice were within the normal range, interestingly, some altered indices in *TertKI* mice showed healthier status compared to those of WT mice, and this included an increase in white blood cell count (6.08 vs. 3.47), lymphocyte count (2.9 vs. 1.53), platelet crit (0.51 vs. 0.36), hemoglobin level (115.75 vs. 92.67), mean corpuscular hemoglobin (17.68 vs. 16.11), reduced neutrophilic granulocyte ratio (40.48 vs. 45.37, ns), mean platelet volume (7.18 vs. 8.23, ns) and others (Table S1). In addition, the presence of carbohydrate antigen 72‐4 (CA72‐4), a tumor marker for gastric and colorectal cancers, was examined in the serum of 18‐month‐old (aged) and 6‐week‐old (young) of both WT and *TertKI* mice. The *TertKI* mice showed higher values of CA72‐4 in serum (1.56 ± 0.89 vs. 0.58 ± 0.27 in 6‐week mice; 0.5 ± 0.13 vs. 0.28 ± 0.13 in 18‐month mice), but they were not significantly deviated from the normal range (0–9 U/mL).

Furthermore, at 6 weeks of age, there were no significant differences in CA72‐4 levels in the stomach (60.66 ± 5.1 vs. 51.10 ± 7.91) or colon (32.25 ± 6.76 vs. 23.05 ± 5.74) of *TertKI* mice compared to WT mice. At 18 months of age, only the stomach CA72‐4 levels were slightly higher in the *TertKI* mice (49.56 ± 0.89 vs. 39.1 ± 0.89), while colon CA72‐4 levels were lower (16.65 ± 0.51 vs. 28.82 ± 7.27) than that in WT mice (Table S2).

To examine chronic disease effects, *TertKI* mice were exposed to urethane to establish a lung cancer model and compared with WT mice. Initially, no significant lesions were observed in untreated *TertKI* and WT mice (0 days). After 42 days of urethane treatment, both groups exhibited complete alveolar structure with widened alveolar septa and congested capillaries. However, bronchial epithelial cells in the *TertKI* mice showed subtle necrosis and exfoliation. By day 92, both groups had significant lesions with numerous tumor cells in alveolar walls, but nodules appeared only in WT mice (Figure S12a,b). The *TertKI* mice showed more pronounced alveolar wall widening (as a result of tumor cell proliferation). After 112 days, large areas of tumor cells were observed in the lungs of the *TertKI* mice, and the CA72‐4 levels were also increased significantly (Figure S12c). Urethane treatment led to more significant weight loss and earlier death in the *TertKI* mice compared to WT mice (Figure S12d). However, no carcinogenesis was developed in other organs (liver, kidney, colon, stomach) with urethane treatment (Figure S12e).

### 2.3 Unparalleled longevity in *TertKI* mice

**FIGURE 2:**
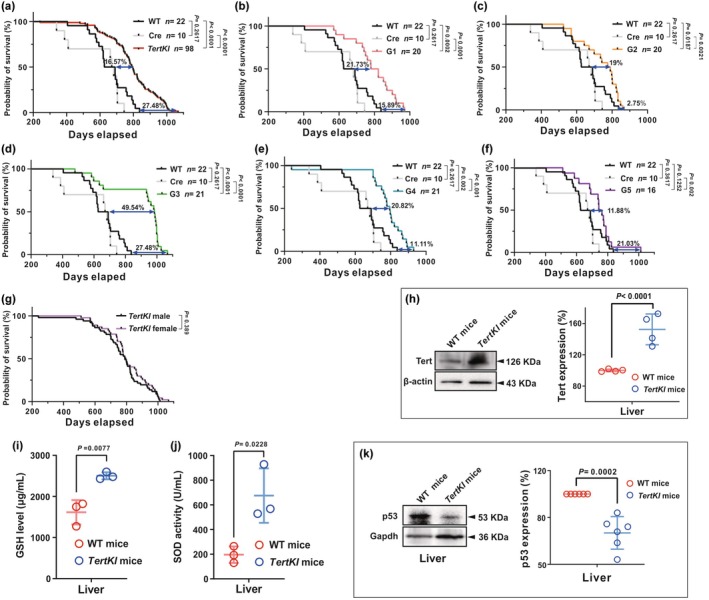
Effects of *Tert* overexpression on lifespan and antioxidant capacity in mice. (a) *Kaplan–Meier* survival curve showing prolonged lifespan of G1–G5 *TertKI* mice compared to WT and *EIIa*‐Cre mice. (b–f) Individual survival curves for each generation of *TertKI* mice (G1–G5) compared to WT and *EIIa*‐Cre mice. (g) *Kaplan–Meier* survival curve separated by gender, showing comparable lifespan of female (*n =* 47) and male (*n =* 51) *TertKI* mice. Statistical differences are represented on the graph, with *p <* 0.05 indicating a significant difference. (h) The protein expression levels of Tert in the liver were evaluated through Western blot analysis (*n* = 4). (i) Hepatic GSH levels in WT (red) and *TertKI* (blue) mice (*n* = 3). (j) SOD activity in the liver of WT (red) and *TertKI* (blue) mice (*n* = 3). (k) Western blot analysis of p53 protein expression and quantification in liver of WT (red) and *TertKI* (blue) mice (*n* = 6). Data are presented as mean ± standard deviation (SD). Statistical differences are represented on the graph, with *p <* 0.05 indicating a significant difference and *p <* 0.01 representing a highly significant difference.

Our study subsequently aimed to investigate the impact of *Tert* overexpression on the lifespan of mice. For this purpose, we compared the natural lifespan of *TertKI* and WT counterparts at G1–G5 generations, all were housed under standard conditions. The results of our investigation revealed remarkable changes in the longevity that are of significant interest. First and foremost, the maximal lifespan of the *TertKI* mice from G1 to G5 exhibited an impressive increase of 27.48% compared to the WT mice (Figure 2a), and the median lifespan of *TertKI* mice was extended by 16.57%. These findings provide compelling evidence that *Tert* overexpression positively influences the lifespan, indicating potential benefits for overall health and aging resilience. Furthermore, a more detailed analysis across the various generations (G1–G5) of *TertKI* mice highlighted a consistent trend of extended lifespans when compared to the WT counterparts (Figure 2b–f). Interestingly, G3 mice displayed notably most prolonged lifespan (Figure 2d). Moreover, it is worth noting that female *TertKI* mice had a trend of longer lifespan in comparison to the male counterparts, although not reaching statistical significance (Figure 2g). These data provide a strong support for the concept that *Tert* overexpression has a significant and consistent positive effect on the longevity of mice across multiple generations. This impact on lifespan underscores significant benefits of *Tert* gene modulation for enhancing overall health and aging outcomes.

Oxidative damage resulting from metabolic processes has been identified as a contributing factor in limiting lifespan to some extent, and its accumulation has been observed in various tissues and species as they age (Campisi et al., 2019). It is suggested that *Tert* may play a role in modulating oxidative stress and protecting cells from oxidative damage by regulating antioxidant defense systems (Sahin & Depinho, 2010; Saretzki, 2009). As *Tert* expression was notably increased in the liver of *TertKI* mice (Figure 2h), we proceeded to evaluate the levels of antioxidant molecules, namely glutathione (GSH) and superoxide dismutase (SOD), within the mouse liver tissues. The *TertKI* liver demonstrated elevated levels of GSH (2502.72 vs. 1615.15 μg/mL) and SOD (675.21 vs. 195.97 U/mL) compared to WT liver, indicating an improved antioxidant capacity in the *TertKI* mice (Figure 2i,j).

The p53 protein is involved in cellular growth regulation and tumor suppression. Previous research highlighted the significance of genomic instability, telomere dysfunction, and DNA damage in triggering cellular senescence through the p53 pathway, particularly in older individuals (Donato et al., 2015). With this in mind, we investigated the expression of p53 in the liver of *TertKI* mice using Western blotting. Intriguingly, we observed a decreased p53 expression in the liver of *TertKI* mice (Figure 2k).

### 2.4 Regenerative potential demonstrated by *TertKI* mice

The regenerative capacity of Tert in rejuvenating organisms by fending off illnesses and recuperating from injuries is well documented (Calcinotto et al., 2019; Mylonas et al., 2021). Considering the extended median lifespan observed in the *TertKI* mice, we conjecture that the *Tert* gene may bestow other advantageous traits on the mice, particularly in terms of recuperating from acute skin and intestinal injuries, and the outcomes are indeed promising.

**FIGURE 3:**
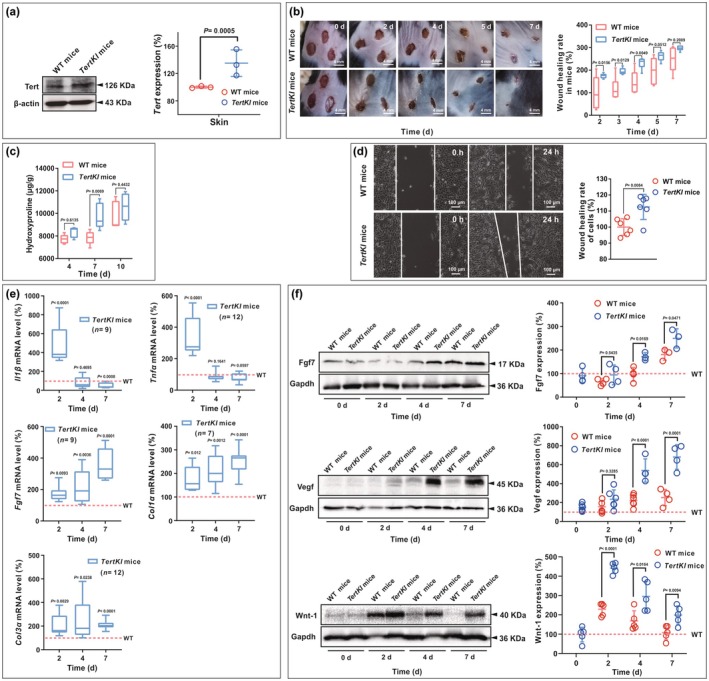
Effects of *Tert* overexpression on skin wound healing and the expression of inflammatory and growth factors. (a) The protein expression levels of Tert in the skin of both WT (C57BL/6) and *TertKI* (G3) mice (over 8 weeks old) were assessed using Western blot analysis. (b) The wound healing rate in acute skin wounds of WT and *TertKI* mice at 0, 2, 4, 5 and 7 days (*n* = 6). (c) Hydroxyproline levels surrounding the wounds in WT and *TertKI* mice (*n* = 5). (d) In vitro measurement of fibroblast wound healing ability from WT and *TertKI* mice (*n* = 6). Fibroblast cells were harvested from both WT and Tert‐overexpressing (*TertKI*) mice, then cultured under controlled conditions. An artificial wound was created within the fibroblast cell layer to simulate a real wound scenario. Subsequently, images were taken at designated time points (0 and 24 h) to monitor the response of fibroblast cells from both groups to the artificial wound. (e) The mRNA expression levels of inflammatory factors (*Il1β* and *Tnfα*) and growth factors (*Fgf1*, *Col1α1* and *Col3α1*) around the wound of mouse skin were measured using RT‐qPCR. The expression of each gene in *TertKI* mice was calculated relative to its expression in WT mice on the same day. The gene expression in WT mice was normalized to 100% every day and is represented by the red dotted line in this panel. (f) The protein expression levels of Fgf7, Vegf, and Wnt‐1 were assessed using western blot from three independent experiments. The skin samples were collected from mice different from those used to measure wound healing. Thus, we ensured that the collected tissue samples did not interfere with the wound healing measurements, and we strictly distinguished the mice used for RNA and protein extraction from those used for wound healing assessments. Protein expression quantification of each band was calculated relative to the sample of WT mice on day 0. The protein expression in WT mice on day 0 was normalized to 100% and is shown as the red dotted line in this panel. Data are presented as mean ± standard deviation (SD). Statistical differences are represented on the graph, with *p <* 0.05 indicating a significant difference and *p <* 0.01 representing a highly significant difference.

In comparison to WT mice, *TertKI* mice displayed increased Tert expression in skin (Figure 3a), faster skin wound healing (Figure 3b), and improved hair growth, which were similar to a previous report (Jaijyan et al., 2022). Histological analysis of the wounds revealed reduced inflammatory cell infiltration and faster wound repair with a flat new epidermis in the *TertKI* mice (Figure S13). Additionally, normal skin features such as hair follicles, sebaceous glands, and neovascularization appeared earlier in *TertKI* mice than in WT mice (Figure S13). Furthermore, collagen fiber remodeling was improved in *TertKI* mice, with thick collagen fibers interwoven into a network (Figure S14). The main component of collagen protein, hydroxyproline, was significantly enhanced at 7 days of wound healing in *TertKI* mice, indicating improved recovery from skin injury (Figure 3c).

Moreover, we demonstrated that *Tert* overexpression enhanced the migration ability of mouse skin fibroblasts in vitro (Figure 3d). Neonatal mouse fibroblasts with high vitality were obtained and confirmed through Vimentin fluorescence staining (Figure S15). Scratch assay results showed that the *TertKI* skin fibroblasts exhibited greater migration ability compared to those from WT mice (Figure 3d).

Furthermore, we assessed the expression of inflammatory factors, such as mouse interleukin 1 beta (Il1β), tumor necrosis factor alpha (Tnfα), and growth factors (Wnt‐1, Vegf, and Fgf7), as well as Col1α and Col3α in *TertKI* mice during wound healing. Compared with WT mice, *TertKI* mice had significantly higher mRNA levels of *Il1β* and *Tnfα* on day 2, followed by a rapid decline and lower levels than WT mice on day 7 (Figure 3e). This indicates that *TertKI* mice mount a quick inflammatory response to combat injury, followed by a prompt resolution of inflammation to prevent potential adverse effects during wound healing. Furthermore, the gene expression of growth factors, including mouse fibroblast growth factor (*Fgf7*) and collagen (*Col1α1* and *Col3α1*), was consistently upregulated in *TertKI* mice, supporting their enhanced wound healing capacity.

At the protein level, expression of growth‐related factors, such as Fgf, Vegf, and Wnt‐1, were significantly increased during the course of wound healing, and the *TertKI* mice showed higher levels of the Wnt‐1/Vegf protein, further confirmed the robust skin regeneration in *TertKI* mice (Figure 3f). These findings suggest that *Tert*‐mediated upregulation of related factors and fibroblast activity contribute to the enhanced skin wound healing observed in *TertKI* mice.

**FIGURE 4:**
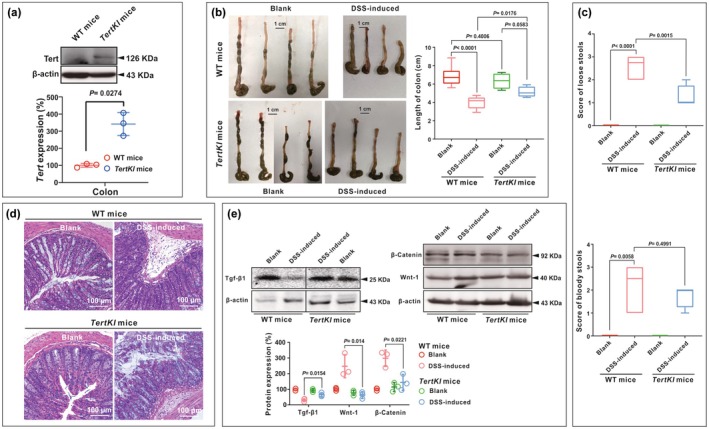
Effects of *Tert* overexpression on DSS‐induced colitis. (a) The protein expression levels of Tert in the colon of both WT and *TertKI* mice were assessed using Western blot analysis. (b–e) The WT (C57BL/6) and *TertKI* (G5) mice (both over 8 weeks old) were subjected to treatment with either regular drinking water or water containing 3% DSS for a duration of 7 days to induce colitis. The mice were divided into four groups: WT‐Blank (*n* = 8), WT DSS‐induced (*n* = 8), *TertKI*‐Blank (*n* = 4), and *TertKI* DSS‐induced (*n* = 4). Various colitis‐related parameters were assessed and analyzed, including the colon length (b), loose stools score, bloody stool score (c), colon pathology (d), and western blot was conducted three times to assess the protein expression levels of Tgf‐β1, Wnt‐1 and β‐Catenin (e). Data are presented as mean ± standard deviation (SD). Statistical differences are represented on the graph, with *p <* 0.05 indicating a significant difference and *p <* 0.01 representing a highly significant difference.

Administration of dextran sodium sulfate (DSS) is commonly used to induce colon injury and monitor colon repair in mice (Okayasu et al., 1990). Here, we induced acute colitis with 3% DSS in drinking water for seven consecutive days and studied mucosal resistance to acute injury in *TertKI* mice, which showed increased Tert expression in the colon compared to those in WT mice (Figure 4a). The stool characteristics, colon length, and other relevant parameters were compared between the *TertKI* and WT mice, to evaluate the impact of *TertKI* on the response to DSS‐induced colon injury. The length of the colon, an indicator of the severity of colon injury and colonic inflammation, showed no significant difference between non‐induced *TertKI* (6.33 ± 0.85) and WT mice (6.86 ± 1.04, *p =* 0.4006) (Figure 4b). Following 7 days of the colitis induction, WT mice exhibited a reduction in colon length (from 6.86 ± 1.04 to 4.02 ± 0.66). However, *TertKI* mice demonstrated tolerance to colon shortening (from 6.33 ± 0.85 to 5.13 ± 0.58), with no significant difference to the *TertKI* control group (*p =* 0.0583) (Figure 4b).

During the period of self‐recovery from injury, the mice were monitored daily for changes in stool consistency and the presence of blood in the stool as indicators of colon injury and inflammation. *Tert*‐overexpression was also shown to benefit relieving loose stools (Figure 4c, *p =* 0.0015), but not the reduction of bloody stools which was statistically insignificant (Figure 4c, *p =* 0.4991). Histological analysis was carried out to evaluate the degree of colon injury, inflammation, and repair. Histopathological analysis of the colon revealed tissue injury induced by DSS in WT mice, including mucosal epithelial cell necrosis, submucosal edema, severe inflammatory cell infiltration, and small vessel dilation. However, these pathological features were alleviated in the *TertKI* mice (Figure 4d).

Transforming growth factor beta (Tgf‐β1), a multifunctional cytokine, plays a crucial role in promoting mucosal wound healing in inflammatory bowel disease (Martin‐Rodriguez et al., 2021). During the initial stages of wound healing, the Wnt/β‐Catenin signaling pathway was activated, leading to the upregulation of several target genes involved in cell proliferation, migration, and tissue repair (Nusse & Clevers, 2017). Western blot analysis was then performed to measure changes in the expression levels of Tgf‐β1, Wnt‐1, and β‐Catenin in colon tissue to investigate the impact of *TertKI* on colon injury repair. In WT mice, Tgf‐β1 expression was reduced and Wnt/β‐Catenin were elevated after DSS induction. However, the *TertKI* mice exhibited resistance to Tgf‐β1 reduction and inhibition of the Wnt/β‐Catenin signaling pathway induced by DSS (Figure 4e). Collectively, these findings suggest that *Tert* overexpression protects mice from DSS‐induced colitis.

## 3 DISCUSSION

The TERT plays an important role in maintaining telomere length and offers a therapeutic potential and a drug target for aging–related diseases. In this study, we targeted the *EF1α‐Tert* transgene into the *ROSA26* locus with a Cre‐LoxP‐inducible system. *Tert* overexpression was shown to extend mouse lifespan with no apparent adverse effects. The *TertKI* mice also displayed accelerated recovery from skin wound healing and DSS‐induced colitis.

### 3.1 Successful generation of *TertKI* mice marks a significant step forward for research on anti‐aging

In the realm of gene‐based therapy, the modulation of the genome through viral or non‐viral vectors, as well as direct gene editing techniques, has been a subject of exploration (Brody, 2018). In this basic research study, we harnessed *ROSA26*‐based gene targeting technology in ES cells, to minimize potential integrational effects on host genome or on transgene expression driven by a chosen promoter (Carofino & Justice, 2015; Friedel & Soriano, 2010). A strong promoter is essential for functional evaluation, and human *EF1α* was chosen in this project as it displayed ubiquitous transcriptional activity in both mouse ES and differentiated tissues (Wang et al., 2008), while viral and non‐eukaryotic promoters might have the risk of epigenetic modification in mammals. We introduced the *loxP‐STOP‐loxP* sequence between the *EF1α* promoter and the *Tert* transgene, and this was to ensure generation of transgenic mice with an option for tissue‐specific Tert overexpression, if ubiquitous Tert overexpression were lethal (Lampreht Tratar et al., 2018). The current study demonstrates that *Tert* overexpression exhibits no major adverse effects to survival or fertility, and the *TertKI* mice shall facilitate an array of research opportunity for Tert function.

### 3.2 Balancing practicality and precision in telomere length measurement

We acknowledge the importance of measuring absolute telomere length for understanding the physiological impact of telomere shortening, as it allows for more precise determinations of whether telomere length has reached critical thresholds that could affect chromosomal stability and cellular function (Aubert & Lansdorp, 2008). However, for telomere length comparison at the individual level in WT and TertKI mice, we believe the relative measurement is suitable for identifying initial trends in telomere length differences. It is because that the telomere length of different chromosomes is very different, and this difference has individual conservation. Therefore, even absolute telomere length detection at the cellular level is difficult to elucidate molecular differences in telomere length (Karimian et al., 2024). Moreover, the organs and tissues we selected—including the lung, kidney, heart, muscle, liver, spleen, brain, and skin—contain various cell types. And given that we used 6‐week‐old mice, which are relatively young and less likely to exhibit critically short telomeres or associated molecular abnormalities, this relative measurement is appropriate. For future studies, particularly those investigating the biological effects and molecular changes, we plan to incorporate absolute telomere length measurements to gain deeper insights into telomere dynamics.

### 3.3 *Tert* overexpression displays no special toxicities in *TertKI* mice

Alongside effectiveness, the safety of gene therapy has been a prevailing concern since its inception (Tang & Xu, 2020), and research is necessary to assess the feasibility, safety, and potential benefits of *Tert* gene editing as a therapeutic avenue for age‐related conditions. The debate surrounding *Tert* gene therapy for rejuvenation and life extension centers on whether Tert is the “natural ally” of aging or the “molecular instigator” of cancer (Shay, 2016; Shay & Wright, 2019). This debate arises from the fact that telomerase activation is observed in a significant majority of human cancers. Additionally, mutations in the *TERT* promoter have been identified as one of the mechanisms leading to the activation of *TERT* transcription (Hafezi & Perez Bercoff, 2020). Despite no sign of tumor formation in the *TertKI* mice, we measured levels of CA72‐4, a biomarker for gastrointestinal cancers, particularly gastric and pancreatic cancers, and found no significant differences between the transgenic and WT animals. However, when exposed to urethane, *TertKI* mice did appear to develop lung cancer more rapidly than WT mice. This suggests that *Tert* overexpression can increase the likelihood of carcinogenesis under chronic harmful stimulation, and a precaution shall be taken in designing gene therapy, as human living environment are different from experimental settings.

We also observed 9.86% of pre‐weaning loss, including 2/11 in chimeric mice, 1/9 in F0 mice, and 4/51 in F1 *EF1α‐Tert* homozygous mice, and this aligned with the previously reported range of pre‐weaning mortality rate in the *C57BL/6* strain, which can vary from less than 10% to as high as 49% (Morello et al., 2020). Comprehensive genotoxicity assessments are essential for a thorough evaluation of potential risks linked to genetically modified organisms (Sabatino et al., 2022). We examined mice that died of pre‐weaning mortality and did not detect any tumor, or particular abnormalities. For mice that died of natural aging at the end‐of‐life spectrum, we carried out anatomical and histological examination of various organs and observed no significant abnormalities, or signs of tumor development, or genotoxicity, or abnormal blood biochemical indices in the transgenic mice. This is consistent with the majority of previous reports that *Tert* transgene alone does not induce genotoxic effects, but offers an advantage over viral protein‐mediated immortalization methods that may inactivate tumor suppressor genes (Kogan et al., 2006). *Tert* itself does not act as a dominant oncogene (Morales et al., 1999), or pose significant risk to genome integrity within our experimental design, and this further strengthens the safety of *Tert* transgenic models. Consistent with our discovery, an induced Tert expression via a novel compound was shown to stimulate adult neurogenesis and to preserve cognitive function without evident cancer risk (Shim et al., 2024).

### 3.4 *TertKI* mice, a showcase with extraordinary lifespan extension

The endogenous mouse *Tert* is known to express at extremely low levels (0*–*2.682 RPKM) across different tissues, whereas the human *EF1α* promoter we used to drive the *mTert* transgene in this study exhibits high expression (713*–*3498 RPKM) in various human adult tissues (https://www.ncbi.nlm.nih.gov/gene/1915/?report=expression). Our study unveils enhanced telomerase activity and lengthened telomere in various organs of the *TertKI* mice, despite they were not in proportion to *Tert* mRNA levels. Mice generally have longer telomeres than humans (Weng & Hodes, 2000), and the increased telomere length observed in the *TertKI* mice can be attributed to several factors. Overexpression of *Tert* mRNA increases telomerase and activity, which promotes telomere elongation. Tissue‐specific regulation of telomerase activity and telomere length can lead to more pronounced elongation in certain tissues. Compensatory mechanisms and individual biological variability among the mice also contribute to the observed extension in telomere length. However, there are also limiting factors in the cells which restrict the Tert activity, and the Terc appears to be one of them.

Our study demonstrates compelling evidence that *Tert* overexpression has beneficial impact on lifespan and overall health in mice. *Tert* overexpression led to an impressive 16.57% increase in median lifespan and a remarkable 27.48% increase in maximal lifespan across five consecutive generations, signifying the stable and heritable nature of its effect. Distinct survival patterns among generations and genders suggest variations in *Tert*'s outcome. However, our control group (C57BL/6) had a slightly shorter lifespan (about 1.8 years) than typical B6 mice (Ackert‐Bicknell et al., 2015), and environmental conditions, diet, and husbandry practices can all significantly affect the lifespan of laboratory mice, with even minor variations leading to differences in longevity across studies. Despite these variations, the extension of lifespan in *TertKI* mice is a notable finding, demonstrating a clear increase compared to the control group under the same conditions. This indicates that Tert overexpression has a significant positive effect on longevity, regardless of the control group's baseline lifespan. Additionally, *TertKI* mice displayed improved antioxidant functions that likely contribute to their extended lifespan. The downregulation of p53 expression in the liver hints its involvement in the mechanisms behind the observed lifespan expansion. These findings provide valuable insights into how Tert overexpression influences longevity and the intricate interplay between antioxidant capacity and lifespan.

The *TertKI* mice continuously overexpress Tert from development under the constitutive *EF1α* promoter, aiding telomere maintenance and genome stability, which benefits physiology and lifespan. However, inducing *Tert* expression in older mice might have different effects and this was beyond the remit of the current study. Future studies should explore the effects of controlled Tert activation in aged mice to understand its therapeutic potential and associated risks in aging and age‐related diseases. Interestingly, induced TERT expression in adult mice via telomerase‐activating compound (TAC) were able to act in a similar fashion to constitutive Tert transgene (Shim et al., 2024). These findings together contribute to our understanding of the impact of *Tert* gene modulation on cellular processes and provide insights into potential anti‐aging mechanisms.

### 3.5 Increased anti‐oxidative enzymes and organ coefficient in *TertKI* animals

We observed a potential relationship between increased antioxidant enzymes (SOD and GSH) in transgenic animals and higher organ coefficients, particularly in the liver. While we suggested that the elevated levels of SOD and GSH may indicate an increase in tolerance against oxidative stress in the transgenic animals, it is essential to consider alternative explanations, such as the possibility that the oxidative stress levels are indeed higher in transgenic animals, leading to the increase in these enzymes. Future studies may include detailed analyses to assess the correlation between organ coefficient and oxidative stress markers in transgenic animals, as well as exploring the underlying mechanisms driving changes in anti‐oxidative enzyme levels. Studies involving genetic instability may also be conducted to evaluate the potential correlation. By integrating these analyses, we can gain a better understanding of the interplay between organ size, oxidative stress, genome instability, and anti‐oxidative defense mechanisms in transgenic animals, providing valuable insights into the physiological implications of *Tert* overexpression.

### 3.6 *TertKI* mice demonstrate unmatched tissue repair capacity and unleash unprecedented regenerative potential

*TertKI* mice exhibited remarkable regenerative potential beyond their extended lifespan. Accelerated skin wound healing, improved histology, and enhanced collagen remodeling suggest enhanced tissue repair in *TertKI* mice. Increased skin fibroblast migration indicates augmented regenerative capacity. These findings highlight the crucial role of *Tert* in promoting tissue regeneration, making it a potential therapy for wound healing and tissue repair. Moreover, *Tert* overexpression can prevent acute injuries and enhance resistance to DSS‐induced colitis, a disease that causes mucosal damage. We demonstrate the dual role of *Tert* in recovery and injury resistance by differential regulation of Wnt‐1 in skin wound repair and colitis. This unique aspect suggests that increased resilience to harmful stimuli contributes to overall health and rejuvenation.

Our research data also suggest that *Tert* transgenic animals display less colon deformation, functional disruption, and reduced molecular markers of injury compared to WT animals. However, the mechanism underlying these observations is not fully elucidated currently. To address this ambiguity, we shall conduct further analyses to determine whether *Tert* overexpression protects against the effects of DSS‐induced injury or promotes healing processes. This will involve exploring potential mechanisms such as modulation of inflammatory responses, enhancement of tissue repair mechanisms, or preservation of epithelial barrier integrity in *TertKI* animals. These follow‐up research shall provide a more comprehensive understanding of how *Tert* overexpression influences the response to DSS‐induced colitis.

### 3.7 Limitations

The telomerase reverse transcriptase plays a crucial role in telomere maintenance and cellular aging. Although this transgenic approach demonstrates the promise of anti‐aging gene therapy with *TertKI* models, challenges remain such as low homologous recombination rates and difficulties to introduce homologous recombination vectors without specific selection, and this can now significantly be solved in combination with CRISPR/Cas9 gene editing, with gRNA targeting specific sequence between two homologous recombination arms. The tradition knock‐in method is also time‐intensive procedures, labor intensiveness and high costs. Moreover, this is primarily limited to mouse models. The TurboKnockout® technology, developed by Cyagen, uses ES cell‐mediated techniques for precise genetic modifications, and offers advantages over CRISPR/Cas9 such as creating complex gene knockouts, large‐fragment knock‐in, and humanization models with high accuracy and no off‐target effects, ensuring 100% germline transmission, reducing production time by eliminating multiple breeding generations, and effectively enabling large genomic modifications up to 300 kb, which is useful for complex gene modeling and drug development research (https://www.labroots.com/trending/health‐and‐medicine/21708/turboknockout‐gene‐targeting‐technology‐2). Additionally, the current study focused on *Tert* overexpression in a specific genetic background of *C57BL/6*, which may limit the generalizability of the findings. Exploring *Tert* overexpression in diverse mouse strains or other animal models is essential, as aging is a multifaceted process influenced by various genetic and environmental factors.

We recognize the importance of conducting extensive research and ongoing monitoring to fully understand the potential long‐term effects and genotoxic risks associated with *Tert* transgenes and genetic modifications. While the extended lifespan and enhanced tissue repair capabilities observed in *TertKI* mice offer valuable insights, extrapolating these findings directly to human aging may require consideration of potential variations. Our study primarily provides valuable genetic insights into the effects of *Tert* on the aging process, which can be contextualized within the realm of human aging and age‐related diseases. This study represents an initial step in exploring the safety of Tert overexpression, and further research is necessary to confirm our findings and address any remaining questions.

Drug exploration on regulation of human TERT expression has made a remarkable progress recently. A TAC was identified to upregulate human *TERT* transcription, promote telomere synthesis, reduce aging hallmarks, and increase DNMT3B‐mediated promoter hypermethylation, via high‐throughput screening using transgenic cells carrying a 160‐kb human *TERT*‐Luc reporter gene. Excitingly, intraperitoneal delivery of the TAC was shown to activate TERT expression in multiple organs, to stimulate adult neurogenesis, and to preserve cognitive function without evident cancer risk (Shim et al., 2024). A combinatorial anti‐aging therapy is likely to emerge shortly for aging and aging‐related diseases.

## 4 METHODS

### 4.1 Animals and animal care

The project was approved by the Ethical Review Committee for Animal Experiment at Nanjing University of Chinese Medicine, China (permit No. ACU170304). Animal care and experimental procedures were conducted in accordance with the guidelines for the care and use of laboratory animals. Mice were housed in oblique opening cages (290 × 178 × 160 mm) under standard laboratory conditions, including a 12‐h light/dark cycle, non‐pathogenic conditions, and ad libitum access to food and water. The indoor temperature was maintained at 22°C (range 18–24°C) with a humidity of 50% (range 40%–70%).

All surgical procedures were performed under general anesthesia. Humane endpoints and euthanasia were implemented to minimize pain or suffering in accordance with the guidelines proposed by the American Veterinary Medical Association. Mice meeting the endpoint criteria with chronic pain or suffering that could not be alleviated or controlled by medication were promptly terminated using isoflurane anesthesia followed by cervical dislocation. Newborn pups without any health issues were maintained under appropriate care until the end of their lives.

Mouse breeding was following standard procedures (Fang et al., 2021). All animals used in this study were on the *C57BL/6* genetic background. Two female mice (7–8 weeks of age) were paired with a three‐month‐old male mouse and checked for the presence of a vaginal plug every morning to confirm successful mating. Each male mouse underwent a maximum of three breeding cycles.

### 4.2 Southern blot analysis

Genomic DNA was extracted from tissue samples using a commercial kit and digested with specified restriction enzymes. Digested DNA samples were separated on an agarose gel and transferred to a nylon membrane. A labeled *Neo* cDNA probe was hybridized to the immobilized DNA. The membrane was washed with high‐stringency buffer to remove unbound probe. The hybridized bands were visualized using autoradiography or chemiluminescence detection methods.

### 4.3 Tail biopsies for genotype validation and PCR conditions

To validate the genotypes of the mice, tail biopsies were collected at 3 weeks of age for genotyping analysis. Care was taken to ensure that only a minimal amount of tail tissue was collected. DNA extraction from the tail samples was performed using the TIANamp Genomic DNA Kit (TIANGEN BIOTECH, Beijing, China), following the manufacturer's instructions. Genotyping was carried out by PCR and PCR products were resolved on agarose gel electrophoresis. The specific primers targeting the regions of interest used for mouse genotyping were listed in Table S3. The PCR amplification reactions were conducted as follows: initial denaturation step at 94°C for 3 min, followed by 38 cycles of denaturation at 94°C for 30 s, annealing at 60°C for 35 s, extension at 72°C for 30 s per 1000 base pairs (1 Kbp), depending on the primer set used. The final extension was carried out at 72°C for 5 min. The genotyping results were confirmed by comparing the band patterns of the PCR products with the expected sizes for each genotype.

### 4.4 Anatomical, histological analysis and determination of organ coefficients

For the determination of organ coefficients, the weights of specific organs, such as the heart, spleen, brain, liver, lung, kidney and muscle, were weighed and recorded. The organ coefficients were calculated by dividing the organ weight by the body weight of the respective mouse, expressed as milligrams per gram (mg/g).

In addition to the standard autopsies conducted on all deceased mice, we conducted detailed examinations specifically on the *TertKI* mice. This comprehensive approach enabled us to thoroughly assess the presence of abnormal tissue growth, offering valuable insights into the long‐term effects of *Tert* overexpression. The examination encompassed all 98 dissected *TertKI* mice. Throughout the procedure, we meticulously isolated the major organs and carefully examined them to identify any tumor lesions. Histological analysis and determination of organ coefficients were performed to assess the morphological, size and structural changes in the organs of *TertKI* mice in comparison to non‐transgenic mice. A portion of the selected organs and tissues, plus posterior gluteal muscle, dorsal skin, stomach and small intestine were rapidly frozen in liquid nitrogen and stored at −80°C for subsequent analysis of *Tert* transcription levels, telomerase activities and telomere lengths. Another portion of the organs and tissues were fixed in 4% paraformaldehyde (Biosharp Life Sciences, Beijing, China, BL539A) and processed for histological examination. The fixed tissue samples were dehydrated, permeated, embedded in paraffin, and cut into 4 μm thick sections. These sections were then stained with H&E to visualize and assess the cellular and tissue morphology. The DHMI3000B light microscope from Leica Camera AG (Wetzlar, Germany) was used to compare the morphological and structural characteristics of the tissues, with two sections analyzed per sample. This analysis helps identify any histological changes associated with *Tert* expression. Additionally, in wound healing experiments, both H&E staining and Masson's trichrome staining were utilized to evaluate physiological and pathological characteristics related to tissue repair.

### 4.5 Western blot analysis

Mouse tissue or organ samples were isolated based on specific experimental requirements. Proteins were extracted using lysis buffer (Beyotime Biotechnology, Shanghai, China) containing protease inhibitor (Beyotime Biotechnology), and protein concentrations were determined with Pierce™ BCA protein assay Kit (Thermo Fisher Scientific). Equal amounts of protein were separated by SDS‐PAGE and transferred to a PVDF membrane (BIO‐RAD) using a constant current of 250 mA for 60–120 min during electrophoresis. The membranes were subsequently blocked with a 3% BSA (Biofroxx, Guangzhou, China) solution at room temperature for 1 h. The PVDF membrane was incubated with specific primary antibodies against the target proteins, including Gapdh (Affinity Biosciences, Cat. No. T0004), Tert (Abcam, Cat. No. ab32020), p53 (Affinity Biosciences, Cat. No. BF8013), Fgf7, Vegf, Wnt‐1, and β‐Catenin (SAB Signalway Antibody, Cat. No. 31162, 41,552, 35,481 and 47,993), β‐Actin (ABclonal Technology, Wuhan, China, #AC026) and Tgf‐β1 (Cell Signaling Technology, USA, #3711). The antibodies used in the experiments exhibited reactivity towards both mouse and human proteins. The membranes, after being washed with TBST, were incubated with an HRP‐labeled secondary antibody (Affinity Biosciences, #S0002) for an additional 2 h at room temperature. Chemiluminescence was performed using Clarity™ Western ECL Substrate (BIO‐RAD, #KF8005) and the signal was detected using the ChemiDoc XRS+ System (BIO‐RAD) and analyzed using Image Lab 3.0 software (BIO‐RAD).

### 4.6 Quantitative real‐time PCR (RT‐qPCR)

Total RNA was extracted from samples using Trizol Reagent (Thermo Fisher Scientific, Waltham, MA, USA, #15596026CN), and cDNA was synthesized using the iScript cDNA Synthesis Kit (BIO‐RAD, Hercules, California, USA, #1708890). RT‐qPCR reactions were performed using the CFX96 system (BIO‐RAD) and iTaq™ Universal SYBR® Green Supermix (BIO‐RAD). The relative gene expression levels were determined using the $2^{-\Delta \Delta C_{t}}$ method, with 18S ribosomal RNA as the reference gene. The sequences of primers and probes used in this study can be found in Table S4.

### 4.7 Generation of *Tert* knock‐in mice

The design strategy for generating *TertKI* mice involved the utilization of TurboKnockout® technology (Cyagen Company, China) to introduce the *Tert* gene, encoding telomerase reverse transcriptase (Tert), into the mouse genome via gene targeting in ES cells. The *Tert* gene was driven by the *EF1α* promoter and specifically inserted at the *Rosa26* site via homologous recombination in ES cells, ensuring controlled and efficient expression of *Tert*, facilitating stable transmission of the *TertKI* transgene and phenotypic characterization across subsequent generations (Figure S1).

First, the full‐length cDNA of *mTert* (NM_009354) was amplified from a bacterial artificial chromosome (BAC) clone (Invitrogen, USA) using high‐fidelity Taq polymerase. The amplified *mTert* fragments were assembled into a mouse gene trap *ROSA26* vector (NR_027008.1) harboring a cassette (Cyagen Company, China) using a specific recombination technique. Then the targeting vector was prepared, which contains the *EF1α‐loxP‐STOP‐loxP‐mTert‐PA‐Rox‐tACE‐iDreV‐PA‐TK‐Neo‐PA‐Rox*. This arrangement allows for precise and controlled genetic modifications using the Dre‐Rox recombination system (Sauer & McDermott, 2004) and the Cre‐*loxP* recombination system (Lakso et al., 1996), respectively. In this vector, *Rox* sequences were incorporated at the ends of the target gene *tACE‐iDreV‐PA‐TK‐Neo‐PA*, so that it can be Roxed out by DreV in the absence of G418 selection. While *loxP* sequences were incorporated at the ends of the target gene *STOP*, enabling precise and controlled gene manipulation after crossing with Cre transgenic mice. The resulting construct contained the *Tert* gene under the control of the *EF1α* promoter but lack other un‐wanted sequence in transgenic mice. The final targeting vector was verified through restriction enzyme digestion, Southern blot analysis by Cyagen Company, and sequencing by Tsingke Biotechnology Co., Ltd. (Beijing, China).

The *Not*I‐linearized targeting vector was electroporated into super competent *C57BL/6* mouse ES cells (Tessarollo et al., 2009), followed by G418 selection (200 μg/mL) and isolation of drug‐resistant clones. Targeted ES clones were screened using PCR in three regions (Figure S3), with specific primer sequences listed in Table S3. Positive clones showing PCR products of the expected lengths in region 1 (4858 bp, with forward primer of *Neo*_F1 derived from the end of the *Neo* gene, *5′*‐GCTGACCGCTTCCTCGTGCTTTA‐*3′* and reverse primer of *3′Arm3′*_R1, *5′*‐AAGACACCAGTTTCAGCCCAAGTTC‐*3′* which is 258 bp downstream of *3′Arm*), indicated homologous integration events, as well as the presence of other transgene fragments in region 2 (5423 bp, with forward primer of *EF1α*_F1, *5′*‐GGATCTTGGTTCATTCTCAAGCC‐*3′* and reverse primer of *tACE*_R1, *5′*‐GGACCCTGAGAGAAAGACATACCCAT‐*3′*), and region 3 (579 bp, with forward primer of *5′Arm*_F1, *5′*‐CAAAGCTGAAAGCTAAGTCTGCAG‐*3′* and reverse primer of *EF1α*_R1, *5′*‐CATAACCCGTAAAGAGGCCAGGC‐*3′*). Up to six PCR‐positive clones were expanded and further confirmed by Southern blot analysis (Figure S4), using specific probes (468 bp) and primer sequences (*Neo*_F2: *5′*‐AAGGCGATAGAAGGCGATGC‐*3′* and *Neo*_R, *5′*‐TCATCTCACCTTGCTCCTGC‐*3′*) listed in Table S3.

The selected *TertKI* ES cell clones were injected into blastocysts derived from *B6(Cg)‐Tyrc‐2 J/J* mice. These blastocysts, along with the injected ES cells, were then transferred into the uteri of pseudopregnant female of *ICR* mice. The resulting pups were examined for coat color chimerism, and their tail biopsies were genotyped by PCR analysis in region 1 (446 bp, with forward primer of *5′Arm*_F2, *5′*‐GGTGCTTGCCTTTATGCCTTTA‐*3′* and reverse primer of *EF1α*_R2, *5′*‐ACCACACACGGCACTTACCTGT‐*3′*), 2 (453 bp, with forward primer of *STOP*_F, *5′*‐GTTCCGGATCCACTACACCA‐*3′* and reverse primer of *Tert*_R1, *5′*‐CAACAGTAGCATCCATGCACC‐*3′*), and 3 (563 bp, with forward primer of *Tert*_F1, *5′*‐AAGCTCCCAGAGGCGACAATG‐*3′* and reverse primer of *tACE*_R2, *5′*‐GGCTGGTAAGGGATATTTGCCTG‐*3′*) to confirm the presence of ES‐derived cells in chimeric tail (Figure S5).

After the successful generation of chimeric mice, male mice carrying the gene‐targeted allele were crossed with WT *C57BL/6* female mice to facilitate the automatic removal of the *Neo* cassette. This breeding strategy resulted in the production of F0 heterozygous mice carrying the *EF1α‐loxP‐STOP‐loxP‐mTert* ^*flox/+*^ genotype. Genotyping analysis of tail biopsies from the offspring lacking the *Neo* cassette, was performed using PCR in region 1 (446 bp, using forward primer of *5′Arm*_F2, *5′*‐GGTGCTTGCCTTTATGCCTTTA‐*3′* and reverse primer of *EF1α*_R2, *5′*‐ACCACACACGGCACTTACCTGT‐*3′*), region 2 (453 bp, using forward primer of *STOP*_F, *5′*‐GTTCCGGATCCACTACACCA‐*3′* and reverse primer of *Tert*_R1, *5′*‐CAACAGTAGCATCCATGCACC‐*3′*), and region 3 (520 bp after *Neo‐Rox* deletion instead of 4315 bp, using forward primer of *Tert*_F1, *5′*‐AAGCTCCCAGAGGCGACAATG‐*3′* derived from the end of *Tert*, and reverse primer of *3′Arm*_R, *5′*‐AAGACCCAACCAACAGCAGAGA‐*3′* derived from the *5′* end of the *3′Arm*) to identify the F0 heterozygous transgenic mice lacking the *tACE‐iDreV‐PA‐TK‐Neo‐PA* (Figure S6).

To generate homozygous mice carrying the *EF1α* promoter and *mTert* gene knock‐in with complete *STOP* knockout, F0 *EF1α‐loxP‐STOP‐loxP‐mTert* ^*flox/+*^ heterozygous was mated with heterozygous mice, which were provided by Cyagen Company. The resulting F1 pups were genotyped by PCR. WT *Rosa26* allele was detected by the presence of 292 bp PCR product using forward primer of *5′Arm*_F3 (*5′*‐AGAGTTTAGCCAGCCAGTGGTGGT‐*3′*) and reverse primer of *3′Arm*_R (*5′*‐AAGACCCAACCAACAGCAGAGA‐*3′*). The knock‐in transgene was detected by appearance of 520 bp DNA using forward primer of *Tert*_F1 (*5′*‐AAGCTCCCAGAGGCGACAATG‐*3′*) and reverse primer of *3′Arm*_R (*5′*‐AAGACCCAACCAACAGCAGAGA‐*3′*). Their transgenic status was further confirmed by DNA sequencing conducted by Tsingke Biotechnology Co., Ltd.

The *B6.FVB‐Tg(EIIa‐Cre)C5379Lmgd/J* transgenic mice, also known as *EIIa*‐Cre mice, were obtained from the Jackson Laboratory and confirmed by PCR for the presence of 100 bp band using *Cre*_F primer (*5′*‐GCGGTCTGGCAGTAAAAACTATC‐*3′*) and Cre_R primer (*5′*‐GTGAAACAGCATTGCTGTCACTT‐*3′*) (Lakso et al., 1996). These mice, carrying the *EIIa* promoter‐driven Cre recombinase and selectively expressing Cre in preimplantation embryos, were used to remove STOP in all cells in early embryos. F1 male homozygous mice were bred with *EIIa*‐Cre female to induce the removal of the *STOP* gene, resulting in the generation of G0 *EF1α‐mTert* ^*flox/+*^ heterozygotes with the *STOP* gene deletion. The genotypes of the resulting G0 pups were identified by PCR analysis (Figure S8). Floxed mice were positive for 472 bp PCR products whereas 1349 bp DNA was in present in non‐floxed mice using PCR primer of *EF1α*_F2 (*5′*‐CCAGGCACCTCGATTAGTTC‐*3′*) and Tert_R3 (*5′*‐AGTGCGGTAGATCTTCGGGTC‐*3′*).

G1 *EF1α‐mTert* ^*flox/flox*^ homozygous pups, also known as *C57BL/6‐Rosa26* ^tm1.1.n(*EF1α‐mTert/EF1α‐mTert*)^ mice or *EF1α‐mTert* ^*flox*^ *_Rosa26* ^*wt*^ */EF1α‐mTert* ^*flox*^ *_Rosa26* ^*wt*^ mice, were generated by mating *EF1α‐mTert* ^*flox/+*^ heterozygotes. Genotyping of G1 pups was performed using PCR, 520 bp DNA using *Tert*_F1 (*5′*‐AAGCTCCCAGAGGCGACAATG‐*3′*) and *3′Arm*_R (*5′*‐AAGACCCAACCAACAGCAGAGA‐*3′*), 1636 bp (not 2512 bp) DNA using *5′Arm*_F3 (*5′*‐AGAGTTTAGCCAGCCAGTGGTGGT‐*3′*) and *Tert*_R3 (*5′*‐AGTGCGGTAGATCTTCGGGTC‐*3′*), and 472 bp PCR products using primers of *EF1α*_F2 (*5′*‐CCAGGCACCTCGATTAGTTC‐*3′*) and *Tert*_R3 (*5′*‐AGTGCGGTAGATCTTCGGGTC‐*3′*). The transgene was confirmed by sequencing (Figure S9). Subsequent G2, G3, G4 and G5 generations of homozygous mice were obtained through inbreeding of *EF1α‐mTert* ^*flox/flox*^ individuals, referred to as *TertKI* mice. Genotyping of G2–G5 pups was carried out using the same PCR method described previously. The *Tert* mouse line, with confirmed systemic activation of the *Tert* gene, has been deposited at the NJUCM Laboratory Animal Center and is available under the catalog numbers: G1 *TertKI* mice, G2 *TertKI* mice, G3 *TertKI* mice and G4 *TertKI* mice. This breeding strategy involving consecutive generations of full‐sib mating ensures the maintenance and preservation of the *Tert* mouse line.

### 4.8 Transcriptomic and phenotypic assessments

*Tert* transgenic and WT mice were housed in separate groups under controlled environmental conditions. The mice were visually assessed for any discernible differences in coat color, body size, or other visible characteristics. Their locomotor activity and exploration in an open‐field arena were recorded. Social interactions, such as sniffing, grooming, and play behavior, were also monitored. Body weights of the mice were regularly measured using a calibrated weighing scale. At the age of 6 months, *TertKI* and control mice (with a minimum of 5 animals per group per sex) were euthanized. The heart, liver, spleen, lung, kidney, brain, testis, etc. were dissected and weighed, and the organ‐to‐body weight ratios were calculated.

RT‐qPCR was performed to measure the transcription levels of *Tert* in *TertKI* mice. Total RNA was extracted from the isolated tissues, and then converted into single strand cDNA through reverse transcription. The cDNA was used as a template in qPCR reactions, where specific primers *Tert*_F2 (*5′*‐GGATTGCCACTGGCTCCG‐*3′*) and *Tert*_R2 (*5′*‐TGCCTGACCTCCTCTTGTGAC‐*3′*) were utilized to amplify 279 bp DNA from *Tert* gene. The fluorescence signal generated during each qPCR cycle was monitored, allowing for the quantification of *Tert* transcript levels. The relative expression of *Tert* was determined using 114 bp PCR product from transcripts of the *Gapdh* house keeping gene, using primer *Gapdh_*F (*5′*‐AATGGTGAAGGTCGGTGTGAAC‐*3′*) and *Gapdh*_R (*5′*‐AGGTCAATGAAGGGGTCGTTG‐*3′*).

Telomerase activity in mouse organs was assessed using the Telomerase Activity Quantification qPCR Assay Kit (3H Biomedical AB, Uppsala, Sweden, Cat. No. SC8928) according to the manufacturer's instructions (Péntek et al., 2023). This kit utilizes a qPCR‐based method to measure telomerase activity. Briefly, tissue samples were homogenized, and proteins were extracted and quantified. The telomerase reaction mixture containing the extracted telomerase, telomerase substrate, and PCR reagents were subjected to qPCR amplification. The amplification curves were analyzed to determine the telomerase activity in the samples.

The telomere length in mouse organs was measured using the TeloTAGGG Telomere Length Assay Kit (Sigma‐Aldrich, Darmstadt, Germany) following the manufacturer's instructions (Looi et al., 2010). Genomic DNA was extracted from the organs, and telomere‐specific DNA fragments were obtained by digestion with restriction enzymes. The fragments were then separated by gel electrophoresis, transferred to a membrane, and hybridized with a telomere‐specific probe. The telomere length was determined by comparing the migration pattern of the fragments with a molecular weight marker.

### 4.9 Adverse effects in *TertKI* mice

In *TertKI* mice, the genetic toxicity assessment involves evaluating the potential adverse effects on the genetic material (Taškova et al., 2018) caused by the integration and expression of the *Tert* gene. This assessment helps identify any DNA damage, mutations, or chromosomal abnormalities induced by the *TertKI*, providing valuable insights into the integrity and safety of the mouse genetic material, with the following analyses.

After sacrificing the male mice, the right epididymis was isolated and washed with normal saline. It was then cut into small pieces and transferred to a saline solution. The suspension was gently blown through a straw multiple time to release the sperm. After a 5‐min incubation, the suspension was filtered and placed on a glass slide. The sperm was air‐dried and fixed with methanol for 15 min. Subsequently, the slides were stained with a 2% eosin solution for 1 h. After washing with water, the slides were examined under a light microscope. A subset of sperm (less than 500) per animal was evaluated to identify any abnormalities in their morphology or structure.

The thighbones of mice were dissected and the bone marrow was extracted by squeezing using hemostatic forceps. The obtained marrow was mixed with FBS and smeared onto glass slides, followed by air‐drying. The cells on the slides were fixed with methanol for 5–10 min. Giemsa solution (http://www.balbsw.com/, Beijing, China, Cat. No. GL0812) was then applied for 5–10 min for cell staining, followed by washing with PBS solution. The presence of micronucleated cells was observed under a light microscope. A total of 1000 polychromatic erythrocytes per animal were scored to assess the mutagenic properties.

To ascertain the impact of genetic modification on overall health and organ function, a comparative analysis of biochemical markers was conducted between the transgenic mice and the control group. Routine blood tests measured blood cell counts and parameters, while biochemical tests analyzed specific biomarkers related to organ function. These tests provide valuable insights into the physiological status and organ health of mice. Fresh blood samples were collected and divided into anticoagulant tubes and coagulation tubes. The blood samples in the anticoagulant tubes were used for routine blood tests using the PE‐6800VET automatic hemocyte analyzer (PROKAN, Shenzhen, China). The blood samples collected in the coagulation tubes could clot, and the serum was separated by centrifugation. Biochemical analysis was performed on serum samples using the AU 480 automatic biochemical analyzer (Beckman, USA) to measure mouse Gpt, mouse Got1, and urea levels, following the provided instructions.

Considering the established role of Tert activation in promoting development, progression, and increased cancer incidence (Lansdorp, 2022), we sought to investigate the impact of *Tert*‐overexpression on carcinogenesis in mice under normal culture conditions. We specifically focused on evaluating the potential effects on concentration of CA72‐4, a conventional serum tumor marker for gastric and colorectal cancers (Zhang et al., 2019), CA72‐4 in serum samples collected from both WT mice and *TertKI* mice at different age groups (18 months old and 6 weeks old). The Mouse CA72‐4 ELISA Kit (ZCI BIO, Shanghai, China, Cat. No. ZC‐39023) was utilized for this analysis, enabling us to examine the association between *Tert* expression and the levels of CA72‐4 according to the manufacturer's instructions. Briefly, the CA72‐4 levels were measured by adding the samples to the designated wells of the ELISA plate coated with specific antibodies against CA72‐4. After incubation and washing steps, an enzyme‐linked detection antibody was added, followed by the addition of a substrate solution. The reaction was stopped, and the absorbance was measured using a microplate reader. The CA72‐4 concentration in each sample was determined by comparing the absorbance to a standard curve generated using known concentrations of CA72‐4.

### 4.10 Urethane‐induced lung cancer progression

Previous studies indicate that *Tert* expression is usually turned off in most somatic cells but is activated in cells that need to proliferate continuously or in cancerous cells where it contributes to cellular immortality (Heidenreich et al., 2014). The lung cancer model was established in 8‐week‐old male *TertKI* (G8, *n* = 3) mice and WT (C57BL/6, *n* = 3) mice. Both groups were injected intraperitoneally with 800 mg/kg urethane (CSNpharm, Chicago, USA) twice a week for 112 days. Throughout the treatment period, body weight was recorded. After execution, the morphology and histopathology of the lung, liver, kidney, colon, and stomach were examined to assess lesion development. Histological analysis was performed as previously described to ensure consistency and accuracy. Additionally, the concentration of CA72‐4 in the serum of the lung cancer model was determined following established protocols.

### 4.11 Monitoring lifespan and assessing antioxidant levels

*TertKI* and WT mice were maintained in a controlled laboratory environment with regulated temperature, humidity, and a 12‐h light–dark cycle. They had unrestricted access to standard rodent food and water. The health and well‐being of the mice were regularly monitored throughout their lifespan. Daily observations were made to identify signs of aging, illness, or natural death. Survival data were recorded, and Kaplan–Meier survival curves were constructed using statistical analysis software (Barakat et al., 2019; Kaplan & Meier, 1958). These survival curves allowed for the estimation of median life expectancy and maximal life expectancy (the age reached by the longest‐lived individual in the population) for the studied mouse cohorts.

To evaluate the antioxidant levels in *TertKI* mice, SOD and GSH levels in the liver were measured using the CheKine™ Superoxide SOD Activity Assay kit and the Reduced GSH Colorimetric Assay Kit Caspase‐1 (Abbkine Scientific Co., Ltd., Wuhan, China, Cat. No. KTA3020) according to the manufacturer's instructions. The activity of SOD was measured by monitoring the reduction of a tetrazolium salt to formazan at 450 nm, while the GSH levels were determined by measuring the absorbance of the reaction product at 412 nm. The optical densities at the respective wavelengths were recorded for data analyses by the Victor X Multimode Plate Reader (PerkinElmer, USA) (Logozzi et al., 2020).

### 4.12 Skin injury repair

WT and *TertKI* mice were anesthetized, and the back area was disinfected. A tissue biopsy device with a 4 mm diameter was used to create a full‐thickness skin injury on the dorsal region, extending down to the fascia. The progression of wound healing was monitored and documented at regular intervals, and photographs were taken. The wound areas were measured using ImageJ software (National Institutes of Health, Bethesda, USA), and the percentage of wound closure was calculated using the formula: $\text{Wound healing rate}\left(\%\right)=\frac{\text{Wound area}\;\mathrm{on}\;\mathrm{day}\;0-\text{Wound area}\;\mathrm{on}\;\mathrm{day}\;N}{\text{wound area}\;\mathrm{on}\;\mathrm{day}\;0}\times 100$, both measured in square millimeters (mm^2^) (Galiano et al., 2004).

At 4, 7, and 10 days post‐injury, mice were humanely euthanized, and the skin tissues around the wound were cut by 3 mm. These tissue samples were subsequently divided into three fractions to facilitate various analyses, including histological examination, assessment of extracellular matrix (ECM) components, and measurement of hydroxyproline (HYP) levels.

Histological evaluation was conducted to assess tissue damage, inflammation, and repair processes. The tissues were fixed in 4% paraformaldehyde solution (Biosharp, China) for 24 h on the days 5 and 8 after trauma. After dehydration and paraffin embedding according to the standard procedure, 4 μm sections were cut with a pathological microtome (Leica, China). Then H&E staining and Masson staining were carried out to observe the wound healing rate and collagen fiber arrangement. The positive area of collagen fibers in Masson staining was calculated using Image J software.

Cell scratching assay was performed to evaluate cell migration and wound healing ability in vitro (Martinotti & Ranzato, 2020). Cells were seeded in 6‐well plates at a density of 800,000 cells per well and allowed to reach 90% confluency. A sterile pipette tip was used to create a narrow wound‐like gap by scratching the cell monolayer. Photographs of the scratched area were taken immediately after scratching (0 h) and after 24 h. The wound area was analyzed using ImageJ software to measure the extent of wound closure, and the healing rate was calculated based on the reduction in wound area over time.

Fibroblasts play a crucial role in the process of tissue repair. When a wound occurs, fibroblasts are recruited to the site of injury and contribute to various aspects of the healing process, including the production of ECM, synthesis of growth factors and cytokines, and promotion of angiogenesis (Reinke & Sorg, 2012). Their coordinated actions contribute to tissue remodeling and restoration of tissue integrity. Newborn mice were sacrificed, and their dorsal skin was separated and cut into 1 mm^2^ size. The small skin patches were then subjected to digestion using Type I collagen (Sigma‐Aldrich) (1 mg/mL) at 37°C for 30 min, followed by trypsin (0.25 mg/mL) at 37°C for 40 min. The digested fibroblasts from the mouse skins were subsequently cultured in DMEM complete medium supplemented with 20% fetal bovine serum (GIBCO, Grand Island, USA) at 37°C in a 5% CO_2_ incubator. The isolated fibroblasts were further characterized using immunofluorescence staining with an antibody against Vimentin proteins (SAB Signalway Antibody) to confirm their identity as fibroblasts.

The ECM is essential for tissue repair as it provides structural support, promotes cell adhesion, and guides cell migration during the healing process (Hynes, 2009). Collagen, the main component of ECM, contributes to tissue strength and integrity. Hydroxyproline, an amino acid found in collagen, is crucial for collagen synthesis and stability (Diegelmann & Evans, 2004). To assess the ECM content, hydroxyproline was quantified using a hydroxyproline assay kit (Solarbio LIFE SCIENCES, Beijing, China) according to the manufacturer's instructions. We used wound hydroxyproline content to assess collagen levels, measured in μg of hydroxyproline per mg of dry wound tissue. (Caetano et al., 2016). The tissue was weighed and hydrolyzed with 6 mol/L hydrochloric acid. The hydroxyproline content was determined using the chloramine T method with slight modifications. Briefly, the hydrolysate was adjusted to pH 6–8 using 10 mol/L NaOH. Subsequently, chloramine T solution was added, and the mixture was incubated at room temperature (25°C) for 20 min. After that, dimethylaminobenzaldehyde (DMAB) solution was added, and the mixture was further incubated at 60°C for 20 min. The reaction mixture was then cooled for 15 min, and the absorbance was measured at 595 nm using a Victor X3 microplate reader (PerkinElmer, Waltham, USA) (Stegemann & Stalder, 1967).

Fibroblasts play a significant role in ECM synthesis by producing growth factors such as Fgf (Frantz et al., 2010). These growth factors stimulate cell proliferation and ECM synthesis. Furthermore, fibroblasts contribute to the formation of new blood vessels, a process known as angiogenesis. Collagen I and collagen III are the most abundant proteins in ECM of connective tissues, including the skin. IL‐1β and Tnf‐α are two inflammatory cytokines released by immune cells, such as macrophages, in response to tissue injury. They initiate and amplify the inflammatory response, and promote the formation of new blood vessels by inducing the expression of pro‐angiogenic factors, such as Vegf (Eming et al., 2007). Vegf acts as a potent inducer of angiogenesis by stimulating the proliferation, migration, and tube formation of endothelial cells, which are the building blocks of blood vessels (Folkman, 1995). Wnt‐1 is a signaling molecule that is known to promote re‐epithelialization, granulation tissue formation, and ECM remodeling during the wound healing process (Rognoni & Watt, 2018). The mRNA expression levels of inflammatory factors and growth factors, including *Il1β*, *Tnfα*, *Fgf7*, *Col1α1* (encoding Collagen I), and *Col3α1* (encoding Collagen III), were measured using the RT‐qPCR method using primers listed in Table S4. Similarly, the protein levels of Fgf7, Vegf, and Wnt‐1 were assessed using Western blotting.

### 4.13 Colon damage repair

Both *TertKI* (experimental group) and WT mice (control group) were divided and subjected to DSS treatment, with drinking water containing 3% DSS for seven consecutive days. The mice were monitored daily for changes in stool consistency and the presence of blood in the stool, as indicators of colon injury and inflammation. After the DSS treatment, the mice were sacrificed, and the length of the colon was measured as a parameter to assess the extent of injury and healing and evaluate the impact of *TertKI* on the response to DSS‐induced colon injury. Routine staining and histological evaluation were performed on colon sections to evaluate the degree of colon injury, inflammation, and repair.

### 4.14 Statistics analysis

The statistical analysis of the data was conducted using appropriate methods to assess the significance of the findings. Descriptive statistics, including mean values and standard deviations, were calculated for each group to summarize the data. To compare the differences between two columns, *t* test was performed. To compare the differences between multiple columns, one‐way analysis of variance (ANOVA) was performed. To compare the differences between multiple groups, two‐way ANOVA was performed. This analysis allowed for the examination of significant variations among the groups. Statistical significance was determined using a *p*‐value threshold of <0.05, indicating a significant difference between groups and a low probability of obtaining the observed results by chance. In addition, survival analysis was carried out using the Kaplan–Meier method to assess the survival rates between different experimental groups. This method is commonly used to estimate and compare survival distributions. All statistical analyses were performed using the GraphPad Prism 8.0 software (https://www.graphpad.com/), a widely used tool for data analysis and visualization in scientific research.

## AUTHOR CONTRIBUTIONS

Y. Pan, G‐M. Yang, P. Hu, and S‐B. Shen conceived the idea contributed to the study design and data acquisition and analysis. T‐Y. Zhu conceived the idea, contributed to study design, and acquired initial funding. J‐L. Zhang, A‐N. Xu, Y‐H. Mi, M‐T. Gao, and Y‐Y. Zhang performed the experiments and were involved in data acquisition and analysis. J‐L. Zhang, Y‐H. Mi, and A‐N. Xu worked on aspects of the study relating to the generation of *mTert* transgenic mice, expression of telomerase reverse transcriptase (Tert), and prolonging lifespan and accelerating damage repair in mice. Y. Pan, P. Hu, G‐M. Yang, and S‐B. Shen drafted the paper. Y. Pan, T‐Y. Zhu, P. Hu, G‐M. Yang, and S‐B. Shen reviewed and edited the paper. All authors have read and approved the final manuscript.

## CONFLICT OF INTEREST STATEMENT

None declared.

## Supporting information


Appendix S1.

Tables S1‐S4.

Figures S1‐S15.

## Data Availability

Data generated or analyzed during this study are included in this published article, its Supplementary Information, and Source Data files. All other data pertaining to this study are available from the corresponding author upon reasonable request.
