# Supplementary material for: Telomerase reverse transcriptase gene knock‐in unleashes enhanced longevity and accelerated damage repair in mice
Source: Aging Cell. 2024 Dec 11;24(4):e14445. doi: 10.1111/acel.14445 (PMC11984681; doi:10.1111/acel.14445)
Supplement: Supplementary file 1 — Appendix S1. [file ACEL-24-e14445-s003.pdf]

Supplementary Documents

Supplementary Document 1. Key features and DNA sequence of the final targeting vector (20793 bp).

Location (bp)	Feature	Size (bp)	Label	
			Highlight	Font color
1–2660	Plasmid vector sequence	2660		
2661–5307	*Rosa26 5'Arm*	2647	Green	
5308–6485	Human *EF1α* promoter	1178	Yellow	
6513–6547	*loxP*	35		Red/Bold
6548–7389	STOP	842	Grey	
7390–7424	*LoxP*	35		Red/Bold
7462–10830	Mouse *Tert* cDNA	3369	Cyan	
10831–11085	*HSV-TK* poly (A) signal	255	Grey	
11149–11180	*Rox* site	32		Purple/Bold
11279–11978	Mouse *tACE* promoter	700	Yellow	
11979–13028	*iDreV* light-inducible DNA recombinase	1050	Red	
13029–13168	*SV40* polyA signal	140		
13169–13773	*TK* promoter	605	Yellow	
13774–14577	*Neo*-resistant gene	804	Pink	
14609–14822	*BGH* polyA	214	Grey	
14823–14945	*Neor*-derived sequence	123	Black	
14946–14977	*Rox* site	32		Purple/Bold
14984–19090	*Rosa26 3'Arm*	4107	Green	
19091–20793	Plasmid vector sequence	1703		

TTAATATTTTGTTAAAATTCGCGTTAAATTTTTGTTAAATCAGCTCATTTTTTAACCAATAGGCCGAAATCGGCAA

AATCCCTTATAAATCAAAAGAATAGACCGAGATAGGGTTGAGTGTTGTTCCAGTTTGGAACAAGAGTCCACTATTA

AAGAACGTGGACTCCAACGTCAAAGGGCGAAAAACCGTCTATCAGGGCGATGGCCCACTACGTGAACCATCACCCT

AATCAAGTTTTTTGGGGTCGAGGTGCCGTAAAGCACTAAATCGGAACCCTAAAGGGAGCCCCCGATTTAGAGCTTG

ACGGGGAAAGCCGGCGAACGTGGCGAGAAAGGAAGGGAAGAAAGCGAAAGGAGCGGGCGCTAGGGCGCTGGCAAGT

GTAGCGGTCACGCTGCGCGTAACCACCACACCCGCCGCGCTTAATGCGCCGCTACAGGGCGCGTCAGGTGGCACTT

TTCGGGGAAATGTGCGCGGAACCCCTATTTGTTTATTTTTCTAAATACATTCAAATATGTATCCGCTCATGAGACA

ATAACCCTGATAAATGCTTCAATAATATTGAAAAAGGAAGAGTATGAGTATTCAACATTTCCGTGTCGCCCTTATT

CCCTTTTTTGCGGCATTTTGCCTTCCTGTTTTTGCTCACCCAGAAACGCTGGTGAAAGTAAAAGATGCTGAAGATC

AGTTGGGTGCACGAGTGGGTTACATCGAACTGGATCTCAACAGCGGTAAGATCCTTGAGAGTTTTCGCCCCGAAGA

ACGTTTTCCAATGATGAGCACTTTTAAAGTTCTGCTATGTGGCGCGGTATTATCCCGTATTGACGCCGGGCAAGAG

CAACTCGGTCGCCGCATACACTATTCTCAGAATGACTTGGTTGAGTACTCACCAGTCACAGAAAAGCATCTTACGG

ATGGCATGACAGTAAGAGAATTATGCAGTGCTGCCATAACCATGAGTGATAACACTGCGGCCAACTTACTTCTGAC

AACGATCGGAGGACCGAAGGAGCTAACCGCTTTTTTGCACAACATGGGGGATCATGTAACTCGCCTTGATCGTTGG

GAACCGGAGCTGAATGAAGCCATACCAAACGACGAGCGTGACACCACGATGCCTGTAGCAATGGCAACAACGTTGC

GCAAACTATTAACTGGCGAACTACTTACTCTAGCTTCCCGGCAACAATTAATAGACTGGATGGAGGCGGATAAAGT

TGCAGGACCACTTCTGCGCTCGGCCCTTCCGGCTGGCTGGTTTATTGCTGATAAATCTGGAGCCGGTGAGCGTGGG

TCTCGCGGTATCATTGCAGCACTGGGGCCAGATGGTAAGCCCTCCCGTATCGTAGTTATCTACACGACGGGGAGTC

AGGCAACTATGGATGAACGAAATAGACAGATCGCTGAGATAGGTGCCTCACTGATTAAGCATTGGTAACTGTCAGA

CCAAGTTTACTCATATATACTTTAGATTGATTTAAAACTTCATTTTTAATTTAAAAGGATCTAGGTGAAGATCCTT
TTTGATAATCTCATGACCAAAATCCCTTAACGTGAGTTTTCGTTCCACTGAGCGTCAGACCCCGTAGAAAAGATCA
AAGGATCTTCTTGAGATCCTTTTTTTCTGCGCGTAATCTGCTGCTTGCAAACAAAAAAACCACCGCTACCAGCGGT
GGTTTGTTTGCCGGATCAAGAGCTACCAACTCTTTTTCCGAAGGTAACTGGCTTCAGCAGAGCGCAGATACCAAAT
ACTGTTCTTCTAGTGTAGCCGTAGTTAGGCCACCACTTCAAGAACTCTGTAGCACCGCCTACATACCTCGCTCTGC
TAATCCTGTTACCAGTGGCTGCTGCCAGTGGCGATAAGTCGTGTCTTACCGGGTTGGACTCAAGACGATAGTTACC
GGATAAGGCGCAGCGGTCGGGCTGAACGGGGGGTTCGTGCACACAGCCCAGCTTGGAGCGAACGACCTACACCGAA
CTGAGATACCTACAGCGTGAGCTATGAGAAAGCGCCACGCTTCCCGAAGGGAGAAAGGCGGACAGGTATCCGGTAA
GCGGCAGGGTCGGAACAGGAGAGCGCACGAGGGAGCTTCCAGGGGGAAACGCCTGGTATCTTTATAGTCCTGTCGG
GTTTCGCCACCTCTGACTTGAGCGTCGATTTTTGTGATGCTCGTCAGGGGGGCGGAGCCTATGGAAAAACGCCAGC
AACGCGGCCTTTTTACGGTTCCTGGCCTTTTGCTGGCCTTTTGCTCACATGTTCTTTCCTGCGTTATCCCCTGATT
CTGTGGATAACCGTATTACCGCCTTTGAGTGAGCTGATACCGCTCGCCGCAGCCGAACGACCGAGCGCAGCGAGTC
AGTGAGCGAGGAAGCGGAAGAGCGCCCAATACGCAAACCGCCTCTCCCCGCGCGTTGGCCGATTCATTAATGCAGC
TGGCACGACAGGTTTCCCGACTGGAAAGCGGGCAGTGAGCGCAACGCAATTAATGTGAGTTAGCTCACTCATTAGG
CACCCCAGGCTTTACACTTTATGCTTCCGGCTCGTATGTTGTGTGGAATTGTGAGCGGATAACAATTTCACACAGG
AAACAGCTATGACCATGATTACGCCAAGCTCGAAATTAACCCTCACTAAAGGGAACAAAAGCTGGTACGCGGCCGC
TCGTCGTCTGATTGGCTCTCGGGGCCCAGAAAACTGGCCCTTGCCATTGGCTCGTGTTCGTGCAAGTTGAGTCCAT
CCGCCGGCCAGCGGGGGCGGCGAGGAGGCGCTCCCAGGTTCCGGCCCTCCCCTCGGCCCCGCGCCGCAGAGTCTGG
CCGCGCGCCCCTGCGCAACGTGGCAGGAAGCGCGCGCTGGGGGCGGGGACGGGCAGTAGGGCTGAGCGGCTGCGGG
GCGGGTGCAAGCACGTTTCCGACTTGAGTTGCCTCAAGAGGGGCGTGCTGAGCCAGACCTCCATCGCGCACTCCGG
GGAGTGGAGGGAAGGAGCGAGGGCTCAGTTGGGCTGTTTTGGAGGCAGGAAGCACTTGCTCTCCCAAAGTCGCTCT
GAGTTGTTATCAGTAAGGGAGCTGCAGTGGAGTAGGCGGGGAGAAGGCCGCACCCTTCTCCGGAGGGGGGAGGGGA
GTGTTGCAATACCTTTCTGGGAGTTCTCTGCTGCCTCCTGGCTTCTGAGGACCGCCCTGGGCCTGGGAGAATCCCT
TCCCCCTCTTCCCTCGTGATCTGCAACTCCAGTCTTTCTAGAAGATGGGCGGGAGTCTTCTGGGCAGGCTTAAAGG
CTAACCTGGTGTGTGGGCGTTGTCCTGCAGGGGAATTGAACAGGTGTAAAATTGGAGGGACAAGACTTCCCACAGA
TTTTCGGTTTTGTCGGGAAGTTTTTTAATAGGGGCAAATAAGGAAAATGGGAGGATAGGTAGTCATCTGGGGTTTT
ATGCAGCAAAACTACAGGTTATTATTGCTTGTGATCCGCCTCGGAGTATTTTCCATCGAGGTAGATTAAAGACATG
CTCACCCGAGTTTTATACTCTCCTGCTTGAGATCCTTACTACAGTATGAAATTACAGTGTCGCGAGTTAGACTATG
TAAGCAGAATTTTAATCATTTTTAAAGAGCCCAGTACTTCATATCCATTTCTCCCGCTCCTTCTGCAGCCTTATCA
AAAGGTATTTTAGAACACTCATTTTAGCCCCATTTTCATTTATTATACTGGCTTATCCAACCCCTAGACAGAGCAT
TGGCATTTTCCCTTTCCTGATCTTAGAAGTCTGATGACTCATGAAACCAGACAGATTAGTTACATACACCACAAAT
CGAGGCTGTAGCTGGGGCCTCAACACTGCAGTTCTTTTATAACTCCTTAGTACACTTTTTGTTGATCCTTTGCCTT
GATCCTTAATTTTCAGTGTCTATCACCTCTCCCGTCAGGTGGTGTTCCACATTTGGGCCTATTCTCAGTCCAGGGA
GTTTTACAACAATAGATGTATTGAGAATCCAACCTAAAGCTTAACTTTCCACTCCCATGAATGCCTCTCTCCTTTT
TCTCCATTTATAAACTGAGCTATTAACCATTAATGGTTTCCAGGTGGATGTCTCCTCCCCCAATATTACCTGATGT
ATCTTACATATTGCCAGGCTGATATTTTAAGACATTAAAAGGTATATTTCATTATTGAGCCACATGGTATTGATTA
CTGCTTACTAAAATTTTGTCATTGTACACATCTGTAAAAGGTGGTTCCTTTTGGAATGCAAAGTTCAGGTGTTTGT
TGTCTTTCCTGACCTAAGGTCTTGTGAGCTTGTATTTTTTCTATTTAAGCAGTGCTTTCTCTTGGACTGGCTTGAC
TCATGGCATTCTACACGTTATTGCTGGTCTAAATGTGATTTTGCCAAGCTTCTTCAGGACCTATAATTTTGCTTGA
CTTGTAGCCAAACACAAGTAAAATGATTAAGCAACAAATGTATTTGTGAAGCTTGGTTTTTAGGTTGTTGTGTTGT
GTGTGCTTGTGCTCTATAATAATACTATCCAGGGGCTGGAGAGGTGGCTCGGAGTTCAAGAGCACAGACTGCTCTT
CCAGAAGTCCTGAGTTCAATTCCCAGCAACCACATGGTGGCTCACAACCATCTGTAATGGGATCTGATGCCCTCTT
CTGGTGTGTCTGAAGACCACAAGTGTATTCACATTAAATAAATAAATCCTCCTTCTTCTTCTTTTTTTTTTTTTTA
AAGAGAATACTGTCTCCAGTAGAATTTACTGAAGTAATGAAATACTTTGTGTTTGTTCCAATATGGTAGCCAATAA

TCAAATTACTCTTTAAGCACTGGAAATGTTACCAAGGAACTAATTTTTATTTGAAGTGTAACTGTGGACAGAGGAG
CCATAACTGCAGACTTGTGGGATACAGAAGACCAATGCAGACTTTAATGTCTTTTCTCTTACACTAAGCAATAAAG
AAATAAAAAATTGAACTTCTAGTATCCTATTTGTTTAAACTGCTAGCTTTACTTAACTTTTGTGCTTCATCTATACA
AAGCTGAAAGCTAAGTCTGCAGCCATTACTAAACATGAAAGCAAGTAATGATAATTTTGGATTTCAAAAATGTAGG
GCCAGAGTTTAGCCAGCCAGTGGTGGTGCTTGCCTTTATGCCTTTAATCCCAGCACTCTGGAGGCAGAGACAGGCA
GATCTCTGAGTTTGAGCCCAGCCTGGTCTACACATCAAGTTCTATCTAGGATAGCCAGGAATACACACAGAAACCC
TGTTGGGGAGGGGGGCTCTGAGATTTCATAAAATTATAATTGAAGCATTCCCTAATGAGCCAC**GTCGACGGCTCCG**
GTGCCCGTCAGTGGGCAGAGCGCACATCGCCCACAGTCCCCGAGAAGTTGGGGGGAGGGGTCGGCAATTGAACCGG
TGCCTAGAGAAGGTGGCGCGGGGTAAACTGGGAAAGTGATGTCGTGTACTGGCTCCGCCTTTTTCCCGAGGGTGGG
GGAGAACCGTATATAAGTGCAGTAGTCGCCGTGAACGTTCTTTTTCGCAACGGGTTTGCCGCCAGAACACAGGTAA
GTGCCGTGTGTGGTTCCCGCGGGCCTGGCCTCTTTACGGGTTATGGCCCTTGCGTGCCTTGAATTACTTCCACCTG
GCTGCAGTACGTGATTCTTGATCCCGAGCTTCGGGTTGGAAGTGGGTGGGAGAGTTCGAGGCCTTGCGCTTAAGGA
GCCCCTTCGCCTCGTGCTTGAGTTGAGGCCTGGCCTGGGCGCTGGGGCCGCCGCGTGCGAATCTGGTGGCACCTTC
GCGCCTGTCTCGCTGCTTTCGATAAGTCTCTAGCCATTTAAAATTTTTGATGACCTGCTGCGACGCTTTTTTTCTG
GCAAGATAGTCTTGTAAATGCGGGCCAAGATCTGCACACTGGTATTTCGGTTTTTGGGGCCGCGGGCGGCGACGGG
GCCCGTGCGTCCCAGCGCACATGTTCGGCGAGGCGGGGCCTGCGAGCGCGGCCACCGAGAATCGGACGGGGGTAGT
CTCAAGCTGGCCGGCCTGCTCTGGTGCCTGGTCTCGCGCCGCCGTGTATCGCCCCGCCCTGGGCGGCAAGGCTGGC
CCGGTCGGCACCAGTTGCGTGAGCGGAAAGATGGCCGCTTCCCGGCCCTGCTGCAGGGAGCTCAAAATGGAGGACG
CGGCGCTCGGGAGAGCGGGCGGGTGAGTCACCCACACAAAGGAAAAGGGCCTTTCCGTCCTCAGCCGTCGCTTCAT
GTGACTCCACGGAGTACCGGGCGCCGTCCAGGCACCTCGATTAGTTCTCGAGCTTTTGGAGTACGTCGTCTTTAGG
TTGGGGGGAGGGGTTTTATGCGATGGAGTTTCCCCACACTGAGTGGGTGGAGACTGAAGTTAGGCCAGCTTGGCAC
TTGATGTAATTCTCCTTGGAATTTGCCCTTTTTGAGTTTGGATCTTGGTTCATTCTCAAGCCTCAGACAGTGGTTC
AAAGTTTTTTTCTTCCATTTCAGGTGTCGTGAGGCTGCAGGTCGAGGGACCT**AATAACTTCGTATAGCATACATTA**
TACGAAGTTATATTAAGGGTTCCGGATCAGCTTGATGGGGATCCAGACATGATAAGATACATTGATGAGTTTGGAC
AAACCACAACTAGAATGCAGTGAAAAAAATGCTTTATTTGTGAAATTTGTGATGCTATTGCTTTATTTGTAACCAT
TATAAGCTGCAATAAACAAGTTAACAACAACAATTGCATTCATTTTATGTTTCAGGTTCAGGGGGAGGTGTGGGAG
GTTTTTTAAAGCAAGTAAAACCTCTACAAATGTGGTATGGCTGATTATGATCCTCTAGAGTCGCAGATCCAGACAT
GATAAGATACATTGATGAGTTTGGACAAACCACAACTAGAATGCAGTGAAAAAAATGCTTTATTTGTGAAATTTGT
GATGCTATTGCTTTATTTGTAACCATTATAAGCTGCAATAAACAAGTTAACAACAACAATTGCATTCATTTTATGT
TTCAGGTTCAGGGGGAGGTGTGGGAGGTTTTTTAAAGCAAGTAAAACCTCTACAAATGTGGTATGGCTGATTATGA
TCCTCTAGAGTCGCAGATCCAGACATGATAAGATACATTGATGAGTTTGGACAAACCACAACTAGAATGCAGTGAA
AAAAATGCTTTATTTGTGAAATTTGTGATGCTATTGCTTTATTTGTAACCATTATAAGCTGCAATAAACAAGTTAA
CAACAACAATTGCATTCATTTTATGTTTCAGGTTCAGGGGGAGGTGTGGGAGGTTTTTTAAAGCAAGTAAAACCTC
TACAAATGTGGTATGGCTGATTATGATCCTCTAGAGTCGCAGATCCTCTAGAGTCGCAGATCTGCAAGCTAATTCC
TGCAGGTCGAGGGACCT**AATAACTTCGTATAGCATACATTATACGAAGTTAT**ATTAAGGGTTCCGGATCCACTACA
CCACGTGGCCACC**ATG**ACCCGCGCTCCTCGTTGCCCCGCGGTGCGCTCTCTGCTGCGCAGCCGATACCGGGAGGTG
TGGCCGCTGGCAACCTTTGTGCGGCGCCTGGGGCCCGAGGGCAGGCGGCTTGTGCAACCCGGGGACCCGAAGATCT
ACCGCACTTTGGTTGCCCAATGCCTAGTGTGCATGCACTGGGGCTCACAGCCTCCACCTGCCGACCTTTCCTTCCA
CCAGGTGTCATCCCTGAAAGAGCTGGTGGCCAGGGTTGTGCAGAGACTCTGCGAGCGCAACGAGAGAAACGTGCTG
GCTTTTGGCTTTGAGCTGCTTAACGAGGCCAGAGGCGGGCCTCCCATGGCCTTCACTAGTAGCGTGCGTAGCTACT
TGCCCAACACTGTTATTGAGACCCTGCGTGTCAGTGGTGCATGGATGCTACTGTTGAGCCGAGTGGGCGACGACCT
GCTGGTCTACCTGCTGGCACACTGTGCTCTTTATCTTCTGGTGCCCCCCAGCTGTGCCTACCAGGTGTGTGGGTCT
CCCCTGTACCAAATTTGTGCCACCACGGATATCTGGCCCTCTGTGTCCGCTAGTTACAGGCCCACCCGACCCGTGG
GCAGGAATTTCACTAACCTTAGGTTCTTACAACAGATCAAGAGCAGTAGTCGCCAGGAAGCACCGAAACCCCTGGC

CTTGCCATCTCGAGGTACAAAGAGGCATCTGAGTCTCACCAGTACAAGTGTGCCTTCAGCTAAGAAGGCCAGATGC
TATCCTGTCCCGAGAGTGGAGGAGGGACCCCACAGGCAGGTGCTACCAACCCCATCAGGCAAATCATGGGTGCCAA
GTCCTGCTCGGTCCCCCGAGGTGCCTACTGCAGAGAAAGATTTGTCTTCTAAAGGAAAGGTGTCTGACCTGAGTCT
CTCTGGGTCGGTGTGCTGTAAACACAAGCCCAGCTCCACATCTCTGCTGTCACCACCCCGCCAAAATGCCTTTCAG
CTCAGGCCATTTATTGAGACCAGACATTTCCTTTACTCCAGGGGAGATGGCCAAGAGCGTCTAAACCCCTCATTCC
TACTCAGCAACCTCCAGCCTAACTTGACTGGGGCCAGGAGACTGGTGGAGATCATCTTTCTGGGCTCAAGGCCTAG
GACATCAGGACCACTCTGCAGGACACACCGTCTATCGCGTCGATACTGGCAGATGCGGCCCCTGTTCCAACAGCTG
CTGGTGAACCATGCAGAGTGCCAATATGTCAGACTCCTCAGGTCACATTGCAGGTTTCGAACAGCAAACCAACAGG
TGACAGATGCCTTGAACACCAGCCCACCGCACCTCATGGATTTGCTCCGCCTGCACAGCAGTCCCTGGCAGGTATA
TGGTTTTCTTCGGGCCTGTCTCTGCAAGGTGGTGTCTGCTAGTCTCTGGGGTACCAGGCACAATGAGCGCCGCTTC
TTTAAGAACTTAAAGAAGTTCATCTCGTTGGGGAAATACGGCAAGCTATCACTGCAGGAACTGATGTGGAAGATGA
AAGTAGAGGATTGCCACTGGCTCCGCAGCAGCCCGGGGAAGGACCGTGTCCCCGCTGCAGAGCACCGTCTGAGGGA
GAGGATCCTGGCTACGTTCCTGTTCTGGCTGATGGACACATACGTGGTACAGCTGCTTAGGTCATTCTTTTACATC
ACAGAGAGCACATTCCAGAAGAACAGGCTCTTCTTCTACCGTAAGAGTGTGTGGAGCAAGCTGCAGAGCATTGGAG
TCAGGCAACACCTTGAGAGAGTGCGGCTACGGGAGCTGTCACAAGAGGAGGTCAGGCATCACCAGGACACCTGGCT
AGCCATGCCCATCTGCAGACTGCGCTTCATCCCCAAGCCCAACGGCCTGCGGCCCATTGTGAACATGAGTTATAGC
ATGGGTACCAGAGCTTTGGGCAGAAGGAAGCAGGCCCAGCATTTCACCCAGCGTCTCAAGACTCTCTTCAGCATGC
TCAACTATGAGCGGACAAAACATCCTCACCTTATGGGGTCTTCTGTACTGGGTATGAATGACATCTACAGGACCTG
GCGGGCCTTTGTGCTGCGTGTGCGTGCTCTGGACCAGACACCCAGGATGTACTTTGTTAAGGCAGATGTGACCGGG
GCCTATGATGCCATCCCCCAGGGTAAGCTGGTGGAGGTTGTTGCCAATATGATCAGGCACTCGGAGAGCACGTACT
GTATCCGCCAGTATGCAGTGGTCCGGAGAGATAGCCAAGGCCAAGTCCACAAGTCCTTTAGGAGACAGGTCACCAC
CCTCTCTGACCTCCAGCCATACATGGGCCAGTTCCTTAAGCATCTGCAGGATTCAGATGCCAGTGCACTGAGGAAC
TCCGTTGTCATCGAGCAGAGCATCTCTATGAATGAGAGCAGCAGCAGCCTGTTTGACTTCTTCCTGCACTTCCTGC
GTCACAGTGTCGTAAAGATTGGTGACAGGTGCTATACGCAGTGCCAGGGCATCCCCCAGGGCTCCAGCCTATCCAC
CCTGCTCTGCAGTCTGTGTTTCGGAGACATGGAGAACAAGCTGTTTGCTGAGGTGCAGCGGGATGGGTTGCTTTTA
CGTTTTGTTGATGACTTTCTGTTGGTGACGCCTCACTTGGACCAAGCAAAAACCTTCCTCAGCACCCTGGTCCATG
GCGTTCCTGAGTATGGGTGCATGATAAACTTGCAGAAGACAGTGGTGAACTTCCCTGTGGAGCCTGGTACCCTGGG
TGGTGCAGCTCCATACCAGCTGCCTGCTCACTGCCTGTTTCCCTGGTGTGGCTTGCTGCTGGACACTCAGACTTTG
GAGGTGTTCTGTGACTACTCAGGTTATGCCCAGACCTCAATTAAGACGAGCCTCACCTTCCAGAGTGTCTTCAAAG
CTGGGAAGACCATGCGGAACAAGCTCCTGTCGGTCTTGCGGTTGAAGTGTCACGGTCTATTTCTAGACTTGCAGGT
GAACAGCCTCCAGACAGTCTGCATCAATATATACAAGATCTTCCTGCTTCAGGCCTACAGGTTCCATGCATGTGTG
ATTCAGCTTCCCTTTGACCAGCGTGTTAGGAAGAACCTCACATTCTTTCTGGGCATCATCTCCAGCCAAGCATCCT
GCTGCTATGCTATCCTGAAGGTCAAGAATCCAGGAATGACACTAAAGGCCTCTGGCTCCTTTCCTCCTGAAGCCGC
ACATTGGCTCTGCTACCAGGCCTTCCTGCTCAAGCTGGCTGCTCATTCTGTCATCTACAAATGTCTCCTGGACCT
CTGAGGACAGCCCAAAAACTGCTGTGCCGGAAGCTCCCAGAGGCGACAATGACCATCCTTAAAGCTGCAGCTGACC
CAGCCCTAAGCACAGACTTTCAGACCATTTTGGAC**TAA**GCGATCGCACCCGCGGACTAGAGCTGGGGGAGGCTAAC
TGAAACACGGAAGGAGACAATACCGGAAGGAACCCGCGCTATGACGGCAATAAAAAGACAGAATAAAACGCACGGG
TGTTGGGTCGTTTGTTCATAAACGCGGGGTTCGGTCCCAGGGCTGGCACTCTGTCGATACCCCACCGAGACCCCAT
TGGGGCCAATACGCCCGCGTTTCTTCCTTTTCCCCACCCCACCCCCCAAGTTCGGGTGAAGGCCCAGGGCTCGCAG
CCAACGTCGGGGCGGCAGGCCCTGCCATAGCGCATTCTGGTGTCGACGCTAC**TAACTTTAAATAATTGGCATTATT**
TAAAGTTAATCAAGCTTAGCTTGATATCGAATTCCGAAGTTCCTATTCTCTAGAAAGTATAGGAACTTCAGGTCTG
AAGAGGAGTTTACGTCCAGCCAAGCTAGCT**CCATGGGCCAGGCAAATATCCCTTACCAGCCTCACAGAGACCTCCC**
CCACCCCCCGCAACCCTAGAGTTCTTTTACTAGTGAGGGACAAGTGGACAATGGTGCTGTTGTGGCCCCACCCTG
TGTCCCCTGTGCCCACAGTGGTCACTCTGCTTGGCAGGCAGGTGTTGCAGGCTGGCTGCTCCAGGCCCTGGCAGGA

GGTACTGAAGGACCTGGTAGGCTCAGATGCCCTGGATGCCAAGGCACTGCTGGAGTACTTCCAACCGGTCAGCCAG

TGGCTGGAAGAGCAGAATCAGCGGAATGGCGAAGTCCTAGGCTGGCCAGAGAATCAGTGGCGTCCACCGTTACCCG

ACAACTATCCAGAGGGCATTGGTAAAGCTCTGAGTGAGGGTGGACTGGGACCAAGAGAAGTCCTGGCCTCTGGCCT

CTGGCTTCTGGGTCAAAGCCTCAGCATCCTGGTCACTTTGCTGCCAGCTGAGCCCCAGTGTCCTTTGCTTCAGTGC

CAAGCCACCCCTGGGCTCATCCTCAGGGCCCTAAGCAGAAATGGGTATGTCTTTCTCTCAGGGTCCTAGAGACAGT

GTGCCCAAGCCTGAGGGCCCTTGGGGTCAGGCTGGCTGGCACATTGCTCTATGAGGTCACACTGCAGGCTTGGCTC

TTATTGGCCGGTGATGGGAGCTTCAGGGCTCTGCTTTCCTGCGGCCATGCCTAAGAAGAAGAGGAAGGTTTCTGAG

CTGATTATTAGTGGTTCATCTGGTGGATTCCTGCGAAACATCGGCAAAGAGTATCAGGAGGCCGCTGAAAACTTCA

TGAGGTTTATGAATGACCAGGGGGCGTACGCTCCTAACACTTTGAGGGATTTGAGGTTGGTCTTTCATAGCTGGGC

CAGATGGTGCCATGCTCGGCAGCTTGCATGGTTTCCAATTAGTCCTGAAATGGCACGCGAATACTTTCTTCAGTTG

CACGATGCAGACCTGGCCTCCACTACCATCGACAAGCACTATGCTATGCTTAATATGCTTCTGTCCCACTGCGGAC

TGCCACCCTTGTCCGACGACAAGTCAGTGAGTCTTGCCATGAGAAGAATTAGAAGAGAAGCCGCAACCGAAAAGGG

TGAGAGGACAGGACAGGCAATCCCCCTGCGCTGGGACGACCTGAAGCTGCTGGATGTGCTGCTCAGCAGGAGCGAG

CGGCTGGTCGACCTGCGCAACAGGGCTTTCCTGTTCGTAGCCTATAACACCCTCATGAGAATGTCTGAAATATCAC

GCATCAGGGTTGGGGACTTGGATCAGACAGGAGACACAGTGACCCTGCACATCAGTCACACTAAGACAATCACCAC

AGCTGCGGGCCTTGACAAAGTGCTCTCCCGGCGAACCACAGCAGTGCTCAATGACTGGCTGGACGTCAGTGGGCTT

AGAGAACATCCAGACGCTGTGCTCTTCCCACCTATACACCGGTCAAACAAAGCCCGCATTACTACCACGCCCCTGA

CCGCCCCTGCCATGGAGAAGATTTTCAGTGATGCCTGGGTGCTGCTGAACAAACGGGACGCCACCCCCAATAAAGG

GAGGTATAGGACCTGGACCGGCCATTCCGCCAGGGTGGGTGCCGCAATAGACATGGCCGAGAAACAGGTGTCTATG

GTCGAGATTATGCAGGAAGGGACATGGAAGAAGCCTGAAACACTGATGCGGTATCTCAGAAGGGGCGGAGTGTCCG

TGGGAGCCAATTCTCGACTGATGGATAGC**TAA**ACTTGTTTATTGCAGCTTATAATGGTTACAAATAAAGCAATAGC

ATCACAAATTTCACAAATAAAGCATTTTTTTCACTGCATTCTAGTTGTGGTTTGTCCAAACTCATCAATGTATCTT

ATCATGTCTGGCTCTAGCGC**TTTGGCTGCAGGTCGTCGAAATTCTACCGGGTAGGGGAGGCGCTTTTCCCAAGGCA**

GTCTGGAGCATGCGCTTTAGCAGCCCCGCTGGGCACTTGGCGCTACACAAGTGGCCTCTGGCCTCGCACACATTCC

ACATCCACCGGTAGGCGCCAACCGGCTCCGTTCTTTGGTGGCCCCTTCGCGCCACCTTCTACTCCTCCCCTAGTCA

GGAAGTTCCCCCCCGCCCCGCAGCTCGCGTCGTGCAGGACGTGACAAATGGAAGTAGCACGTCTCACTAGTCTCGT

GCAGATGGACAGCACCGCTGAGCAATGGAAGCGGGTAGGCCTTTGGGGCAGCGGCCAATAGCAGCTTTGCTCCTTC

GCTTTCTGGGCTCAGAGGCTGGGAAGGGGTGGGTCCGGGGGCGGGCTCAGGGGCGGGCTCAGGGGCGGGGCGGGCG

CCCGAAGGTCCTCCGGAGGCCCGGCATTCTGCACGCTTCAAAAGCGCACGTCTGCCGCGCTGTTCTCCTCTTCCTC

ATCTCCGGGCCTTTCGACCTGCAGCCTGTTGACAATTAATCATCGGCATAGTATATCGGCATAGTATAATACGACA

AGGTGAGGAACTAAACCATGGGATCGGCCATTGAACAAGATGGATTGCACGCAGGTTCTCCGGCCGCTTGGGTGGA

GAGGCTATTCGGCTATGACTGGGCACAACAGACAATCGGCTGCTCTGATGCCGCCGTGTTCCGGCTGTCAGCGCAG

GGGCGCCCGGTTCTTTTTGTCAAGACCGACCTGTCCGGTGCCCTGAATGAACTGCAGGACGAGGCAGCGCGGCTAT

CGTGGCTGGCCACGACGGGCGTTCCTTGCGCAGCTGTGCTCGACGTTGTCACTGAAGCGGGAAGGGACTGGCTGCT

ATTGGGCGAAGTGCCGGGGCAGGATCTCCTGTCATCTCACCTTGCTCCTGCCGAGAAAGTATCCATCATGGCTGAT

GCAATGCGGCGGCTGCATACGCTTGATCCGGCTACCTGCCCATTCGACCACCAAGCGAAACATCGCATCGAGCGAG

CACGTACTCGGATGGAAGCCGGTCTTGTCGATCAGGATGATCTGGACGAAGAGCATCAGGGGCTCGCGCCAGCCGA

ACTGTTCGCCAGGCTCAAGGCGCGCATGCCCGACGGCGATGATCTCGTCGTGACCCATGGCGATGCCTGCTTGCCG

AATATCATGGTGGAAAATGGCCGCTTTTCTGGATTCATCGACTGTGGCCGGCTGGGTGTGGCGGACCGCTATCAGG

ACATAGCGTTGGCTACCCGTGATATTGCTGAAGAGCTTGGCGGCGAATGGGCTGACCGCTTCCTCGTGCTTTACGG

TATCGCCGCTCCCGATTCGCAGCGCATCGCCTTCTATCGCCTTCTTGACGAGTTCTTC**TGA**GGGGATCAATTCTCT

AGAGCTCGCTGATCAGCCTCGACTGTGCCTTCTAGTTGCCAGCCATCTGTTGTTTGCCCCTCCCCCGTGCCTTCCT

TGACCCTGGAAGGTGCCACTCCCACTGTCCTTTCCTAATAAAATGAGGAAATTGCATCGCATTGTCTGAGTAGGTG

TCATTCTATTCTGGGGGGTGGGGTGGGGCAGGACAGCAAGGGGGAGGATTGGGAAGACAATAGCAGGCATGCTGGG

GATGCGGTGGGCTCTATGGCTTCTGAGGCGGAAAGAACCAGCTGGGGCTCGACTAGAGCTTGCGGAACCCTTCGAA
GTTCCTATTCTCTAGAAAGTATAGGAACTTCATCAGTCAGGTACATAAC**TAACTTTAAATAATTGGCATTATTTAA**
AGTTACTCGAGTATGGATGTGGCTAAATCCGTCTACCTTTCTGATGAGATTTGGGTATTATTTTTTCTGTCTCTGC
TGTTGGTTGGGTCTTTTGACACTGTGGGCTTTCTTTAAAGCCTCCTTCCTGCCATGTGGTCTCTTGTTTGCTACTA
ACTTCCCATGGCTTAAATGGCATGGCTTTTTGCCTTCTAAGGGCAGCTGCTGAGATTTGCAGCCTGATTTCCAGGG
TGGGGTTGGGAAATCTTTCAAACACTAAAATTGTCCTTTAATTTTTTTTTTAAAAAATGGGTTATATAATAAACCT
CATAAAATAGTTATGAGGAGTGAGGTGGACTAATATTAAATGAGTCCCTCCCCTATAAAAGAGCTATTAAGGCTTT
TTGTCTTATACTTAACTTTTTTTTTAAATGTGGTATCTTTAGAACCAAGGGTCTTAGAGTTTTAGTATACAGAAAC
TGTTGCATCGCTTAATCAGATTTTCTAGTTTCAAATCCAGAGAATCCAAATTCTTCACAGCCAAAGTCAAATTAAG
AATTTCTGACTTTTAATGTTAATTTGCTTACTGTGAATATAAAAATGATAGCTTTTCCTGAGGCAGGGTCTCACTA
TGTATCTCTGCCTGATCTGCAACAAGATATGTAGACTAAAGTTCTGCCTGCTTTTGTCTCCTGAATACTAAGGTTA
AAATGTAGTAATACTTTTGGAACTTGCAGGTCAGATTCTTTTATAGGGGACACACTAAGGGAGCTTGGGTGATAGT
TGGTAAAATGTGTTTCAAGTGATGAAAACTTGAATTATTATCACCGCAACCTACTTTTTAAAAAAAAAGCCAGGC
CTGTTAGAGCATGCTTAAGGGATCCCTAGGACTTGCTGAGCACACAAGAGTAGTTACTTGGCAGGCTCCTGGTGAG
AGCATATTTCAAAAAACAAGGCAGACAACCAAGAAACTACAGTTAAGGTTACCTGTCTTTAAACCATCTGCATATA
CACAGGGATATTAAAATATTCCAAATAATATTTCATTCAAGTTTTCCCCCATCAAATTGGGACATGGATTTCTCCG
GTGAATAGGCAGAGTTGGAAACTAAACAAATGTTGGTTTTGTGATTTGTGAAATTGTTTTCAAGTGATAGTTAAAG
CCCATGAGATACAGAACAAAGCTGCTATTTCGAGGTCTCTTGGTTTATACTCAGAAGCACTTCTTTGGGTTTCCCT
GCACTATCCTGATCATGTGCTAGGCCTACCTTAGGCTGATTGTTGTTCAAATAAACTTAAGTTTCCTGTCAGGTGA
TGTCATATGATTTCATATATCAAGGCAAAACATGTTATATATGTTAAACATTTGTACTTAATGTGAAAGTTAGGTC
TTTGTGGGTTTGATTTTTAATTTTCAAAACCTGAGCTAAATAAGTCATTTTTACATGTCTTACATTTGGTGGAATT
GTATAATTGTGGTTTGCAGGCAAGACTCTCTGACCTAGTAACCCTACCTATAGAGCACTTTGCTGGGTCACAAGTC
TAGGAGTCAAGCATTTCACCTTGAAGTTGAGACGTTTTGTTAGTGTATACTAGTTTATATGTTGGAGGACATGTTT
ATCCAGAAGATATTCAGGACTATTTTTGACTGGGCTAAGGAATTGATTCTGATTAGCACTGTTAGTGAGCATTGAG
TGGCCTTTAGGCTTGAATTGGAGTCACTTGTATATCTCAAATAATGCTGGCCTTTTTTAAAAAGCCCTTGTTCTTT
ATCACCCTGTTTTCTACATAATTTTTGTTCAAAGAAATACTTGTTTGGATCTCCTTTTGACAACAATAGCATGTTT
TCAAGCCATATTTTTTTTCCTTTTTTTTTTTTTTTTTGGTTTTTCGAGACAGGGTTTCTCTGTATAGCCCTGGCTG
TCCTGGAACTCACTTTGTAGACCAGGCTGGCCTCGAACTCAGAAATCCGCCTGCCTCTGCCTCCTGAGTGCCGGGA
TTAAAGGCGTGCACCACCACGCCTGGCTAAGTTGGATATTTTGTTATATAACTATAACCAATACTAACTCCACTGG
GTGGATTTTTAATTCAGTCAGTAGTCTTAAGTGGTCTTTATTGGCCCTTCATTAAAATCTACTGTTCACTCTAACA
GAGGCTGTTGGTACTAGTGGCACTTAAGCAACTTCCTACGGATATACTAGCAGATTAAGGGTCAGGGATAGAAACT
AGTCTAGCGTTTTGTATACCTACCAGCTTTATACTACCTTGTTCTGATAGAAATATTTCAGGACATCTAGAGTGTA
CTATAAGGTTGATGGTAAGCTTATAAGGAACTTGAAAGTGGAGTAACTACTCCATTTCTCTGAGGGGAGAATTAAA
ATTTTTGACCAAGTGTTGTTGAGCCACTGAGAATGGTCTCAGAACATAACTTCTTAAGGAACCTTCCCAGATTGCC
CTCAACACTGCACCACATTTGGTCCTGCTTGAACATTGCCATGGCTCTTAAAGTCTTAATTAAGAATATTAATTGT
GTAATTATTGTTTTTCCTCCTTTAGATCATTCCTTGAGGACAGGACAGTGCTTGTTTAAGGCTATATTTCTGCTGT
CTGAGCAGCAACAGGTCTTCGAGATCAACATGATGTTCATAATCCCAAGATGTTGCCATTTATGTTCTCAGAAGCA
AGCAGAGGCATGATGGTCAGTGACAGTAATGTCACTGTGTTAAATGTTGCTATGCAGTTTGGATTTTTCTAATGTA
GTGTAGGTAGAACATATGTGTTCTGTATGAATTAAACTCTTAAGTTACACCTTGTATAATCCATGCAATGTGTTAT
GCAATTACCATTTTAAGTATTGTAGCTTTCTTTGTATGTGAGGATAAAGGTGTTTGTCATAAAATGTTTTGAACAT
TTCCCCAAAGTTCCAAATTATAAAACCACAACGTTAGAACTTATTTATGAACAATGGTTGTAGTTTCATGCTTTTA
AAATGCTTAATTATTCAATTAACACCGTTTGTGTTATAATATATATAAAACTGACATGTAGAAGTGTTTGTCCAGA
ACATTTCTTAAATGTATACTGTCTTTAGAGAGTTTAATATAGCATGTCTTTTGCAACATACTAACTTTTGTGTTGG
TGCGAGCAATATTGTGTAGTCATTTTGAAAGGAGTCATTTCAATGAGTGTCAGATTGTTTTGAATGTTATTGAACA

TTTTAAATGCAGACTTGTTCGTGTTTAGAAAGCAAAACTGTCAGAAGCTTTGAACTAGAAATTAAAAAGCTGAAG
TATTTCAGAAGGGAAATAAGCTACTTGCTGTATTAGTTGAAGGAAAGTGTAATAGCTTAGAAAATTTAAAACCATA
TAGTTGTCATTGCTGAATATCTGGCAGATGAAAAGAAATACTCAGTGGTTCTTTTGAGCAATATAACAGCTTGTTA
TATTAAAAATTTTCCCCACAGATATAAACTCTAATCTATAACTCATAAATGTTACAAATGGATGAAGCTTACAAAT
GTGGCTTGACTTGTCACTGTGCTTGTTTTAGTTATGTGAAAGTTTGGCAATAAACCTATGTCCTAAATAGTCAAAC
TGTGGAATGACTTTTTAATCTATTGGTTTGTCTAGAACAGTTATGTTGCCATTTGCCCTAATGGTGAAAGAAAAAG
TGGGGAGTGCCTTGGCACTGTTCATTTGTGGTGTGAACCAAAGAGGGGGGCATGCACTTACACTTCAAACATCCTT
TTGAAAGACTGACAAGTTTGGGTCTTCACAGTTGGAATTGGGCATCCCTTTTGTCAGGGAGGGAGGGAGGGAGGGA
GGCTGGCTTGTTATGCTGACAAGTGTGATTAAATTCAAACTTTGAGGTAAGTTGGAGGAACTTGTACATTGTTAGG
AGTGTGACAATTTGGACTCTTAATGATTTGGTCATACAAAATGAACCTAGACCAACTTCTGGAAGATGTATATAAT
AACTCCATGTTACATTGATTTCACCTGACTAATACTTATCCCTTATCAATTAAATACAGAAGATGCCAGCCATCTG
GGCCTTTTAACCCAGAAATTTAGTTTCAAACTCCTAGGTTAGTGTTCTCACTGAGCTACATCCTGATCTAGTCCTG
AAAATAGGACCACCGGCGCGCCACTCTTCGCGACAGCTAGATCTCATCGCCTAGGATCGCCCGGGTTGATTCGAGG
CTGCTAACAAATCGAGCAGTGTGGTTTTCAAGAGGAAGCAAAAAGCCTCTCCACCCAGGCCTGGAATGTTTCCACC
CAATGTCGAGCAGTGTGGTTTTGCAAGAGGAAGCAAAAAGCCTCTCCACCCAGGCCTGGAATGTTTCCACCCAATG
TCGAGCAAACCCCGCCCAGCGTCTTGTCATTGGCGAATTCGAACACGCAGATGCAGTCGGGGCGGCGCGGTCCGAG
GTCCACTTCGCATATTAAGGTGACGCGTGTGGCCTCGAACACCGAGCGACCCTGCAGCGACCCGCTTAACAGCGTC
AACAGCGTGCCGCAGATCTTGGTGGCGTGAAACTCCCGCACCTCTTCGGCCAGCGCCTTGTAGAAGCGCGTGCCAT
GGATCCTGATGATGTTGTTGATTCTTCTAAATCTTTTGTGATGGAAAACTTTTCTTCGTACCACGGGACTAAACCT
GGTTATGTAGATTCCATTCAAAAAGGTATACAAAAGCCAAAATCTGGTACACAAGGAAATTATGACGATGATTGGA
AAGGGTTTTATAGTACCGACAATAAATACGACGCTGCGGGATACTCTGTAGATAATGAAAACCCGCTCTCTGGAAA
AGCTGGAGGCGTGGTCAAAGTGACGTATCCAGGACTGACGAAGGTTCTCGCACTAAAAGTGGATAATGCCGAAACT
ATTAAGAAAGAGTTAGGTTTAAGTCTCACTGAACCGTTGATGGAGCAAGTCGGAACGGAAGAGTTTATCAAAAGGT
TCGGTGATGGTGCTTCGCGTGTAGTGCTCAGCCTTCCCTTCGCTGAGGGGAGTTCTAGCGTTGAATATATTAATAA
CTGGGAACAGGCGAAAGCGTTAAGCGTAGAACTTGAGATTAATTTTGAAACCCGTGGAAAACGTGGCCAAGATGCG
ATGTATGAGTATATGGCTCAAGCCTGTGCAGGAAATCGTGTCAGGCGATCTCTTTGTGAAGGAACCTTACTTCTGT
GGTGTGACATAATTGGACAAACTACCTACAGAGATTTAAAGCTCTAAGGTAAATATAAAATTTTTAAGTGTATAAT
GTGTTAAACTACTGATTCTAATTGTTTGTGTATTTTAGATTCCAACCTATGGAACTGATGAATGGGAGCAGTGGTG
GAATGCAGATCCTAGAGCTCGCTGATCAGCCTCGACTGTGCCTTCTAGTTGCCAGCCATCTGTTGTTTGCCCCTCC
CCCGTGCCTTCCTTGACCCTGGAAGGTGCCACTCCCACTGTCCTTTCCTAATAAAATGAGGAAATTGCATCGCATT
GTCTGAGTAGGTGTCATTCTATTCTGGGGGGTGGGGTGGGGCAGGACAGCAAGGGGGAGGATTGGGAAGACAATAG
CAGGCATGCTGGGGATGCGGTGGGCTCTATGGCTTCTGAGGCGGAAAGAACCAGCCCGGGCGGTGGAGCTCCAATT
CGCCCTATAGTGAGTCGTATTACAATTCACTGGCCGTCGTTTTACAACGTCGTGACTGGGAAAACCCTGGCGTTAC
CCAACTTAATCGCCTTGCAGCACATCCCCCTTTCGCCAGCTGGCGTAATAGCGAAGAGGCCCGCACCGATCGCCCT
TCCCAACAGTTGCGCAGCCTGAATGGCGAATGGAAATTGTAAGCG

Supplementary Document 2. Key features and DNA sequence of the *EF1α-loxP-Stop-loxP-mTert*$^{flox/flox}$ allele after *Neo* deletion (6557 bp).

Location (bp)	Feature	Size (bp)	Label	
			Highlight	Font color
1–300	*Rosa26 5'Arm*	300	Green	
301–1478	Human *EF1α* promoter	1178	Yellow	
1506–1540	*LoxP*	35		Red/Bold
1541–2382	*STOP*	842	Grey	

2383–2417	*LoxP*	35		Red/Bold
2455–5823	Mouse *Tert* cDNA	3369	Cyan	
5824–6078	*HSV-TK* poly (A) signal	255	Grey	
6142–6173	*Rox* site	32		Purple/Bold
6180–6557	*Rosa26 3'Arm*	378	Green	

ATCTATACAAAGCTGAAAGCTAAGTCTGCAGCCATTACTAAACATGAAAGCAAGTAATGATAATTTTGGATTTCAA
AAATGTAGGGCCAGAGTTTAGCCAGCCAGTGGTGGTGCTTGCCTTTATGCCTTTAATCCCAGCACTCTGGAGGCAG
AGACAGGCAGATCTCTGAGTTTGAGCCCAGCCTGGTCTACACATCAAGTTCTATCTAGGATAGCCAGGAATACACA
CAGAAACCCTGTTGGGGAGGGGGGCTCTGAGATTTCATAAAATTATAATTGAAGCATTCCCTAATGAGCCACGTCG
ACGGCTCCGGTGCCCGTCAGTGGGCAGAGCGCACATCGCCCACAGTCCCCGAGAAGTTGGGGGGAGGGGTCGGCAA
TTGAACCGGTGCCTAGAGAAGGTGGCGCGGGGTAAACTGGGAAAGTGATGTCGTGTACTGGCTCCGCCTTTTTCCC
GAGGGTGGGGGAGAACCGTATATAAGTGCAGTAGTCGCCGTGAACGTTCTTTTTCGCAACGGGTTTGCCGCCAGAA
CACAGGTAAGTGCCGTGTGTGGTTCCCGCGGGCCTGGCCTCTTTACGGGTTATGGCCCTTGCGTGCCTTGAATTAC
TTCCACCTGGCTGCAGTACGTGATTCTTGATCCCGAGCTTCGGGTTGGAAGTGGGTGGGAGAGTTCGAGGCCTTGC
GCTTAAGGAGCCCCTTCGCCTCGTGCTTGAGTTGAGGCCTGGCCTGGGCGCTGGGGCCGCCGCGTGCGAATCTGGT
GGCACCTTCGCGCCTGTCTCGCTGCTTTCGATAAGTCTCTAGCCATTTAAAATTTTTGATGACCTGCTGCGACGCT
TTTTTTCTGGCAAGATAGTCTTGTAAATGCGGGCCAAGATCTGCACACTGGTATTTCGGTTTTTGGGGCCGCGGGC
GGCGACGGGGCCCGTGCGTCCCAGCGCACATGTTCGGCGAGGCGGGGCCTGCGAGCGCGGCCACCGAGAATCGGAC
GGGGGTAGTCTCAAGCTGGCCGGCCTGCTCTGGTGCCTGGTCTCGCGCCGCCGTGTATCGCCCCGCCCTGGGCGGC
AAGGCTGGCCCGGTCGGCACCAGTTGCGTGAGCGGAAAGATGGCCGCTTCCCGGCCCTGCTGCAGGGAGCTCAAAA
TGGAGGACGCGGCGCTCGGGAGAGCGGGCGGGTGAGTCACCCACACAAAGGAAAAGGGCCTTTCCGTCCTCAGCCG
TCGCTTCATGTGACTCCACGGAGTACCGGGCGCCGTCCAGGCACCTCGATTAGTTCTCGAGCTTTTGGAGTACGTC
GTCTTTAGGTTGGGGGGAGGGGTTTTATGCGATGGAGTTTCCCCACACTGAGTGGGTGGAGACTGAAGTTAGGCCA
GCTTGGCACTTGATGTAATTCTCCTTGGAATTTGCCCTTTTTGAGTTTGGATCTTGGTTCATTCTCAAGCCTCAGA
CAGTGGTTCAAAGTTTTTTTCTTCCATTTCAGGTGTCGTGAGGCTGCAGGTCGAGGGACCTAATAACTTCGTATAG
CATACATTATACGAAGTTATATTAAGGGTTCCGGATCAGCTTGATGGGGATCCAGACATGATAAGATACATTGATG
AGTTTGGACAAACCACAACTAGAATGCAGTGAAAAAAATGCTTTATTTGTGAAATTTGTGATGCTATTGCTTTATT
TGTAACCATTATAAGCTGCAATAAACAAGTTAACAACAACAATTGCATTCATTTTATGTTTCAGGTTCAGGGGGAG
GTGTGGGAGGTTTTTTAAAGCAAGTAAAACCTCTACAAATGTGGTATGGCTGATTATGATCCTCTAGAGTCGCAGA
TCCAGACATGATAAGATACATTGATGAGTTTGGACAAACCACAACTAGAATGCAGTGAAAAAAATGCTTTATTTGT
GAAATTTGTGATGCTATTGCTTTATTTGTAACCATTATAAGCTGCAATAAACAAGTTAACAACAACAATTGCATTC
ATTTTATGTTTCAGGTTCAGGGGGAGGTGTGGGAGGTTTTTTAAAGCAAGTAAAACCTCTACAAATGTGGTATGGC
TGATTATGATCCTCTAGAGTCGCAGATCCAGACATGATAAGATACATTGATGAGTTTGGACAAACCACAACTAGAA
TGCAGTGAAAAAAATGCTTTATTTGTGAAATTTGTGATGCTATTGCTTTATTTGTAACCATTATAAGCTGCAATAA
ACAAGTTAACAACAACAATTGCATTCATTTTATGTTTCAGGTTCAGGGGGAGGTGTGGGAGGTTTTTTAAAGCAAG
TAAAACCTCTACAAATGTGGTATGGCTGATTATGATCCTCTAGAGTCGCAGATCCTCTAGAGTCGCAGATCTGCAA
GCTAATTCCTGCAGGTCGAGGGACCTAATAACTTCGTATAGCATACATTATACGAAGTTATATTAAGGGTTCCGGA
TCCACTACACCACGTGGCCACCATGACCCGCGCTCCTCGTTGCCCCGCGGTGCGCTCTCTGCTGCGCAGCCGATAC
CGGGAGGTGTGGCCGCTGGCAACCTTTGTGCGGCGCCTGGGGCCCGAGGGCAGGCGGCTTGTGCAACCCGGGGACC
CGAAGATCTACCGCACTTTGGTTGCCCAATGCCTAGTGTGCATGCACTGGGGCTCACAGCCTCCACCTGCCGACCT
TTCCTTCCACCAGGTGTCATCCCTGAAAGAGCTGGTGGCCAGGGTTGTGCAGAGACTCTGCGAGCGCAACGAGAGA
AACGTGCTGGCTTTTGGCTTTGAGCTGCTTAACGAGGCCAGAGGCGGGCCTCCCATGGCCTTCACTAGTAGCGTGC
GTAGCTACTTGCCCAACACTGTTATTGAGACCCTGCGTGTCAGTGGTGCATGGATGCTACTGTTGAGCCGAGTGGG
CGACGACCTGCTGGTCTACCTGCTGGCACACTGTGCTCTTTATCTTCTGGTGCCCCCCAGCTGTGCCTACCAGGTG

TGTGGGTCTCCCCTGTACCAAATTTGTGCCACCACGGATATCTGGCCCTCTGTGTCCGCTAGTTACAGGCCCACCC
GACCCGTGGGCAGGAATTTCACTAACCTTAGGTTCTTACAACAGATCAAGAGCAGTAGTCGCCAGGAAGCACCGAA
ACCCCTGGCCTTGCCATCTCGAGGTACAAAGAGGCATCTGAGTCTCACCAGTACAAGTGTGCCTTCAGCTAAGAAG
GCCAGATGCTATCCTGTCCCGAGAGTGGAGGAGGGACCCCACAGGCAGGTGCTACCAACCCCATCAGGCAAATCAT
GGGTGCCAAGTCCTGCTCGGTCCCCCGAGGTGCCTACTGCAGAGAAAGATTTGTCTTCTAAAGGAAAGGTGTCTGA
CCTGAGTCTCTCTGGGTCGGTGTGCTGTAAACACAAGCCCAGCTCCACATCTCTGCTGTCACCACCCCGCCAAAAT
GCCTTTCAGCTCAGGCCATTTATTGAGACCAGACATTTCCTTTACTCCAGGGGAGATGGCCAAGAGCGTCTAAACC
CCTCATTCCTACTCAGCAACCTCCAGCCTAACTTGACTGGGGCCAGGAGACTGGTGGAGATCATCTTTCTGGGCTC
AAGGCCTAGGACATCAGGACCACTCTGCAGGACACACCGTCTATCGCGTCGATACTGGCAGATGCGGCCCCTGTTC
CAACAGCTGCTGGTGAACCATGCAGAGTGCCAATATGTCAGACTCCTCAGGTCACATTGCAGGTTTCGAACAGCAA
ACCAACAGGTGACAGATGCCTTGAACACCAGCCCACCGCACCTCATGGATTTGCTCCGCCTGCACAGCAGTCCCTG
GCAGGTATATGGTTTTCTTCGGGCCTGTCTCTGCAAGGTGGTGTCTGCTAGTCTCTGGGGTACCAGGCACAATGAG
CGCCGCTTCTTTAAGAACTTAAAGAAGTTCATCTCGTTGGGGAAATACGGCAAGCTATCACTGCAGGAACTGATGT
GGAAGATGAAAGTAGAGGATTGCCACTGGCTCCGCAGCAGCCCGGGGAAGGACCGTGTCCCCGCTGCAGAGCACCG
TCTGAGGGAGAGGATCCTGGCTACGTTCCTGTTCTGGCTGATGGACACATACGTGGTACAGCTGCTTAGGTCATTC
TTTTACATCACAGAGAGCACATTCCAGAAGAACAGGCTCTTCTTCTACCGTAAGAGTGTGTGGAGCAAGCTGCAGA
GCATTGGAGTCAGGCAACACCTTGAGAGAGTGCGGCTACGGGAGCTGTCACAAGAGGAGGTCAGGCATCACCAGGA
CACCTGGCTAGCCATGCCCATCTGCAGACTGCGCTTCATCCCCAAGCCCAACGGCCTGCGGCCCATTGTGAACATG
AGTTATAGCATGGGTACCAGAGCTTTGGGCAGAAGGAAGCAGGCCCAGCATTTCACCCAGCGTCTCAAGACTCTCT
TCAGCATGCTCAACTATGAGCGGACAAAACATCCTCACCTTATGGGGTCTTCTGTACTGGGTATGAATGACATCTA
CAGGACCTGGCGGGCCTTTGTGCTGCGTGTGCGTGCTCTGGACCAGACACCCAGGATGTACTTTGTTAAGGCAGAT
GTGACCGGGGCCTATGATGCCATCCCCCAGGGTAAGCTGGTGGAGGTTGTTGCCAATATGATCAGGCACTCGGAGA
GCACGTACTGTATCCGCCAGTATGCAGTGGTCCGGAGAGATAGCCAAGGCCAAGTCCACAAGTCCTTTAGGAGACA
GGTCACCACCCTCTCTGACCTCCAGCCATACATGGGCCAGTTCCTTAAGCATCTGCAGGATTCAGATGCCAGTGCA
CTGAGGAACTCCGTTGTCATCGAGCAGAGCATCTCTATGAATGAGAGCAGCAGCCTGTTTGACTTCTTCCTGC
ACTTCCTGCGTCACAGTGTCGTAAAGATTGGTGACAGGTGCTATACGCAGTGCCAGGGCATCCCCCAGGGCTCCAG
CCTATCCACCCTGCTCTGCAGTCTGTGTTTCGGAGACATGGAGAACAAGCTGTTTGCTGAGGTGCAGCGGGATGGG
TTGCTTTTACGTTTTGTTGATGACTTTCTGTTGGTGACGCCTCACTTGGACCAAGCAAAAACCTTCCTCAGCACCC
TGGTCCATGGCGTTCCTGAGTATGGGTGCATGATAAACTTGCAGAAGACAGTGGTGAACTTCCCTGTGGAGCCTGG
TACCCTGGGTGGTGCAGCTCCATACCAGCTGCCTGCTCACTGCCTGTTTCCCTGGTGTGGCTTGCTGCTGGACACT
CAGACTTTGGAGGTGTTCTGTGACTACTCAGGTTATGCCCAGACCTCAATTAAGACGAGCCTCACCTTCCAGAGTG
TCTTCAAAGCTGGGAAGACCATGCGGAACAAGCTCCTGTCGGTCTTGCGGTTGAAGTGTCACGGTCTATTTCTAGA
CTTGCAGGTGAACAGCCTCCAGACAGTCTGCATCAATATATACAAGATCTTCCTGCTTCAGGCCTACAGGTTCCAT
GCATGTGTGATTCAGCTTCCCTTTGACCAGCGTGTTAGGAAGAACCTCACATTCTTTCTGGGCATCATCTCCAGCC
AAGCATCCTGCTGCTATGCTATCCTGAAGGTCAAGAATCCAGGAATGACACTAAAGGCCTCTGGCTCCTTTCCTCC
TGAAGCCGCACATTGGCTCTGCTACCAGGCCTTCCTGCTCAAGCTGGCTGCTCATTCTGTCATCTACAAATGTCTC
CTGGGACCTCTGAGGACAGCCCAAAAACTGCTGTGCCGGAAGCTCCCAGAGGCGACAATGACCATCCTTAAAGCTG
CAGCTGACCCAGCCCTAAGCACAGACTTTCAGACCATTTTGGAC**TAA**GCGATCGCACCCGCGGACTAGAGCTGGGG
GAGGCTAACTGAAACACGGAAGGAGACAATACCGGAAGGAACCCGCGCTATGACGGCAATAAAAAGACAGAATAAA
ACGCACGGGTGTTGGGTCGTTTGTTCATAAACGCGGGGTTCGGTCCCAGGGCTGGCACTCTGTCGATACCCCACCG
AGACCCCATTGGGGCCAATACGCCCGCGTTTCTTCCTTTTCCCCACCCCACCCCCCAAGTTCGGGTGAAGGCCCAG
GGCTCGCAGCCAACGTCGGGGCGGCAGGCCCTGCCATAGCGCATTCTGGTGTCGACGCTAC**TAACTTTAAATAATT**
GGCATTATTTAAAGTTACTCGAGTATGGATGTGGCTAAATCCGTCTACCTTTCTGATGAGATTTGGGTATTATTTT
TTCTGTCTCTGCTGTTGGTTGGGTCTTTTGACACTGTGGGCTTTCTTTAAAGCCTCCTTCCTGCCATGTGGTCTCT

TGTTTGCTACTAACTTCCCATGGCTTAAATGGCATGGCTTTTTGCCTTCTAAGGGCAGCTGCTGAGATTTGCAGCC
TGATTTCCAGGGTGGGGTTGGGAAATCTTTCAAACACTAAAATTGTCCTTTAATTTTTTTTTAAAAAATGGGTTA
TATAATAAACCTCATAAAATAGTTATGAGGAGTGAGGTGGACTAATATTAAATGAGTCCCTCCCCTATAAAAGAGC
TATTAAGGCTTTTTGTCTTAT

Supplementary Document 3. Key features and DNA sequence of the *EF1α-mTert*$^{flox/flox}$ allele after *Stop* deletion (5548 bp).

Location (bp)	Feature	Size (bp)	Label	
			Highlight	Font color
1–300	*Rosa26 5'Arm* (*5'* integration site)	300	Green	
301–1478	Human *EF1α* promoter	1178	Yellow	
1506–1540	*LoxP*	35		Red/Bold
1578–4946	Mouse *Tert* cDNA	3369	Cyan	
4947–5201	*HSV-TK* poly (A) signal	255	Grey	
5265–5296	*Rox* site	32		Purple/Bold
5303–5548	*Rosa26 3'Arm* (*3'* integration site)	246	Green	

ATCTATACAAAGCTGAAAGCTAAGTCTGCAGCCATTACTAAACATGAAAGCAAGTAATGATAATTTTGGATTTCAA
AAATGTAGGGCCAGAGTTTAGCCAGCCAGTGGTGGTGCTTGCCTTTATGCCTTTAATCCCAGCACTCTGGAGGCAG
AGACAGGCAGATCTCTGAGTTTGAGCCCAGCCTGGTCTACACATCAAGTTCTATCTAGGATAGCCAGGAATACACA
CAGAAACCCTGTTGGGGAGGGGGGCTCTGAGATTTCATAAAATTATAATTGAAGCATTCCCTAATGAGCCAC**GTCG**
ACGGCTCCGGTGCCCGTCAGTGGGCAGAGCGCACATCGCCCACAGTCCCCGAGAAGTTGGGGGGAGGGGTCGGCAA
TTGAACCGGTGCCTAGAGAAGGTGGCGCGGGGTAAACTGGGAAAGTGATGTCGTGTACTGGCTCCGCCTTTTTCCC
GAGGGTGGGGGAGAACCGTATATAAGTGCAGTAGTCGCCGTGAACGTTCTTTTTCGCAACGGGTTTGCCGCCAGAA
CACAGGTAAGTGCCGTGTGTGGTTCCCGCGGGCCTGGCCTCTTTACGGGTTATGGCCCTTGCGTGCCTTGAATTAC
TTCCACCTGGCTGCAGTACGTGATTCTTGATCCCGAGCTTCGGGTTGGAAGTGGGTGGGAGAGTTCGAGGCCTTGC
GCTTAAGGAGCCCCTTCGCCTCGTGCTTGAGTTGAGGCCTGGCCTGGGCGCTGGGGCCGCCGCGTGCGAATCTGGT
GGCACCTTCGCGCCTGTCTCGCTGCTTTCGATAAGTCTCTAGCCATTTAAAATTTTTGATGACCTGCTGCGACGCT
TTTTTTCTGGCAAGATAGTCTTGTAAATGCGGGCCAAGATCTGCACACTGGTATTTCGGTTTTTGGGGCCGCGGGC
GGCGACGGGGCCCGTGCGTCCCAGCGCACATGTTCGGCGAGGCGGGGCCTGCGAGCGCGGCCACCGAGAATCGGAC
GGGGGTAGTCTCAAGCTGGCCGGCCTGCTCTGGTGCCTGGTCTCGCGCCGCCGTGTATCGCCCCGCCCTGGGCGGC
AAGGCTGGCCCGGTCGGCACCAGTTGCGTGAGCGGAAAGATGGCCGCTTCCCGGCCCTGCTGCAGGGAGCTCAAAA
TGGAGGACGCGGCGCTCGGGAGAGCGGGCGGGTGAGTCACCCACACAAAGGAAAAGGGCCTTTCCGTCCTCAGCCG
TCGCTTCATGTGACTCCACGGAGTACCGGGCGCCGTCCAGGCACCTCGATTAGTTCTCGAGCTTTTGGAGTACGTC
GTCTTTAGGTTGGGGGGAGGGGTTTTATGCGATGGAGTTTCCCCACACTGAGTGGGTGGAGACTGAAGTTAGGCCA
GCTTGGCACTTGATGTAATTCTCCTTGGAATTTGCCCTTTTTGAGTTTGGATCTTGGTTCATTCTCAAGCCTCAGA
CAGTGGTTCAAAGTTTTTTTCTTCCATTTCAGGTGTCGTGAGGCTGCAGGTCGAGGGACCT**AATAACTTCGTATAG**
CATACATTATACGAAGTTATATTAAGGGTTCCGGATCCACTACACCACGTGGCCACC**ATG**ACCCGCGCTCCTCGTT
GCCCCGCGGTGCGCTCTCTGCTGCGCAGCCGATACCGGGAGGTGTGGCCGCTGGCAACCTTTGTGCGGCGCCTGGG
GCCCGAGGGCAGGCGGCTTGTGCAACCCGGGGACCCGAAGATCTACCGCACTTTGGTTGCCCAATGCCTAGTGTGC
ATGCACTGGGGCTCACAGCCTCCACCTGCCGACCTTTCCTTCCACCAGGTGTCATCCCTGAAAGAGCTGGTGGCCA
GGGTTGTGCAGAGACTCTGCGAGCGCAACGAGAGAAACGTGCTGGCTTTTGGCTTTGAGCTGCTTAACGAGGCCAG
AGGCGGGCCTCCCATGGCCTTCACTAGTAGCGTGCGTAGCTACTTGCCCAACACTGTTATTGAGACCCTGCGTGTC
AGTGGTGCATGGATGCTACTGTTGAGCCGAGTGGGCGACGACCTGCTGGTCTACCTGCTGGCACACTGTGCTCTTT
ATCTTCTGGTGCCCCCCAGCTGTGCCTACCAGGTGTGTGGGTCTCCCCTGTACCAAATTTGTGCCACCACGGATAT

CTGGCCCTCTGTGTCCGCTAGTTACAGGCCCACCCGACCCGTGGGCAGGAATTTCACTAACCTTAGGTTCTTACAA
CAGATCAAGAGCAGTAGTCGCCAGGAAGCACCGAAACCCCTGGCCTTGCCATCTCGAGGTACAAAGAGGCATCTGA
GTCTCACCAGTACAAGTGTGCCTTCAGCTAAGAAGGCCAGATGCTATCCTGTCCCGAGAGTGGAGGAGGGACCCCA
CAGGCAGGTGCTACCAACCCCATCAGGCAAATCATGGGTGCCAAGTCCTGCTCGGTCCCCCGAGGTGCCTACTGCA
GAGAAAGATTTGTCTTCTAAAGGAAAGGTGTCTGACCTGAGTCTCTCTGGGTCGGTGTGCTGTAAACACAAGCCCA
GCTCCACATCTCTGCTGTCACCACCCCGCCAAAATGCCTTTCAGCTCAGGCCATTTATTGAGACCAGACATTTCCT
TTACTCCAGGGGAGATGGCCAAGAGCGTCTAAACCCCTCATTCCTACTCAGCAACCTCCAGCCTAACTTGACTGGG
GCCAGGAGACTGGTGGAGATCATCTTTCTGGGCTCAAGGCCTAGGACATCAGGACCACTCTGCAGGACACACCGTC
TATCGCGTCGATACTGGCAGATGCGGCCCCTGTTCCAACAGCTGCTGGTGAACCATGCAGAGTGCCAATATGTCAG
ACTCCTCAGGTCACATTGCAGGTTTCGAACAGCAAACCAACAGGTGACAGATGCCTTGAACACCAGCCCACCGCAC
CTCATGGATTTGCTCCGCCTGCACAGCAGTCCCTGGCAGGTATATGGTTTTCTTCGGGCCTGTCTCTGCAAGGTGG
TGTCTGCTAGTCTCTGGGGTACCAGGCACAATGAGCGCCGCTTCTTTAAGAACTTAAAGAAGTTCATCTCGTTGGG
GAAATACGGCAAGCTATCACTGCAGGAACTGATGTGGAAGATGAAAGTAGAGGATTGCCACTGGCTCCGCAGCAGC
CCGGGGAAGGACCGTGTCCCCGCTGCAGAGCACCGTCTGAGGGAGAGGATCCTGGCTACGTTCCTGTTCTGGCTGA
TGGACACATACGTGGTACAGCTGCTTAGGTCATTCTTTTACATCACAGAGAGCACATTCCAGAAGAACAGGCTCTT
CTTCTACCGTAAGAGTGTGTGGAGCAAGCTGCAGAGCATTGGAGTCAGGCAACACCTTGAGAGAGTGCGGCTACGG
GAGCTGTCACAAGAGGAGGTCAGGCATCACCAGGACACCTGGCTAGCCATGCCCATCTGCAGACTGCGCTTCATCC
CCAAGCCCAACGGCCTGCGGCCCATTGTGAACATGAGTTATAGCATGGGTACCAGAGCTTTGGGCAGAAGGAAGCA
GGCCCAGCATTTCACCCAGCGTCTCAAGACTCTCTTCAGCATGCTCAACTATGAGCGGACAAAACATCCTCACCTT
ATGGGGTCTTCTGTACTGGGTATGAATGACATCTACAGGACCTGGCGGGCCTTTGTGCTGCGTGTGCGTGCTCTGG
ACCAGACACCCAGGATGTACTTTGTTAAGGCAGATGTGACCGGGGCCTATGATGCCATCCCCCAGGGTAAGCTGGT
GGAGGTTGTTGCCAATATGATCAGGCACTCGGAGAGCACGTACTGTATCCGCCAGTATGCAGTGGTCCGGAGAGAT
AGCCAAGGCCAAGTCCACAAGTCCTTTAGGAGACAGGTCACCACCCTCTCTGACCTCCAGCCATACATGGGCCAGT
TCCTTAAGCATCTGCAGGATTCAGATGCCAGTGCACTGAGGAACTCCGTTGTCATCGAGCAGAGCATCTCTATGAA
TGAGAGCAGCAGCAGCCTGTTTGACTTCTTCCTGCACTTCCTGCGTCACAGTGTCGTAAAGATTGGTGACAGGTGC
TATACGCAGTGCCAGGGCATCCCCCAGGGCTCCAGCCTATCCACCCTGCTCTGCAGTCTGTGTTTCGGAGACATGG
AGAACAAGCTGTTTGCTGAGGTGCAGCGGGATGGGTTGCTTTTACGTTTTGTTGATGACTTTCTGTTGGTGACGCC
TCACTTGGACCAAGCAAAAACCTTCCTCAGCACCCTGGTCCATGGCGTTCCTGAGTATGGGTGCATGATAAACTTG
CAGAAGACAGTGGTGAACTTCCCTGTGGAGCCTGGTACCCTGGGTGGTGCAGCTCCATACCAGCTGCCTGCTCACT
GCCTGTTTCCCTGGTGTGGCTTGCTGCTGGACACTCAGACTTTGGAGGTGTTCTGTGACTACTCAGGTTATGCCCA
GACCTCAATTAAGACGAGCCTCACCTTCCAGAGTGTCTTCAAAGCTGGGAAGACCATGCGGAACAAGCTCCTGTCG
GTCTTGCGGTTGAAGTGTCACGGTCTATTTCTAGACTTGCAGGTGAACAGCCTCCAGACAGTCTGCATCAATATAT
ACAAGATCTTCCTGCTTCAGGCCTACAGGTTCCATGCATGTGTGATTCAGCTTCCCTTTGACCAGCGTGTTAGGAA
GAACCTCACATTCTTTCTGGGCATCATCTCCAGCCAAGCATCCTGCTGCTATGCTATCCTGAAGGTCAAGAATCCA
GGAATGACACTAAAGGCCTCTGGCTCCTTTCCTCCTGAAGCCGCACATTGGCTCTGCTACCAGGCCTTCCTGCTCA
AGCTGGCTGCTCATTCTGTCATCTACAAATGTCTCCTGGGACCTCTGAGGACAGCCCAAAAACTGCTGTGCCGGAA
GCTCCCAGAGGCGACAATGACCATCCTTAAAGCTGCAGCTGACCCAGCCCTAAGCACAGACTTTCAGACCATTTTG
GAC**TAA**GCGATCGCACCCGCGGACTAGAGCTGGGGGAGGCTAACTGAAACACGGAAGGAGACAATACCGGAAGGAA
CCCGCGCTATGACGGCAATAAAAAGACAGAATAAAACGCACGGGTGTTGGGTCGTTTGTTCATAAACGCGGGGTTC
GGTCCCAGGGCTGGCACTCTGTCGATACCCCACCGAGACCCCATTGGGGCCAATACGCCCGCGTTTCTTCCTTTTC
CCCACCCCACCCCCCAAGTTCGGGTGAAGGCCCAGGGCTCGCAGCCAACGTCGGGGCGGCAGGCCCTGCCATAGCG
CATTCTGGTGTCGACGCTAC**TAACTTTAAATAATTGGCATTATTTAAAGTTA**CTCGAGTATGGATGTGGCTAAATC
CGTCTACCTTTCTGATGAGATTTGGGTATTATTTTTCTGTCTCTGCTGTTGGTTGGGTCTTTTGACACTGTGGGC
TTTCTTTAAAGCCTCCTTCCTGCCATGTGGTCTCTTGTTTGCTACTAACTTCCCATGGCTTAAATGGCATGGCTTT

TTGCCTTCTAAGGGCAGCTGCTGAGATTTGCAGCCTGATTTCCAGGGTGGGGTTGGGAAATCTTTCAAACACTAAA
