# Supplementary material for: Telomerase reverse transcriptase gene knock‐in unleashes enhanced longevity and accelerated damage repair in mice
Source: Aging Cell. 2024 Dec 11;24(4):e14445. doi: 10.1111/acel.14445 (PMC11984681; doi:10.1111/acel.14445)
Supplement: Supplementary file 2 — Tables S1‐S4. [file ACEL-24-e14445-s001.docx]

**Supplementary Tables**

**Supplementary Table 1. Blood routine test comparison between WT (C57BL/6) and *TertKI* (G4) mice.**

(Both over 8 weeks old, *n* = 3)

| **Test item** | **Abbr.** | **Ref. range** | **Units** | **WT mice** | | | ***TertKI* mice** | | | **Diff.** |
| --- | --- | --- | --- | --- | --- | --- | --- | --- | --- | --- |
|  |  |  |  | **Mean** | **SEM** | ***p*** | **Mean** | **SEM** | ***p*** |  |
| White Blood Cells | **WBC** | 0.8–6.8 | 10^9^/L | 3.47 | 0.81 | 1 | 6.08 | 1.11 | 0.0189 | *****^b^ |
| LYMphocyte ratio | **LYM%** | 55.8–90.6 | % | 47.77↓^a^ | 19.42 | 1 | 47.60↓ | 3.36 | 0.9868 | NS |
| neutrophilic GRANulocyte ratio | **GRAN%** | 8.6–38.9 | % | 45.37↑ | 18.77 | 1 | 40.48↑ | 4.03 | 0.6241 | NS |
| LYMphocyte | **LYM** | 0.7–5.7 | 10^9^/L | 1.53 | 0.23 | 1 | 2.90 | 0.63 | 0.0173 | ***** |
| Red Blood Cells | **RBC** | 6.36–9.42 | 10^12^/L | 5.74↓ | 0.87 | 1 | 6.54 | 0.42 | 0.1595 | NS |
| HemoGloBin | **HGB** | 110–143 | g/L | 92.67↓ | 14.47 | 1 | 115.75 | 4.86 | 0.0283 | ***** |
| HematoCriT | **HCT** | 34.6–44.6 | % | 30.10↓ | 5.24 | 1 | 32.58↓ | 2.37 | 0.4315 | NS |
| Mean Corpuscular Volume | **MCV** | 38.2–58.3 | fL | 52.57 | 1.93 | 1 | 49.85 | 0.72 | 0.0458 | ***** |
| Mean Corpuscular Hemoglobin | **MCH** | 15.8–19.0 | pg | 16.11 | 0.11 | 1 | 17.68 | 0.88 | 0.0303 | ***** |
| Red blood cell Distribution Width-Standard Deviation | **RDW-SD** | 0.1–99.9 | fL | 24.13 | 1.85 | 1 | 21.83 | 0.95 | 0.0805 | NS |
| Red cell Distribution Width-Coefficient of Variation | **RDW-CV** | 13.0–17.0 | % | 14.40 | 0.70 | 1 | 13.65 | 0.45 | 0.1421 | NS |
| PlateLeT count | **PLT** | 450–1590 | 10^9^/L | 475.33 | 201.33 | 1 | 721.50 | 92.95 | 0.0788 | NS |
| Mean Platelet Volume | **MPV** | 3.8–6.0 | fL | 8.23↑ | 2.08 | 1 | 7.18 ↑ | 0.10 | 0.3409 | NS |
| Platelet Distribution Width | **PDW** | 0.1–30.0 | % | 8.57 | 0.29 | 1 | 7.90 | 0.00 | 0.005 | ****** |
| PlateletCriT | **PCT** | 0.01–9.99 | % | 0.36 | 0.09 | 1 | 0.51 | 0.06 | 0.0416 | ***** |
| Platelet-Large Contrast Ratio | **P-LCR** | 0.1–99.9 | % | 13.60 | 10.65 | 1 | 12.73 | 1.93 | 0.8747 | ***** |

**a)** The arrows indicate the deviations of the index from the reference range. **B)** The asterisk (*) indicates a significant difference between WT and *TertKI* mice. Abbreviations: Abbr. = abbreviation; Ref. = reference; Diff. = difference.

**Supplementary Table 2. Comparison of CA72-4 concentration between WT (C57BL/6) and *TertKI* (G4) mice in serum, stomach, and colon.**

(Young mice: 6 weeks old; Aged mice: 18 months old, *n* = 3)

|  | **Serum (U/mL)** | **Stomach (U/g)** | **Colon (U/g)** |
| --- | --- | --- | --- |
| **Aged WT mice** | 0.28±0.13 | 39.10±0.89 | 28.82±7.27 |
| **Young WT mice** | 0.58±0.27 | 51.10±7.91 | 23.05±5.74 |
| **Aged *TertKI* mice** | 0.50±0.13 | 49.56±0.89 | 16.65±0.51 |
| **Young *TertKI* mice** | 1.56±0.89 | 60.66±5.10 | 32.25±6.76 |

**Supplemental Table 3. A List of PCR primers used for genotyping.**

| **Genotyping** | **Primer (*5****'***→*3****'***)** | **Length (bp)** |
| --- | --- | --- |
| Screening of potentially targeted ES clones | *Neo*_F1: GCTGACCGCTTCCTCGTGCTTTA (87 bp before the *Neo* and *STOP* codon)  *3'Arm3'*_R1: AAGACACCAGTTTCAGCCCAAGTTC (258 bp downstream of *3'Arm*)] | 4858 (targeted); no band (for random integration) |
|  | *EF1α*_F1: GGATCTTGGTTCATTCTCAAGCC  *tACE*_R1: GGACCCTGAGAGAAAGACATACCCAT | 5423 |
|  | *5'Arm*_F1: CAAAGCTGAAAGCTAAGTCTGCAG  *EF1α*_R1: CATAACCCGTAAAGAGGCCAGGC | 579 |
| Detection of chimeras | *5'Arm*_F2: GGTGCTTGCCTTTATGCCTTTA  *EF1α*_R2: ACCACACACGGCACTTACCTGT | 446 |
|  | *STOP*_F: GTTCCGGATCCACTACACCA  *Tert*_R1: CAACAGTAGCATCCATGCACC | 453 |
|  | *Tert*_F1: AAGCTCCCAGAGGCGACAATG  *tACE*_R2: GGCTGGTAAGGGATATTTGCCTG | 563 |
| Detection of F0 *mTert ^flox/+^* heterozygotes after *Neo-Rox* deletion | *5'Arm*_F2: GGTGCTTGCCTTTATGCCTTTA  *EF1α*_R2: ACCACACACGGCACTTACCTGT | 446 |
|  | *STOP*_F: GTTCCGGATCCACTACACCA  *Tert*_R1: CAACAGTAGCATCCATGCACC | 453 |
|  | *Tert*_F1: AAGCTCCCAGAGGCGACAATG (63bp *5'* from STOP)  *3'Arm*_R: AAGACCCAACCAACAGCAGAGA (58 bp into the 3’Arm) | 520 (not 4315 after *Neo-Rox* deletion) |
|  | *Chr1*_F1: GCAGAAGAGGACAGATACATTCAT  *Chr1*_R1: CTACTGAAGAATCTATCCCACAG | 689 |
|  | *Chr1*_F2: CATGCCAATGGTTCACTCTAAGGT  *Chr1*_R2: TCTCTATGTCCCAAAGTGCAGACAC | 334 |
| F1 *mTert ^flox/flox^* homozygotes after *Neo-Rox* deletion | *Tert*_F1: AAGCTCCCAGAGGCGACAATG  *3'Arm*_R: AAGACCCAACCAACAGCAGAGA | 520 (Roxed); 4315(original KI) |
|  | *5'Arm*_F3: AGAGTTTAGCCAGCCAGTGGTGGT  *3'Arm*_R: AAGACCCAACCAACAGCAGAGA | 292 (WT); 6170 (Roxed); 9965 (original KI) |
| Detection of *EIIa*-Cre mice | *Cre*_F: GCGGTCTGGCAGTAAAAACTATC  *Cre*_R: GTGAAACAGCATTGCTGTCACTT | 100 |
|  | *Chr3*_F: CTAGGCCACAGAATTGAAAGATCT  *Chr3*_R: GTAGGTGGAAATTCTAGCATCATCC | 324 |
| G0 *EF1α-mTert ^flox/+^* heterozygotes after *STOP-loxP* removal | *EF1α*_F2: CCAGGCACCTCGATTAGTTC  *Tert_R3*: AGTGCGGTAGATCTTCGGGTC | 472 (floxed); 1349 (original KI) |
| G1 to G5 *EF1α-mTert ^flox/flox^* homozygotes after Neo-Rox deletion | *Tert_F1*: AAGCTCCCAGAGGCGACAATG  *3'Arm*_R: AAGACCCAACCAACAGCAGAGA | 520 (Roxed); 4315(original KI) |
|  | *5'Arm*_F3: AGAGTTTAGCCAGCCAGTGGTGGT  *Tert*_R3: AGTGCGGTAGATCTTCGGGTC | 1636 (no 2512) |
|  | *EF1α*_F2: CCAGGCACCTCGATTAGTTC  *Tert*_R3: AGTGCGGTAGATCTTCGGGTC | 472 (floxed); 1349 (original KI) |
| *Neo* probe sequence | *Neo*_F2: AAGGCGATAGAAGGCGATGC  *Neo*_R: TCATCTCACCTTGCTCCTGC | 468 |

**Supplemental Table 4. A list of primers used for RT-qPCR.**

| **Genotyping** | **Primer (*5****'***→*3****'***)** |
| --- | --- |
| *Fgf7* | *Fgf7*_­­­F: TTTGGAAAGAGCGACGACTT  *Fgf7*_­­­R: GGCAGGATCCGTGTCAGTAT |
| *Tert* | *Tert*_F2: GGATTGCCACTGGCTCCG  *Tert*_R2: TGCCTGACCTCCTCTTGTGAC |
| *Terc* | *Terc*_F: CCGCTGTTTTTCTCGCTGAC  *Terc*_R: TCCCACAGCTCAGGTAAGAC |
| *Tnfα* | *Tnfα*_F: GGTCAATCTGCCCAAGTA  *Tnfα*_R: CACCCATTCCCTTCACAG |
| *Il1β* | *Il1β*_­F: CAACCAACAAGTGATATTCTCCATG  *Il1β*_­R: GATCCACACTCTCCAGCTGCA |
| *Collagen I* | *Col1a1*_F: GGTATGCTTGATCTGTATCTG  *Col1a1*_R: AGTCCAGTTCTTCATTGCATT |
| *Collagen III* | *Col3a1*_R: AGCACCTGTTTCTCCCTT  *Col3a1*_F: CTGGTATGAAAGGACACAGAG |
| *Gapdh* | *Gapdh*_F: AATGGTGAAGGTCGGTGTGAAC  *Gapdh*_R: AGGTCAATGAAGGGGTCGTTG |
