# Supplementary material for: Telomerase reverse transcriptase gene knock‐in unleashes enhanced longevity and accelerated damage repair in mice
Source: Aging Cell. 2024 Dec 11;24(4):e14445. doi: 10.1111/acel.14445 (PMC11984681; doi:10.1111/acel.14445)
Supplement: Supplementary file 3 — Figures S1‐S15. [file ACEL-24-e14445-s002.docx]

**Supplementary Figure Legends**

**
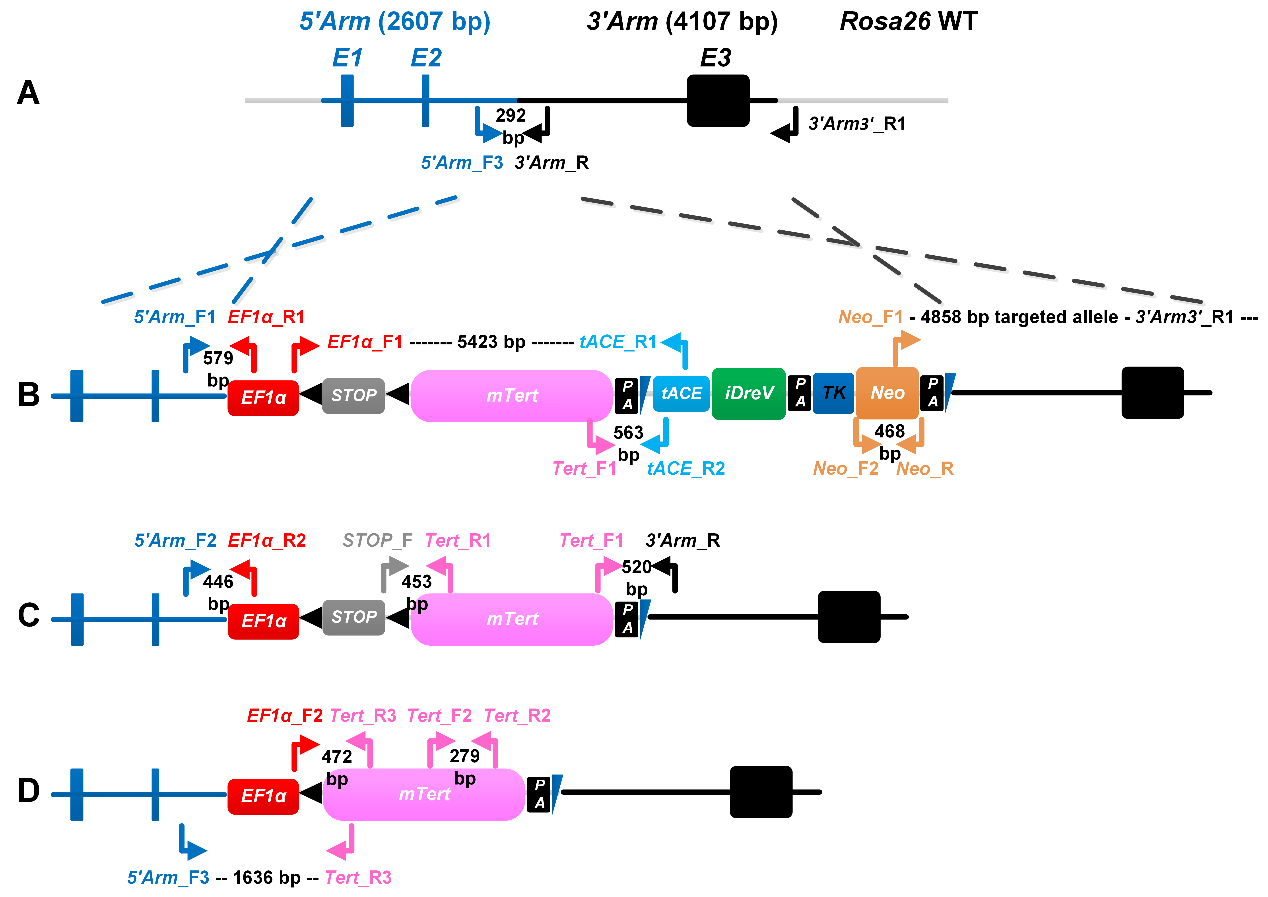
**

**Supplementary Figure 1. Gene targeting strategy for introducing the *EF1α-mTert* transgene into the *Rosa26* locus through homologous recombination.**

**(A)** The WT *Rosa26* locus contains 3 non-coding exons (*E1*–*E3*). The *5'Arm* (colored in blue) for gene targeting is a 2647 bp genomic DNA extending from 240 bp upstream of the *Exon 1* to the first 1162 bp of the *Intron 2*. The *3'*Arm (colored in black) for targeting is 4109 bp *Rosa26* sequence containing 2522 bp of *Intron 2*, *Exon3* and 547 bp of *Intron 3* sequence. The primers *5'Arm_*F3 (*5'*-AGAGTTTAGCCAGCCAGTGGTGGT-*3'*) and *3'Arm*_R (*5'*-AAGACCCAACCAACAGCAGAGA-*3'*) were used to amplify 292 bp PCR product from the WT *Rosa26* allele. The primer *Neo*_F1 (*5'*-GCTGACCGCTTCCTCGTGCTTTA-*3'*) in the targeting construct and the primer *3'Arm3'*_R1 (*5'*-AAGACACCAGTTTCAGCCCAAGTTC-*3'*) which was 258 bp downstream of the *3'*Arm were used to amplify 4858 bp PCR product from genomic DNA of targeted ES clones. (**B)** The TurboKnockout^®^ targeting vector was designed to knock the *EF1α-mTert* transgene into the *Rosa26* gene locus, and contained 2647 bp *Rosa26* *5'*Arm (blue), 1185 bp *EF1α* promoter (red), 969 bp *loxP-STOP-loxP* (grey), 3369 bp mouse *Tert* cDNA (pink), 297 bp HSV *PolyA* signal (PA, black), 151 bp multiple cloning site and *Rox* site (indigo blue), 700 bp mouse testis-specific angiotensin-converting enzyme (*tACE*) promoter (light blue), 1050 bp *iDreV* light-inducible DNA recombinase cDNA (green), 141 bp SV40 polyA signal (black), 604 bp *TK* promoter (dark blue), 835 bp *Neo^r^* gene (orange), 214 bp BGH polyA (black), 159 bp *Neo^r^* -derived sequence and *Rox* site (dark blue), and 4107 bp *Rosa26* *3'Arm* (black). The primers *3'Arm*_F1 (*5'*-CAAAGCTGAAAGCTAAGTCTGCAG-*3'*) and *EF1α*_R1 (*5'*-CATAACCCGTAAAGAGGCCAGGC-*3'*) were used to amplified 579 bp DNA for the confirmation of *5'Arm*-*EF1α* fusion sequence. The primers *Tert*_F1 (*5'*-AAGCTCCCAGAGGCGACAATG-*3'*) and *tACE*_R2 (*5'*-GGCTGGTAAGGGATATTTGCCTG-*3'*) amplifying 563 bp DNA were used to confirmed the *mTert-tACE* fusion. The primers *EF1α*_F1 (*5'*-GGATCTTGGTTCATTCTCAAGCC-*3'*) and *tACE*_R1 (*5'*-GGACCCTGAGAGAAAGACATACCCAT-*3'*) were used to amplify 5423 bp DNA for the *EF1α-mTert-PA* transgene sequence in the targeted ES clones. The presence of ES-derived cells in chimeric mice were confirmed at the DNA level by the presence of 3 PCR products of 446 bp (using *5'Arm*_F2, *5'*-GGTGCTTGCCTTTATGCCTTTA-*3'* and *EF1α*_R2 *5'*-ACCACACACGGCACTTACCTGT-*3'*); 453 bp (using *STOP*_F, *5'*-GTTCCGGATCCACTACACCA-*3'* and *Tert*_R1, *5'*-CAACAGTAGCATCCATGCACC-*3'*), and 563 bp DNA (using *Tert*_F1, *5'*-AAGCTCCCAGAGGCGACAATG-*3'* and *tACE*_R2, *5'*-GGCTGGTAAGGGATATTTGCCTG-*3'*). The primers *Neo*_F2 (*5'*-AAGGCGATAGAAGGCGATGC-*3'*) and *Neo*_R (*5'*-TCATCTCACCTTGCTCCTGC-*3'*) were used to amplify 468 bp *Neo* DNA as a probe to confirm targeted ES clones via Southern Blotting. (**C)** Illustration of anticipated Roxed transgene after self-deletion of the *tACE-iDreV-PA-TK-Neo-PA-Rox* sequence via *iDreV* recombinase during mouse breeding in the absence of G418 (also known as geneticin) selection. This was confirmed by the presence of 520 bp PCR products using primers *Tert*_F1 (*5'*-AAGCTCCCAGAGGCGACAATG-*3'*) and *3'Arm*_R (*5'*-AAGACCCAACCAACAGCAGAGA-*3'*). (**D)** Anticipated constitutive KI allele after removal of the *STOP-loxP* sequence *via* breeding with mice expressing Cre recombinase. Female *Rosa26-mTertKI* mice carrying *loxP-STOP-loxP* were mated with male *EIIa-*Cre mice, resulting in germ-line removal of the *Stop-loxP* sequence and presence of a single *loxP* site between the *EF1α* promoter and *mTert* cDNA. This was confirmed by PCR products of 472 bp floxed DNA using primers *EF1α*_F2 (*5'*-CCAGGCACCTCGATTAGTTC-*3'*) and *Tert*_R3 (*5'*-AGTGCGGTAGATCTTCGGGTC-*3'*), as well as 1636 bp DNA using *5'Arm*_F3 (*5'*-AGAGTTTAGCCAGCCAGTGGTGGT-*3'*) and *Tert*_R3 (*5'*-AGTGCGGTAGATCTTCGGGTC-*3'*) primers. Additionally, primers *Tert*_F2 (*5'*-GGATTGCCACTGGCTCCG-*3'*) and *Tert*_R2 (*5'*-TGCCTGACCTCCTCTTGTGAC-*3'*) were used to carry out quantitative RT-PCR with 279 bp DNA to quantify transgene expression in mouse tissues.


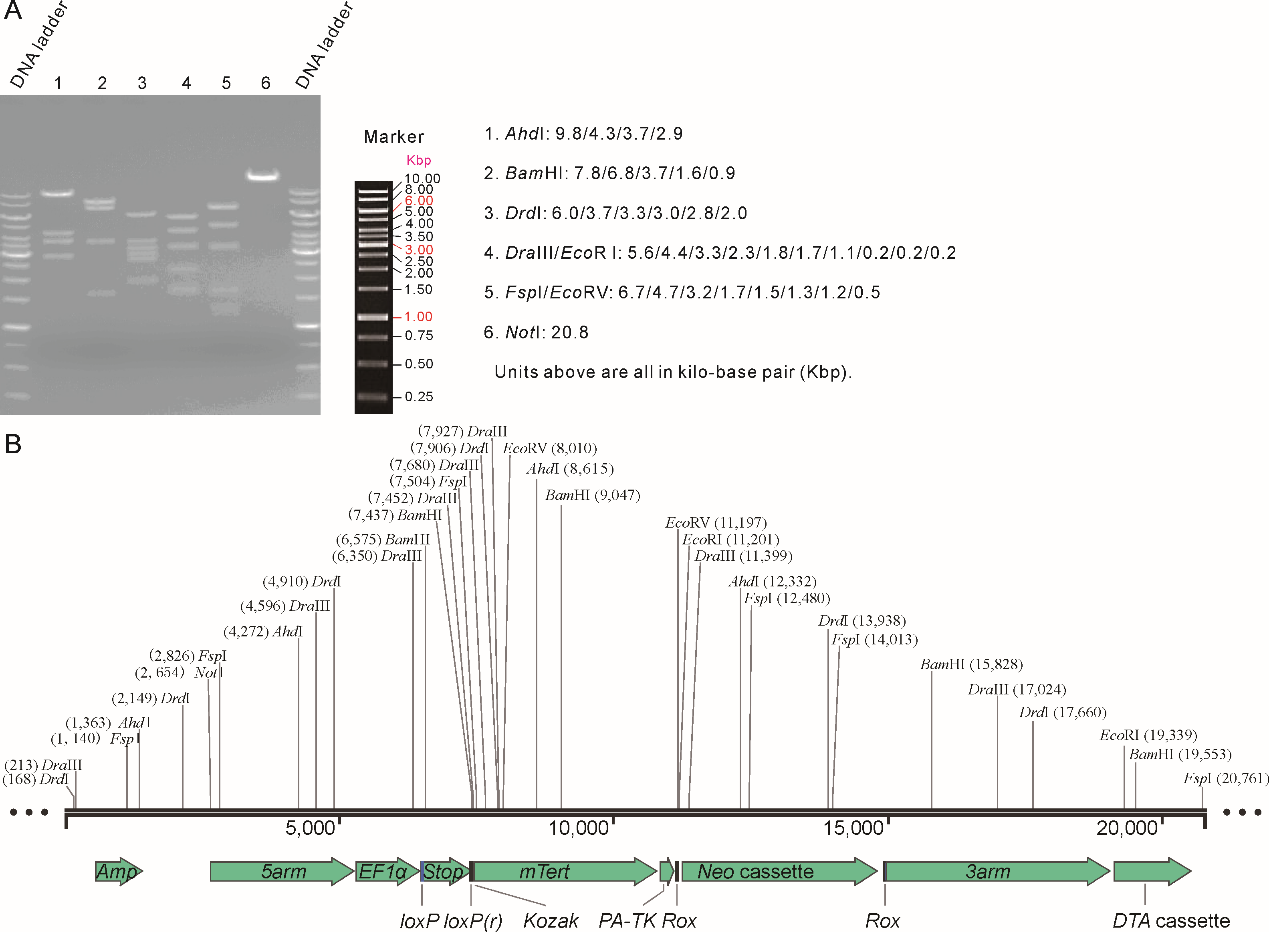


**Supplementary Figure 2. Restriction mapping and linearization of the final *mTert* targeting vector comprising of** ***5'Arm-EF1α-loxP-Stop-loxP-mTert-PA-Rox-tACE-iDreV-PA-TK-Neo-PA-Rox-3'Arm*.**

**(A)** Restriction enzyme digestion analysis was performed on the final vector. The results were as follows: 1. *Ahd*I: 9.8/4.3/3.7/2.9; 2. *Bam*HI: 7.8/6.8/3.7/1.6/0.9; 3. *Drd*I: 6.0/3.7/3.3/3.0/2.8/2.0; 4. *Dra*III/EcoR I: 5.6/4.4/3.3/2.3/1.8/1.7/1.1/0.2/0.2/0.2; 5. *Fsp*I/*Eco*RV: 6.7/4.7/3.2/1.7/1.5/1.3/1.2/0.5; 6. *Not*I: 20.8. The units above represent the fragment sizes in kilobase pairs (Kbp). (**B)** The *Not*I-linearized targeting vector had a length of 20793 bp. This figure provided a schematic representation of the targeted vector design, illustrating the key genetic components involved in the construction of *TertKI* mice. The targeting vector incorporates specific sequences, including *loxP* and *Rox* sites, which were recognized by site-specific recombinases (SSRs) Cre and Dre, respectively. These recombinases are commonly employed in DNA and genome engineering for precise genetic manipulation. The combinatory use of Dre and Cre recombinase-mediated intersectional genetics enhanced the accuracy and specificity of *in vivo* lineage tracing and gene targeting. In the Dre-*Rox* recombination system, two *Rox* sequences were positioned to flank the *tACE-Dre-PA-TK Neo-PA* transgene, enabling its removal and the generation of floxed mice. In the Cre-*loxP* recombination system, the *loxP-STOP-loxP* sequence was placed between the *EF1α* promoter and *mTert* cDNA. The targeting vector included genetic elements of ampicillin resistance gene (*Amp*), *5'Arm*, *EF1α* promoter, *loxP-Stop-loxP, mTert-PA, Rox, tACE-iDreV-PA,* thymidine kinase promoter (*TK*), neomycin resistance gene (*Neo*), *Rox,* *3'Arm* and diphtheria toxin A (*DTA*).


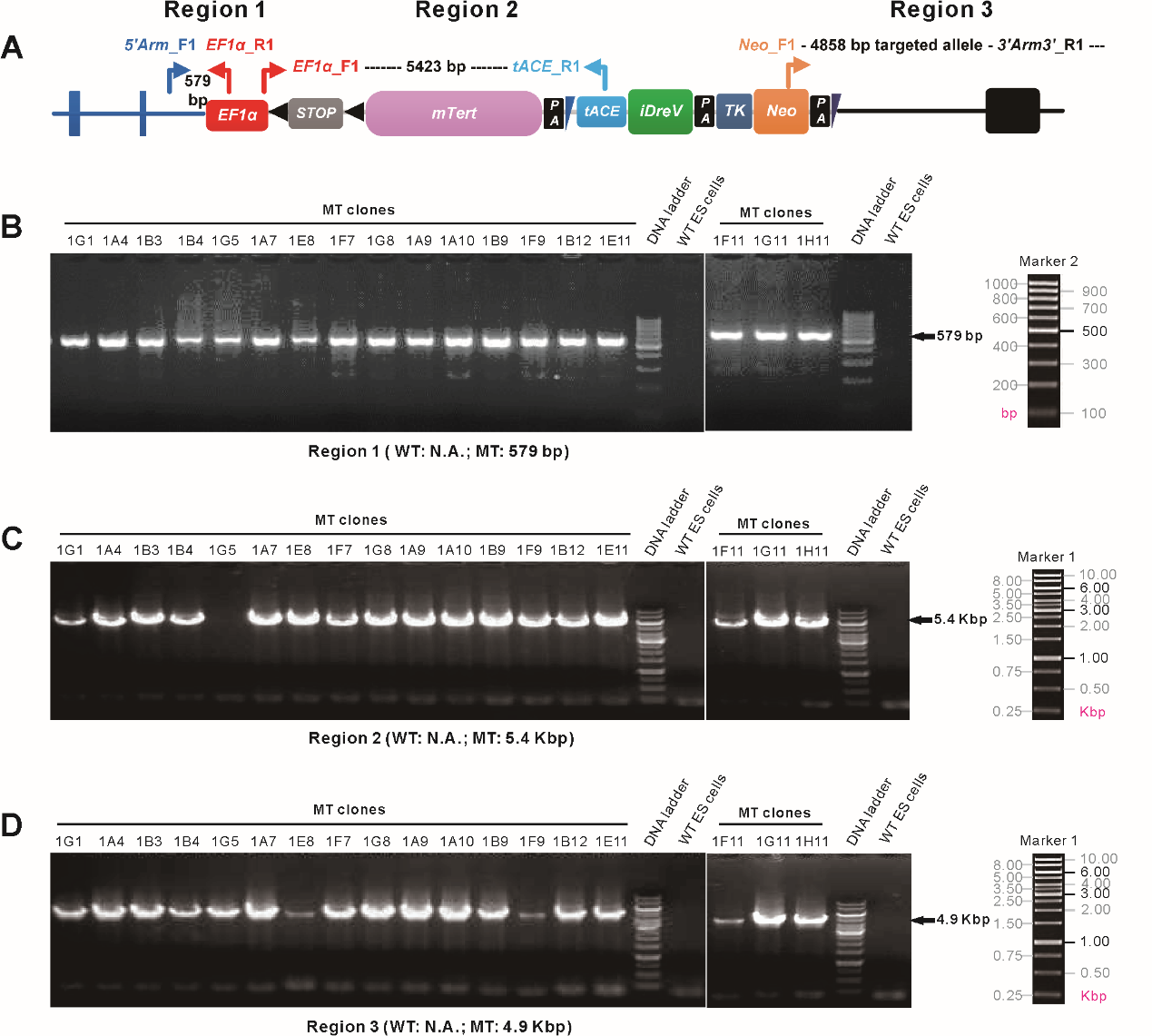


**Supplementary Figure 3. Identification of the targeted ES clones.**

**(A)** The design of PCR strategies for the identification of targeted ES clones. (**B–C)** Genomic DNA were extracted from WT and *Neo^r^* ES clones, and amplified for the region 1 (*5'*Arm_F1: 5’-CAAAGCTGAAAGCTAAGTCTGCAG-*3'*; *EF1α*_R1: *5'*-CATAACCCGTAAAGAGGCCAGGC-*3'*) **(B)** and the region 2 (*EF1α*_F1: *5'*-GGATCTTGGTTCATTCTCAAGCC-*3'*; *tACE*_R1: *5'*-GGACCCTGAGAGAAAGACATACCCAT-*3'*) **(C)**. The successful validation of the *EF1α-loxP-Stop-loxP-mTert-Rox-tACE-iDreV-TK-Neo-Rox* transgene in 17 out of 18 selected ES clones (1G1, 1A4, 1B3, 1B4, 1A7, 1E8, 1F7, 1G8, 1A9, 1A10, 1B9, 1F9, 1B12, 1E11, 1F11, 1G11 and 1H11) was confirmed by the appearance of the expected 579 bp band in region 1 **(B)** and the presence of a 5.4 Kbp fragment in region 2 **(C)**. (**D)** These ES cells were subsequently amplified for from region 3 (*Neo*_F1: *5'*-GCTGACCGCTTCCTCGTGCTTTA-*3'*; *3'Arm3'*_R1: *5'*-AAGACACCAGTTTCAGCCCAAGTTC-*3'*, note that the *3'Arm3'*_R1 primer sequence was not present in the *3'* homology arm, but 258 bp downstream of *3'*Arm). The repeated presence of a 4.9 Kbp DNA in 16 selected ES clones (1G1, 1A4, 1B3, 1B4, 1G5, 1A7, 1F7, 1G8, 1A9, 1A10, 1B9, 1B12, 1E11, 1F11, 1G11 and 1H11) further showed that they were targeted clones with *Neo^r^* knocked into the *Rosa26* locus.


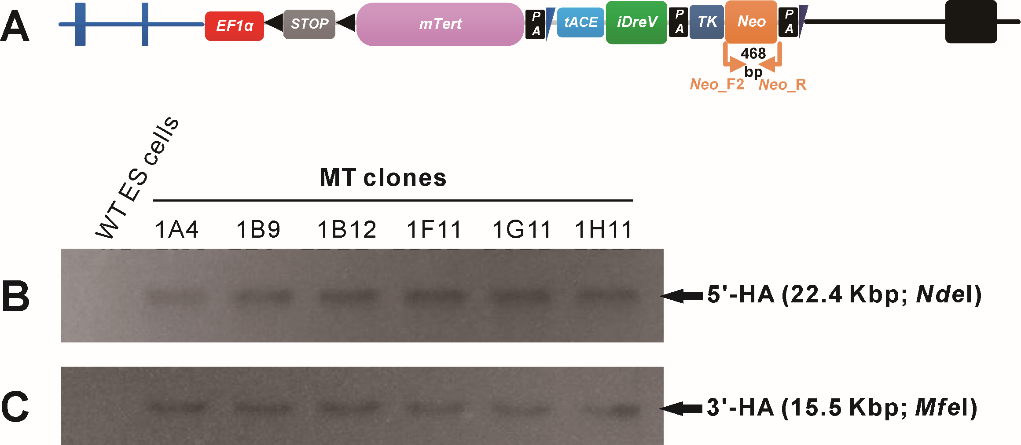


**Supplementary Figure 4. Validation of the PCR-positive targeted ES clones by Southern blot** **analysis.**

**(A)** The design of the Southern blot strategy with *Nde*I and *Mfe*I sites and *Neo* probe (Red box) indicated. Genomic DNA from WT ES cells and six chosen *Neo*-targeted ES clones (1A4, 1B9, 1B12, 1F11, 1G11 and 1H11) were digested with *Nde*I **(B)** or *Mfe*I **(C)** and hybridized with 468 bp *Neo* probe (amplified with *Neo*_F2: *5'*-AAGGCGATAGAAGGCGATGC-*3'*; *Neo*_R: *5'*-TCATCTCACCTTGCTCCTGC-*3'*). (**B)** A 22.4 Kbp positive band was detected in all six clones from *Nde*I digestion as anticipated. (**C)** A 15.5 Kbp fragment was detected in all selected clones, as expected, by the *Neo* probe after *Mfe*I digestion. *3'HA*, *3'*Homology arm; *5'HA*, *5'*Homology arm.


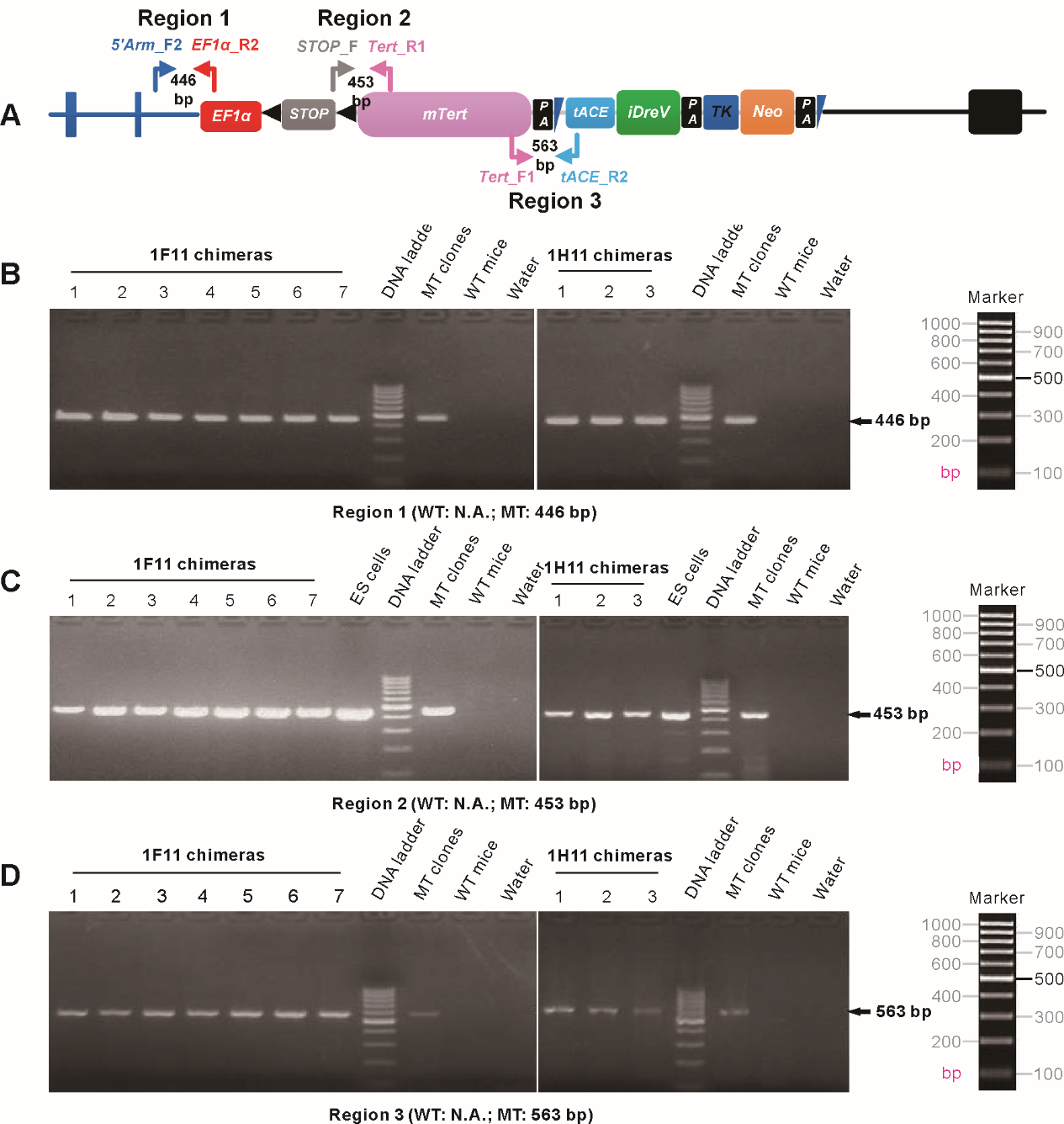


**Supplementary Figure 5. Genotyping strategy to detect chimeric mice with derivatives from targeted ES cells carrying the *EF1α-loxP-Stop-loxP-mTert-PA- Rox-tACE-iDreV-PA- TK-Neo-PA-Rox* transgene.**

**(A)** To facilitate a quick and easy detection of ES cell-derived cells in the mice born after blastocyst injection, three pairs of PCR primers were designed from the *EF1α-loxP-Stop-loxP-mTert-PA- Rox-tACE-iDreV-PA- TK-Neo-PA-Rox* transgene, with expected 446 bp, 453 bp, and 563 bp from the region 1, 2, and 3, respectively. (**B–D)** PCR analysis of 10 pups (including 7 born from microinjection of the 1F11 ES clone and 3 from the 1H11 clone) revealed the presence of 446 bp bands from the regions 1 **(B)**, 453 bp from the region 2 **(C)**, and 563 bp from the region 3 **(D)**, showing that both 1F11 and 1H11 cells had successfully participated in the embryonic development. DNA from the MT clones were used as positive controls, and DNA from WT mice or water were served as negative controls.


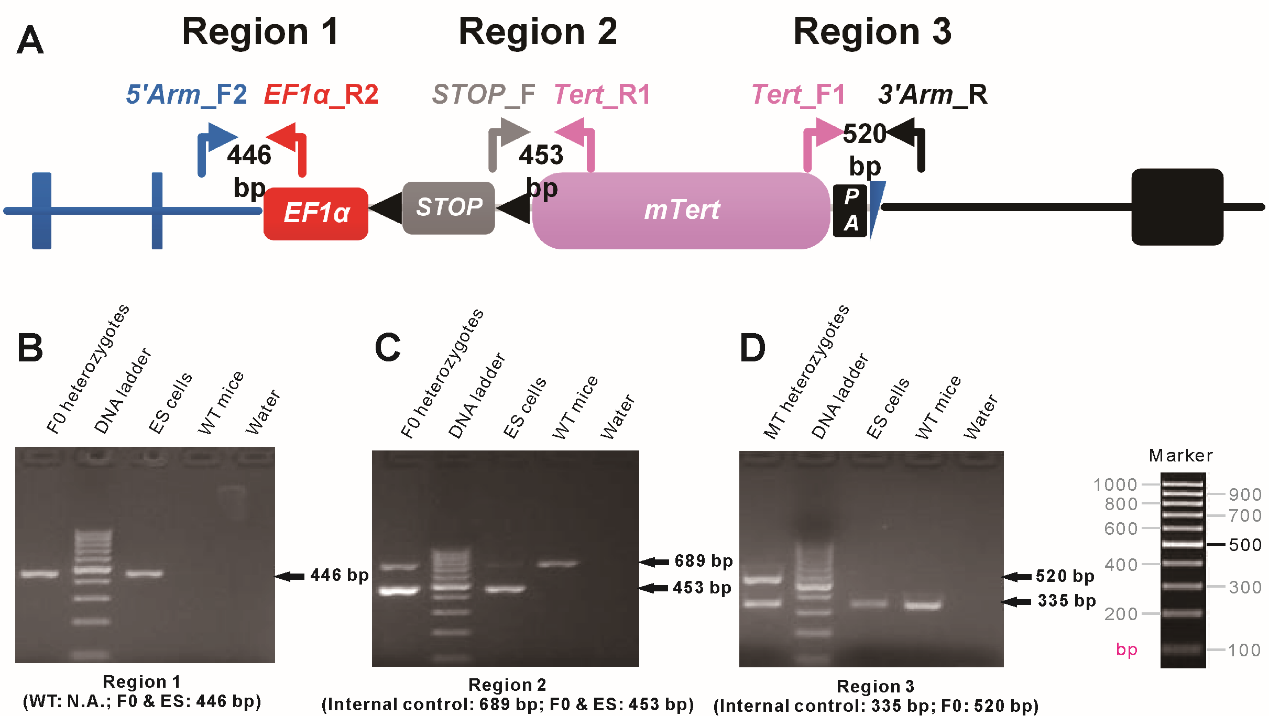


**Supplementary Figure 6 | Genotyping strategy for the removal of *tACE-iDreV-PA-TK-Neo-PA-Rox* in the *EF1α-loxP-Stop-loxP-mTert* ^flox/+^ mice.**

**(A)** Schematic presentation of the knock in allele with deletion of the *tACE-iDreV-PA- TK-Neo-PA-Rox* sequence in the region 3. Three pairs of PCR primers were designed to detect EF1α promoter (*5'Arm*_F2: *5'*-GGTGCTTGCCTTTATGCCTTTA-*3'*; *EF1α*_R2: *5'*-ACCACACACGGCACTTACCTGT-*3'*) from the region 1 (446 bp), the *mTer*t (*STOP*_F: *5'*-GTTCCGGATCCACTACACCA-*3'*, *Tert*_R: *5'*-CAACAGTAGCATCCATGCACC-*3'*) from the region 2 (453 bp) and 520 bp PCR product with *Tert*_F1 (*5'*-AAGCTCCCAGAGGCGACAATG-*3'*) and *3'Arm*_R (*5'*-AAGACCCAACCAACAGCAGAGA-*3'*) from the region 3. Two pairs of PCR primers were designed from Chromosome 1 (689 bp from *Chr1*_F1: *5'*-GCAGAAGAGGACAGATACATTCAT-*3'* and *Chr1*_R1: *5'*-CTACTGAAGAATCTATCCCACAG-*3'*; 334 bp from *Chr1*_F2: *5'*-CATGCCAATGGTTCACTCTAAGGT-*3'* and *Chr1*_R2: *5'*-TCTCTATGTCCCAAAGTGCAGACAC-*3'*) as internal controls. (**B)** PCR with *5'Arm*_F2 and *EF1α*_R2 primers detected 446 bp *EF1α* transgene in the targeted ES cells and F0 heterozygote, but not in WT mice. (**C)** PCR with *STOP*_F and *Tert*_R primers detected 453 bp of *Tert* transgene in the targeted ES cells and F0 heterozygote, and 689 bp band from all samples. (**D)** While PCR with *Chr1*_F2 and *Chr1*_R2 showed the 335 bp band across all samples as an internal positive control, PCR with *Tert_*F1 and *3'Arm*_R primers revealed specific 520 bp DNA only from the targeted ES cells and MT heterozygote (instead of 4315 bp prior to deletion), showing successful removal of the *tACE-iDreV-PA- TK-Neo-PA-Rox* sequence from the transgene.


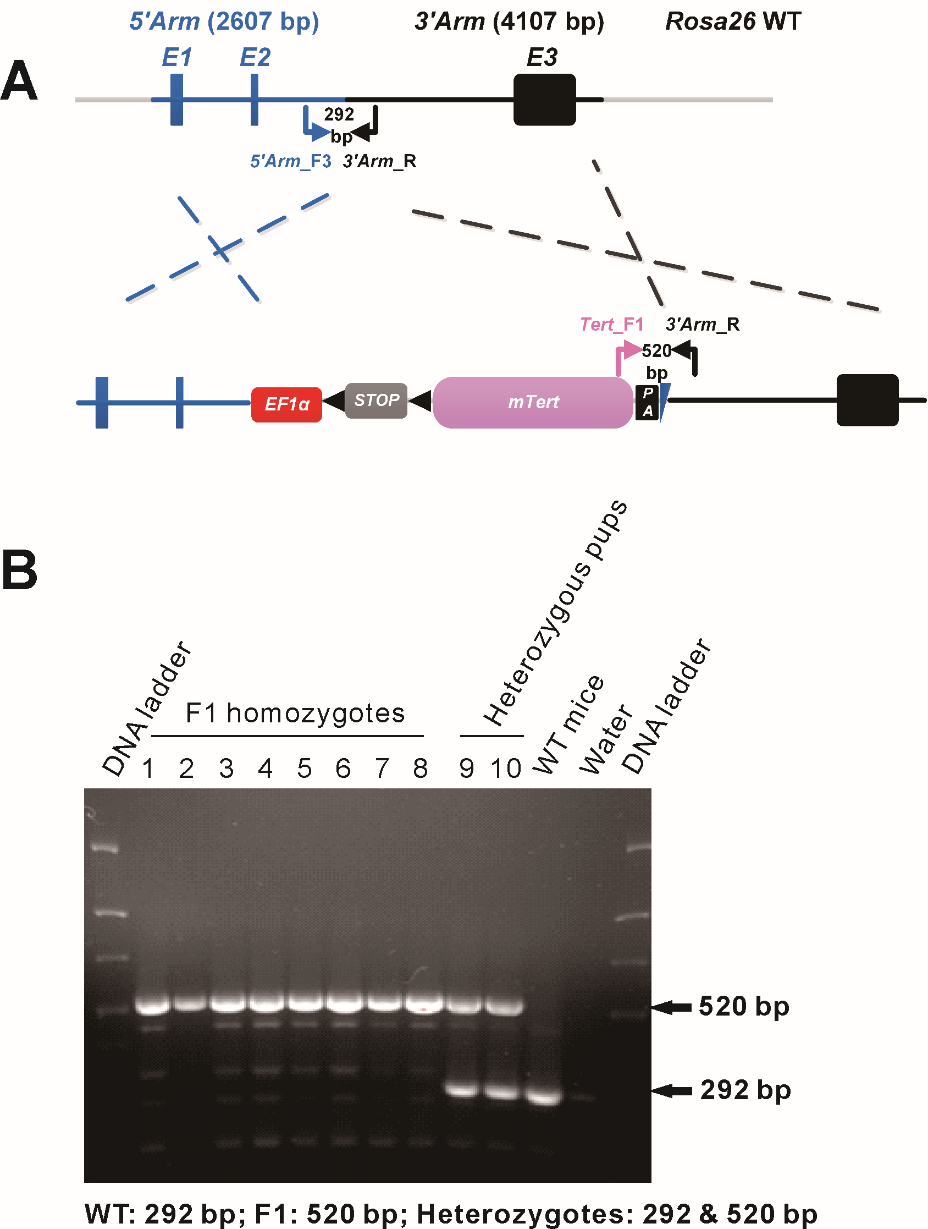


**Supplementary Figure 7. Genotyping strategy for *EF1α-loxP-Stop-loxP-mTert ^flox/flox^* homozygous mice.**

**(A)** The *Tert*_F1 and *3'Arm*_R primers were used to detect 520 bp DNA from the *Tert* knock-in allele, and primers *5'Arm*_F3 (*5'*-AGAGTTTAGCCAGCCAGTGGTGGT-*3'*) and *3'Arm*_R (*5'*-AAGACCCAACCAACAGCAGAGA-*3'*) were used to amplify 292 bp from the WT *Rosa26* allele. (**B)** While 292 bp DNA was amplified from one WT and two heterozygotes, only 520 bp PCR product was amplified from eight F1 mice (No. 1–8), demonstrating they were homozygous knock-in for the *EF1α-loxP-Stop-loxP-mTert ^flox/flox^* transgene.


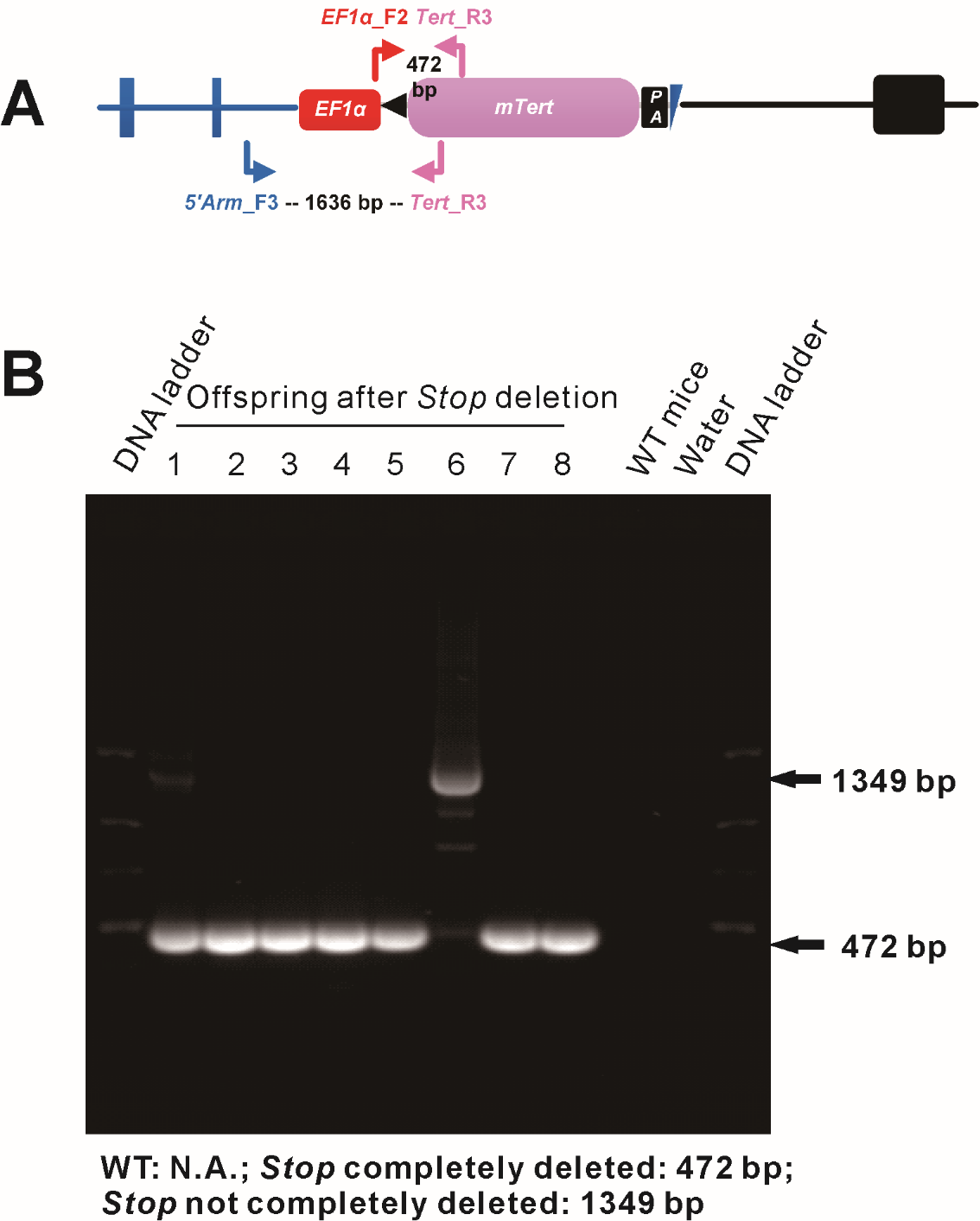


**Supplementary Figure 8. Genotyping strategy for detection of *EF1α- mTert ^flox/+^* mice after *Stop* deletion.**

**(A)** The loxP-STOP-loxP sequence was placed between the *EF1α* promoter and *mTert* cDNA. *EF1α-loxP-Stop-loxP-mTert ^flox/flox^* mice were mated with EIIa-Cre transgenic mice. Offspring were genotype with primers *EF1α*_F2 (*5'*-CCAGGCACCTCGATTAGTTC-*3'*) and *Tert*_R3 (*5'*-AGTGCGGTAGATCTTCGGGTC-*3'*) which amplified 472 bp DNA from floxed allele and 1349 bp DNA from the original inactive transgene. (**B)** While mouse 6 showed a band of 1349 bp, the other 7 pups (2, 3, 4, 5, 7 and 8) displayed a single PCR product of 472 bp, showing that they were floxed mice with *STOP-loxP* removed between the *EF1α* promoter and *mTert* cDNA to facilitate *mTert* overexpression. In pup 1, a weak band at 1349 bp and a strong band at 472 bp were detected, suggesting that the *Stop* gene was partially deleted in the pup 1.


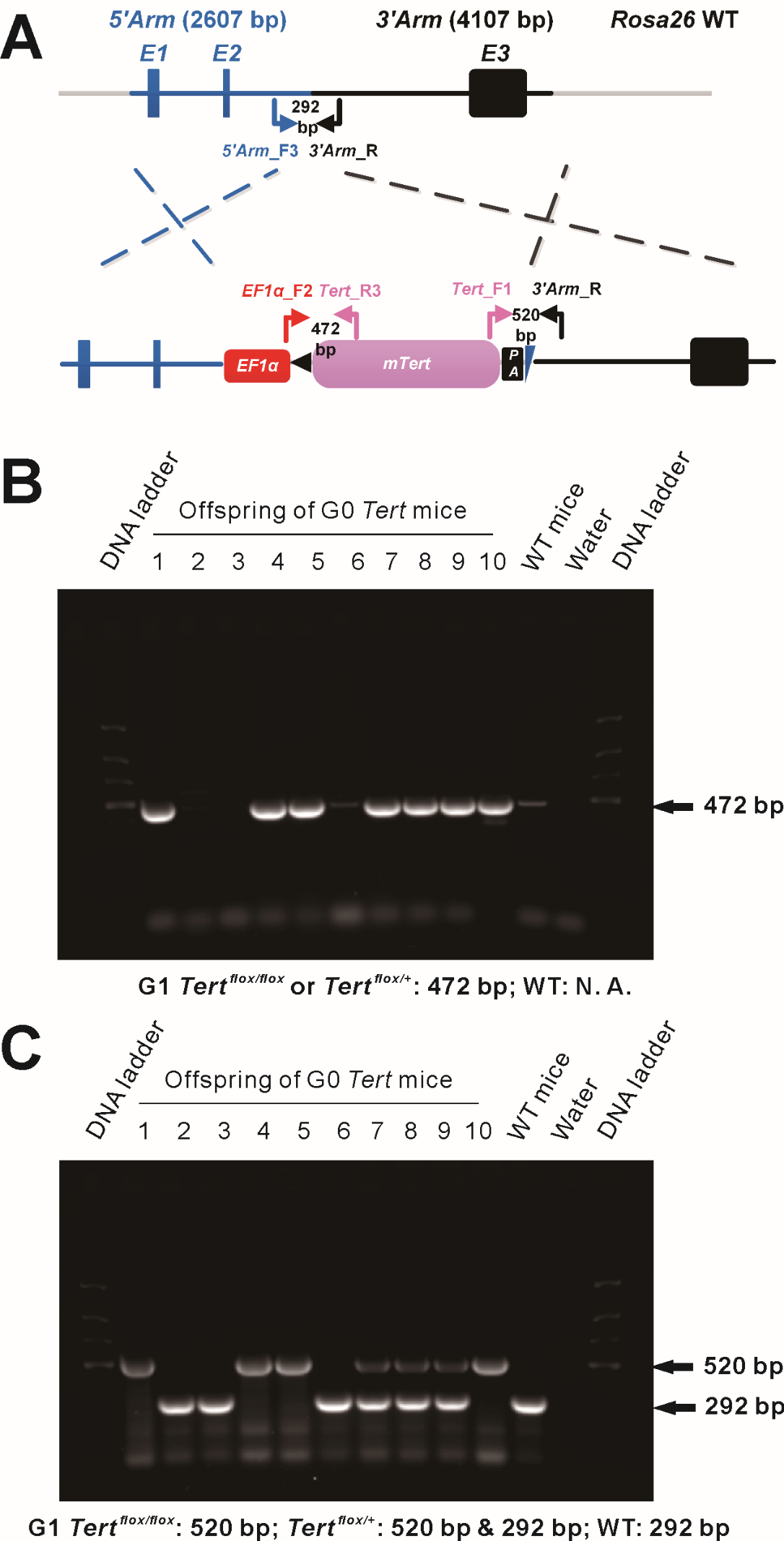


**Supplementary Figure 9. Genotyping strategy for detection of *EF1α-mTert ^flox/flox^* homozygous mice.**

**(A)** Three pairs of primers were used to determine the genotype of *EF1α-mTert ^flox/flox^* mice after *Stop* deletion: (1) Primers *5'Arm*_F3 and *3'Arm*_R to display WT *Rosa26* allele with 292 bp product; (2) primers *Tert_F1* and *3'Arm*_R to show removal of *Neo^r^* with 520 bp DNA, and primers *EF1α*_F2 and *Tert*_R3 with 473 bp band for the floxed transgene. (**B)** The absence of the 472 bp floxed DNA in mouse 2/3/6 confirmed their WT nature, while mouse 1/4/5/7/8/9/10 carried at least one copy of the floxed transgene. **(C)** PCR amplification with two pairs of primers (*5'Arm*_F3 and *3'Arm*_R for 292 bp WT; and *Tert*_F1 and *3'Arm* for 520 bp transgene) in the same PCR reactions showed that pup 2, 3 and 6 and were WT mice as they showed a 292 bp only; pups 7, 8 and 9 were heterozygous with both 520 bp and 292 bp PCR products, and offspring 1, 4, 5 and 10 were homozygous for the *EF1α-mTert ^flox/flox^* as they displayed a 520 bp fragment, with the absence of 292 bp DNA from the WT *Rosa26*.


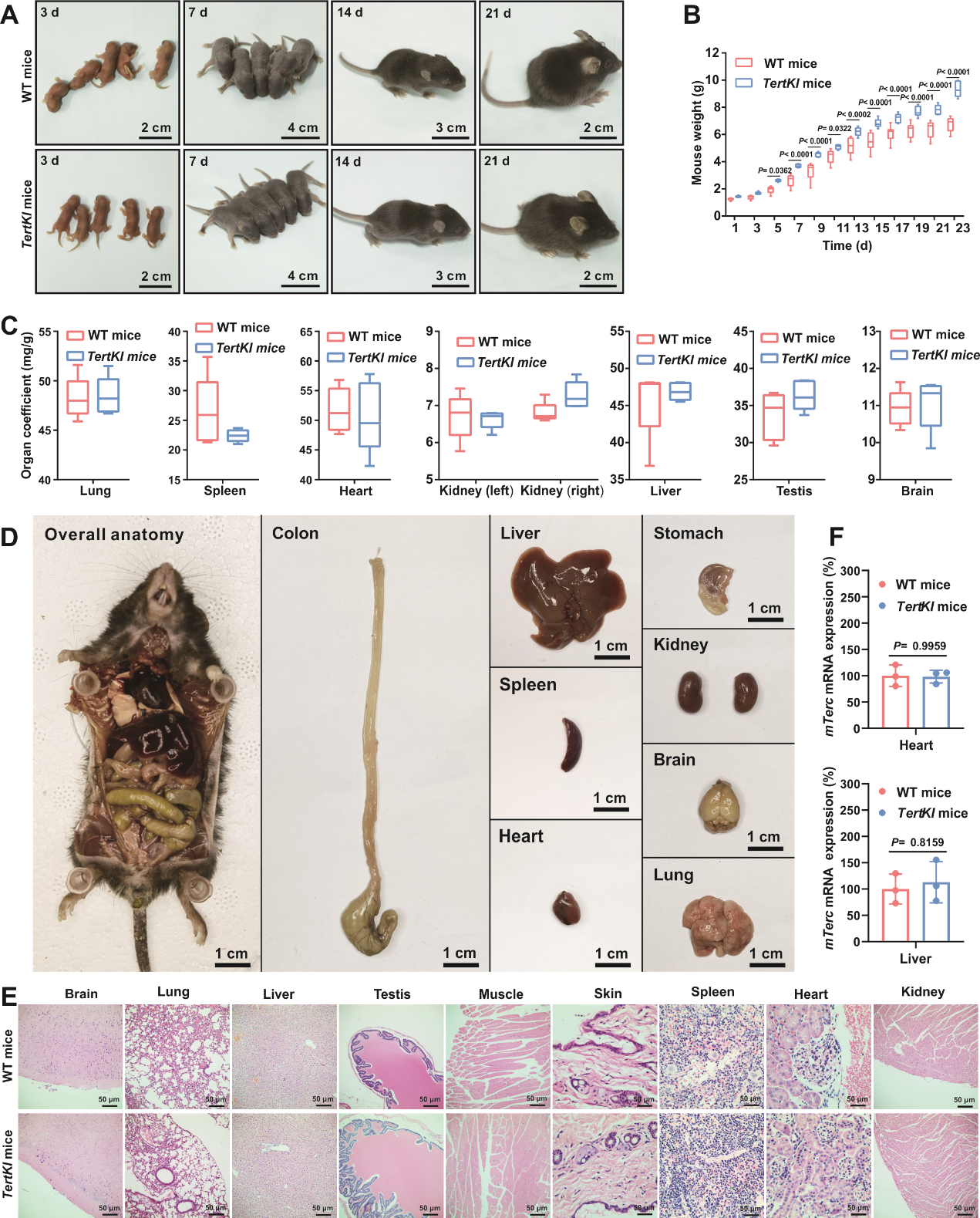


**Supplementary Figure 10. Phenotypic and histopathological analysis of *TertKI* mice compared to WT mice.**

**(A)** Phenotypic characteristics showed comparable appearance, response to a stimulus, behavior, and activity, between *TertKI* (G2) and WT mice (C57BL/6), both 6 months old. We conducted a comparative analysis between *TertKI* mice and WT mice, employing visual observations and photographic assessments. Our findings did not indicate noteworthy disparities between the two groups, encompassing attributes such as appearance, body size and other visible characteristics. These observations, to a certain extent, lend support to our conclusion that *TertKI* mice exhibit phenotypic characteristics akin to those of WT mice. (**B)** *TertKI* mice exhibited a noticeable weight gain during 5–23 days compared to WT mice. Data represent mean ± SD for more than three independent experiments. (**C)** Organ coefficients, reflecting organ size relative to body weight, were consistently comparable between *TertKI* and WT mice. (**D)** Autopsy findings in deceased *TertKI* mice. Autopsies were conducted on 98 deceased *TertKI* mice, examining major organs such as the colon, liver, spleen, heart, stomach, kidneys, brain, and lungs. No significant indications of abnormal organ tissue hyperplasia or tumor growth were noted, indicating a lack of pathological changes associated with organ tissue hyperplasia or tumorigenesis in the investigated *TertKI* mouse models. **(E)** Histopathological examination of selected tissues did not show any abnormalities in *TertKI* mice compared to WT mice. These results demonstrate that *Tert*-overexpression did not lead to significant phenotypic differences or histopathological abnormalities. Statistical differences are represented on the graph, with *p* <0.05 indicating a significant difference and *p* < 0.01 representing a highly significant difference. **(F)** *mTerc* expression in WT and *TertKI* mice in heart and liver. G12 *TertKI* and WT (C57BL/6) mice were about 6 months old (*n*= 3).

**
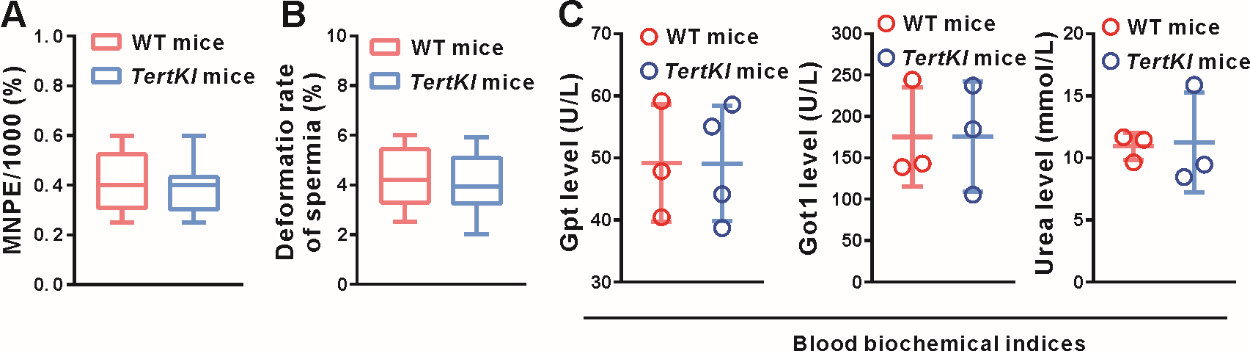
**

**Supplementary Figure 11. Assessment of teratogenic and genotoxic effects in *TertKI* mice.**

**(A)** Micronucleated polychromatic erythrocytes (MPE) assay showed no significant increase in MPE frequency in *TertKI* mice compared to WT mice. (**B)** Deformation rates of sperm were comparable between *TertKI* and WT mice. (**C)** Blood biochemical indices including Serum Glutamic-Pyruvic Transaminase (Gpt) for liver function, glutamic-oxaloacetic transaminase (GOT1) as a prognostic marker for pancreatic ductal adenocarcinoma, urea level for kidney function in *TertKI* mice showed negligible impact, and all indices were within the normal range with no significant alteration. These results suggest that *Tert*-overexpression did not induce teratogenic or genotoxic effects, and had insignificant influence on blood biochemical parameters in *TertKI* mice.


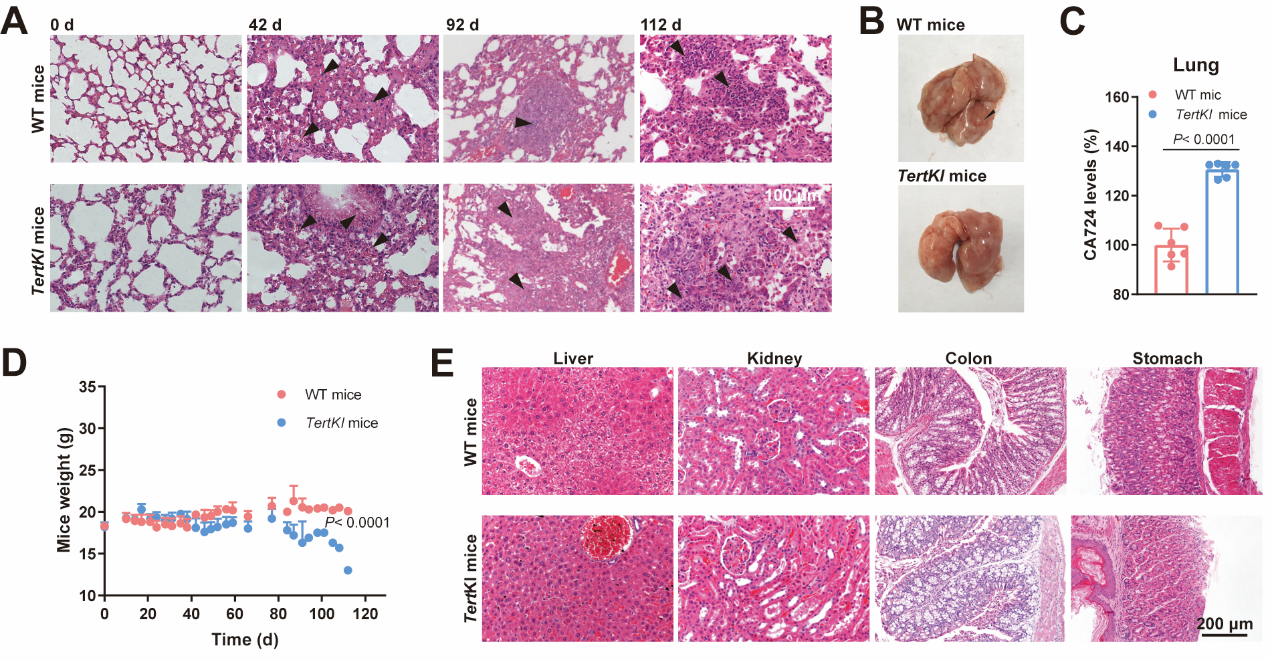


**Supplementary Figure12. Urethane-induced lung cancer progression in TertKI and WT mice.**

**(A)** Histopathological analysis of lungs from WT (C57BL/6) and *TertKI* (G8) mice treated with urethane for 0, 42, 92, and 112 days. **(B)** Morphological examination of lungs from WT and *TertKI* mice treated with urethane for 92 days post-execution. **(C)** Relative CA72-4 levels in lungs of WT and *TertKI* mice treated with urethane for 112 days post-execution. **(D)** Body weight measurements of WT and *TertKI* mice treated with urethane. **(E)** Histopathological analysis of liver, kidney, colon, and stomach from WT and *TertKI* mice treated with urethane for 112 days.

**
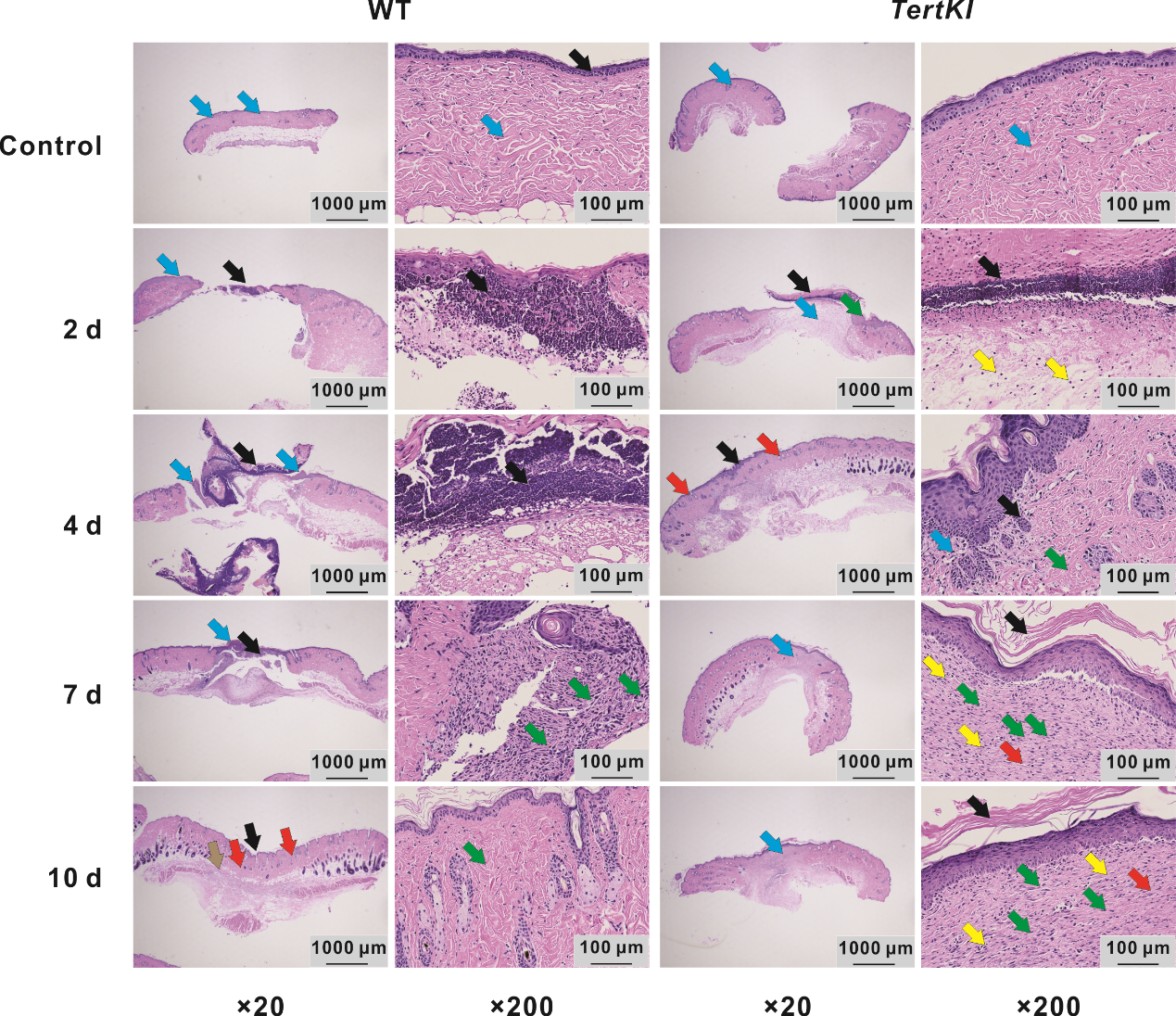
**

**Supplementary Figure 13. Histological evaluation of wound epidermis healing quality *via* H&E-stained sections.**

**For WT mice** (C57BL/6, over 8 weeks old), 1) Control (Before injury): The epidermis displayed a smooth surface with inconspicuous keratinization, and tightly arranged epidermal cells (black arrow). Abundant collagen fibers in the dermal layer with large, intricately woven collagen networks (cyan arrow). No apparent infiltration of inflammatory cells. **2)** 2 Days after injury: Epidermal necrosis was observed, accompanied by a significant presence of purulent cells in the underlying tissue (black arrow). Intense epidermal thickening (cyan arrow) on both sides, characterized by disordered basal cell proliferation. Loss of tissue integrity in the underlying layers. **3)** 4 Days after injury: Epidermal necrosis was prominent, leading to the formation of purulent scabs, with an abundance of purulent cells in the underlying tissue (black arrow). Pronounced epidermal thickening (cyan arrow) on both sides. Loss of tissue integrity in the underlying layers. **4)** 7 Days after injury: New epidermal formation was observed at the injury site with extensive epidermal thickening (black arrow). Presence of necrotic material and incomplete keratinization on the surface (cyan arrow), causing separation from the underlying tissue. Some localized proliferation of granulation tissue was seen on one side, along with the emergence of new blood vessels and fibroblast cells (green arrow), characterized by relatively disorganized arrangements. Both sides exhibited considerable proliferation of fine collagen fibers (green arrow) with relatively orderly arrangements. **5)** 10 Days after injury: Improved tissue repair at the injury site, with new epidermis closely resembling the surrounding normal epidermis (black arrow). Abundant collagen fibers in the dermal layer with large, intricately woven collagen networks (green arrow). Presence of numerous new hair follicles and sebaceous glands (red arrow). Considerable repair in the muscle layer, although many original muscle cells were damaged and disappeared (brown arrow). **For *TertKI* mice** (G3, over 8 weeks old), **1)** Control (Before injury): The tissue's epidermis appeared relatively flat, with inconspicuous keratinization and tightly packed epidermal cells. The dermal layer displayed rich collagen fiber content, with thick collagen fibers interwoven into a dense network (indicated by cyan arrows), and no significant infiltration of inflammatory cells was observed. **2)** 2 Days after injury: Extensive necrosis was evident in the local area, with the loss of the normal epidermal structure. The surface was covered with a substantial amount of purulent scab (black arrow), and the underlying tissue exhibited edema (cyan arrow) and a loose structure. Some neutrophil infiltration was observed (yellow arrow), and the epidermis was thickened on both sides (green arrow). **3)** 4 Days after injury: The tissue at the injury site showed relatively good repair. New epidermis was visible (black arrow), with increased thickness and occasional elongated epidermal protrusions. Some blood vessels were observed in the sub-epidermal layer (cyan arrow). The dermal layer had rich collagen fiber content, with thick collagen fibers forming an interwoven mesh (green arrow), and a few newly formed hair follicles were visible (red arrow). **4)** 7 Days after injury: The tissue showed signs of repair, with the appearance of new epidermis that was relatively thick and exhibited mild keratinization (black arrow). There were no epidermal protrusions, but substantial growth of granulation tissue in the dermal layer (cyan arrow), characterized by increased blood vessels and fibroblast proliferation (green arrow). Fibroblasts displayed orderly alignment parallel to the epidermis, and fine collagen fibers were neatly arranged (red arrow). Scattered infiltration of inflammatory cells was also observed (yellow arrow). **5)** 10 Days after injury: The tissue repair at the injury site was evident. The new epidermis was thicker with mild to moderate keratinization (black arrows), and no epidermal protrusions were observed. The dermal layer showed proliferative granulation tissue (cyan arrows), featuring an abundance of newly formed blood vessels and fibroblast cells (green arrows). Fibroblast cells exhibited an orderly arrangement parallel to the epidermis, and thin collagen fibers were neatly aligned (red arrows). Additionally, there was scattered infiltration of inflammatory cells (yellow arrows).

**
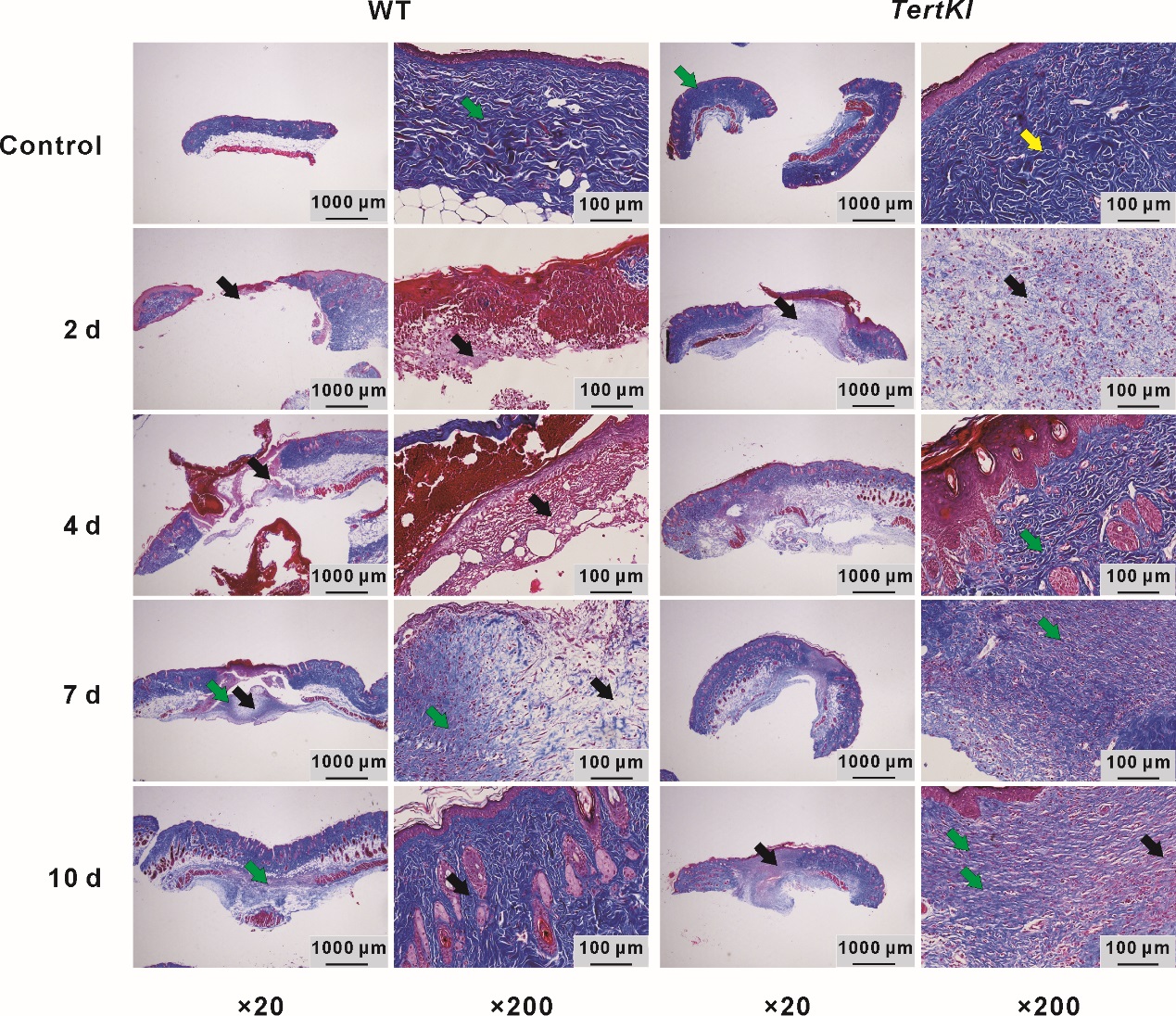
**

**Supplementary Figure 14. Enhanced histological assessment of collagen deposition and restructuring in wound dermal tissue using Masson staining sections.**

**For WT mice** (C57BL/6, over 8 weeks old), **1)** Control (Before injury): Abundant collagen fibers in the dermal layer with large, intricately woven collagen networks (green arrow). No apparent signs of damage or injury. **2)** 2 Days after injury: Collagen fibers at the injury site had disappeared (black arrow). **3)** 4 Days after injury: Collagen fibers at the injury site had disappeared (black arrow). **4)** 7 Days after injury: In the central part of the injury site, there was a loose structure with relatively sparse collagen fiber proliferation (black arrows), while on both sides, there was more pronounced proliferation of fine collagen fibers (green arrows) with a relatively organized arrangement. **5)** 10 Days after injury: Abundant collagen fibers in the dermal layer with large, intricately woven collagen networks (black arrow). At the site of muscle layer damage, there was a noticeable presence of fine collagen fibers (green arrow) with relatively uniform arrangement, displaying a lighter coloration compared to normal collagen fibers. **For *TertKI* mice** (G3, over 8 weeks old), **1)** Control (Before injury): Abundant collagen fibers in the dermal layer with large, intricately woven collagen networks (green arrow). No apparent damage. **2)** 2 Days after injury: Large collagen fibers at the injury site disappeared. Some fine collagen fibers were increased sporadically (black arrow). Collagen fiber arrangement was extremely disordered. **3)** 4 Days after injury: Excellent remodeling of dermal collagen fibers at the injury site. Thick collagen fibers were woven into a network (green arrow). **4)** 7 Days after injury: Newly formed collagen fibers were finer (green arrow), abundant, and well-organized. No other significant abnormalities were observed. **5)** 10 Days after injury: Newly formed collagen fibers were finer (green arrow), well-organized, thicker at both ends (black arrow), and thinner in the middle. No other significant abnormalities were observed.


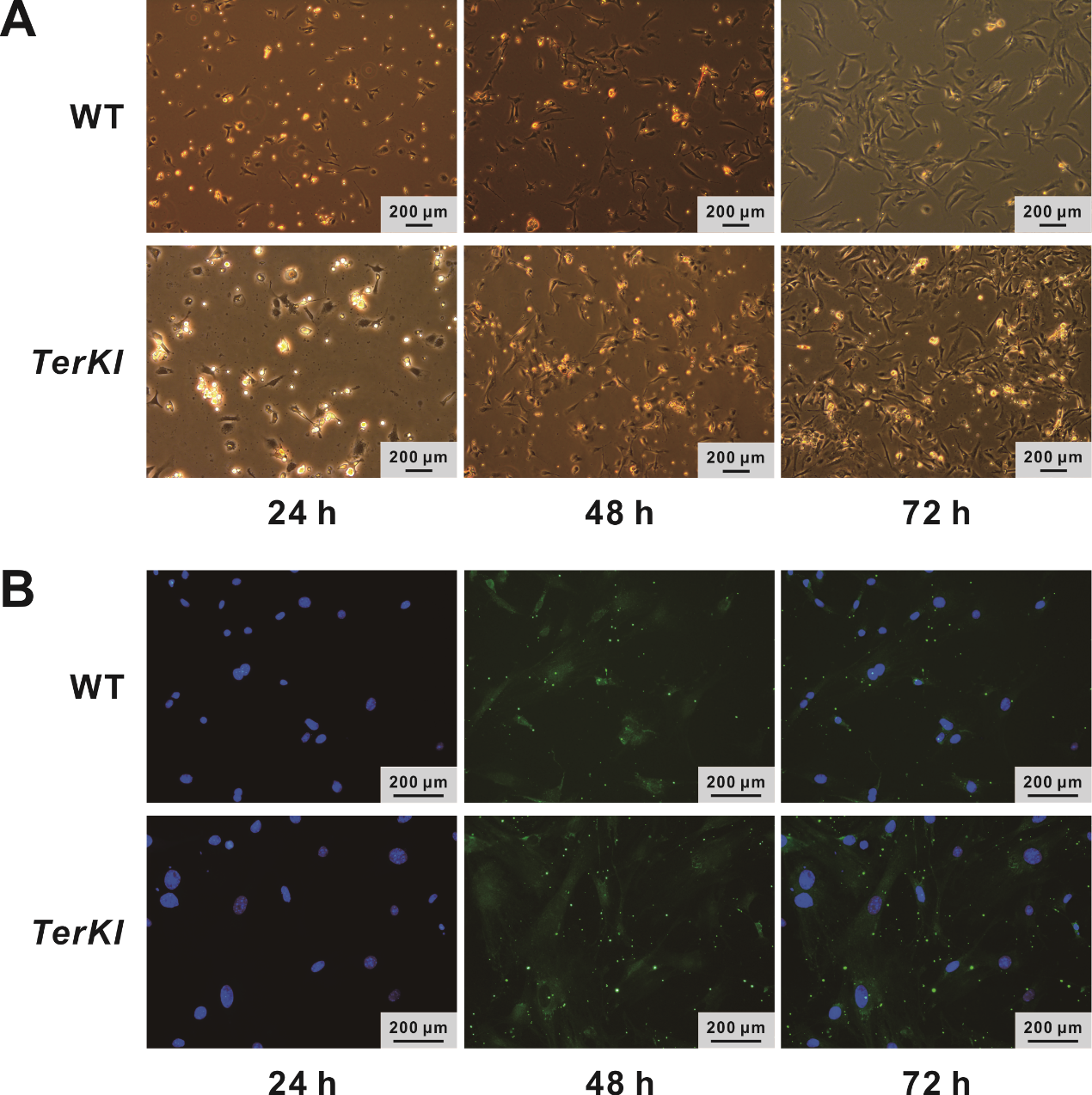


**Supplementary Figure 15. Characterization of mouse skin fibroblasts.**

**(A)** Microscopic images (magnification × 100) depicting skin fibroblasts from both WT and *TertKI* mice. Large quantities of highly active neonatal mouse fibroblasts were obtained using an enzyme digestion method. After the initial 24 hours of culture, only a few cells were adhered to the surface, with many larger tissue blocks remaining unattached. After 48 hours of culture, mouse skin fibroblasts began to attach, displaying a spindle or irregular triangular shape, with a few epithelial cells present. The cell nuclei were clear, and no colonies were observed. After 72 hours of culture, the cells grew into a monolayer with typical spindle shapes, often forming vortex-like or crisscross patterns. The confluence rate for WT group cells was approximately 40%, while *TertKI* mouse fibroblasts achieved a confluence rate of around 60%. (**B)** Identification of skin fibroblasts from WT and *TertKI* mice using immunofluorescent staining of Vimentin (× 200). To identify the isolated mouse skin fibroblasts, immunofluorescence staining for Vimentin, a cytoskeletal protein, was conducted. Results revealed high expression of Vimentin within the mouse skin cells, indicated by green fluorescence, distributed around the cell nuclei stained with DAPI (4',6-diamidino-2-phenylindole appearing as blue fluorescence). The expression rate of green fluorescence Vimentin in isolated WT and *TertKI* mouse skin fibroblasts exceeded 95%.
